# Spatial arrangements of cyclodextrin host–guest complexes in solution studied by ^13^C NMR and molecular modelling

**DOI:** 10.3762/bjoc.20.33

**Published:** 2024-02-20

**Authors:** Konstantin Lebedinskiy, Ivan Barvík, Zdeněk Tošner, Ivana Císařová, Jindřich Jindřich, Radim Hrdina

**Affiliations:** 1 Department of Organic Chemistry, Faculty of Science, Charles University, Hlavova 8, 128 43 Praha, Czech Republic; 2 Institute of Physics, Faculty of Mathematics and Physics, Charles University, Ke Karlovu 2026/5, 121 16 Praha, Czech Republic; 3 Department of Inorganic Chemistry, Faculty of Science, Charles University, Hlavova 8, 128 43 Praha, Czech Republic

**Keywords:** anisotropy, ^13^C NMR, cyclodextrin, host–guest complexes

## Abstract

^13^C NMR spectroscopic analyses of *C_s_* symmetric guest molecules in the cyclodextrin host cavity, combined with molecular modelling and solid-state X-ray analysis, provides a detailed description of the spatial arrangement of cyclodextrin host–guest complexes in solution. The chiral cavity of the cyclodextrin molecule creates an anisotropic environment for the guest molecule resulting in a splitting of its prochiral carbon signals in ^13^C NMR spectra. This signal split can be correlated to the distance of the guest atoms from the wall of the host cavity and to the spatial separation of binding sites preferred by pairs of prochiral carbon atoms. These measurements complement traditional solid-state analyses, which rely on the crystallization of host–guest complexes and their crystallographic analysis.

## Introduction

Complexation of organic and inorganic compounds with α-, β-, or γ-cyclodextrins and their derivatives [1] is an established tool used in medicine for drug delivery [2–4], in analytical and preparative chemistry for compound separation [5] and in materials science for small molecule detection [6–7]. Association (binding) constants between the host and guest molecules [8–10] are typically measured by ^1^H NMR titration [11–12] or isothermal titration calorimetry [13]. Single crystals for many host–guest complexes have been prepared, and their structure elucidated by X-ray crystallography [14–15]. Conformations of host–guest complexes in solution have been studied by 2D NMR experiments [11] (NOESY, ROESY) or proposed computationally [16–17] based on dispersion forces and hydrogen bonding between the cyclodextrin (CD) unit and the guest molecule. Determination of the ee of chiral guests was achieved by observing the splitting of ^1^H NMR signals of the achiral host upon formation of diastereomeric inclusion complexes [18–19]. Shifting the H-3 and H-5 proton signals of CDs in ^1^H NMR or 2D NMR ROESY or NOESY experiments can indicate the spatial distances between host and guest atoms and distinguish between enantiomeric guests. However, the evaluation of such interactions is often hampered by overlapping signals [20].

## Results and Discussion

**Figure 1:**
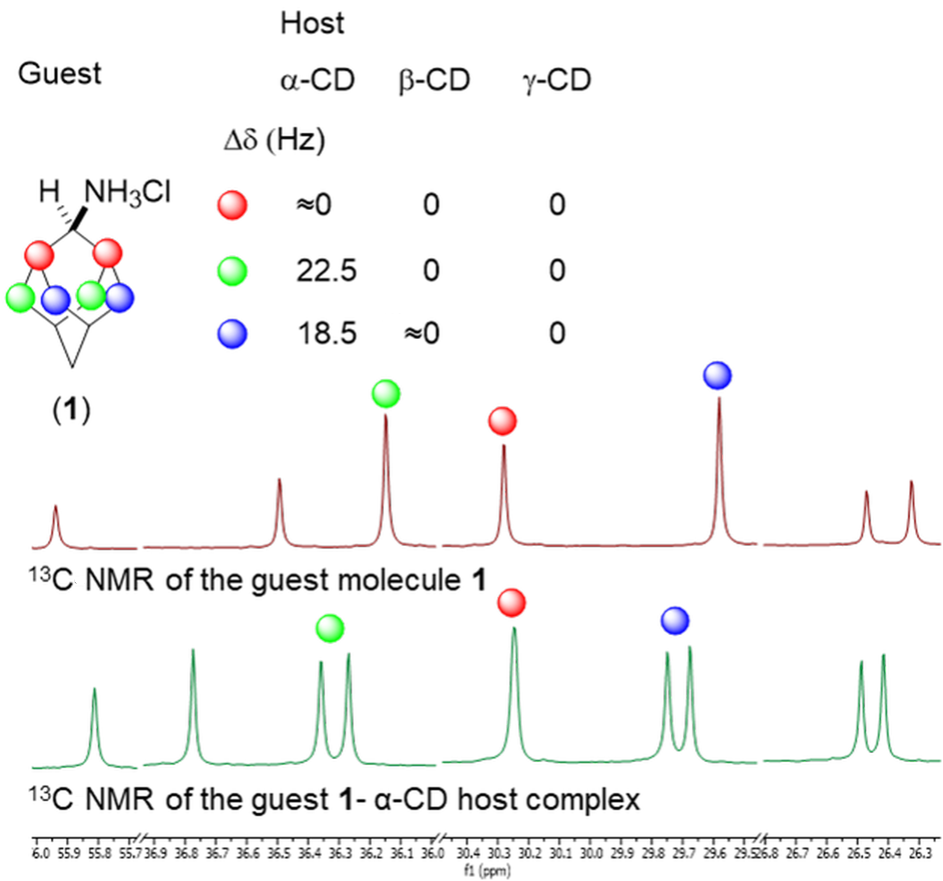
Model system for the elucidation of the solution state conformations of the host–guest complexes. ^13^C NMR splits of prochiral carbons of the guest molecule **1** (adamantan-2-amine hydrochloride) in the anisotropic cavity of the host α-CD in D_2_O solution. Data were acquired at ^13^C Larmor frequency of 150 MHz.

In this work, we reveal the conformation (spatial arrangement) of the host–guest complex in solution spectroscopically by measuring the ^13^C NMR spectra of a suitable guest molecule. We decided to take *C_s_* symmetric guest molecules, CD as a host and measure the ^13^C NMR spectra of these complexes. We expect that the anisotropy of the chiral cavity is expressed by differences in the magnetic shielding of prochiral atoms, resulting in signal splitting of the prochiral carbons of the guest molecule in ^13^C NMR spectra. Adamantan-2-amine hydrochloride was used as a model guest molecule containing three sets of prochiral carbons (Figure 1).

The degree of the signal split correlates with the distance of prochiral atoms (carbons) to the host cavity and thus gives information about the conformation of the guest molecule in the host cavity. First, we measured simple 1D ^13^C NMR spectra and indeed observed the split of the peaks of all prochiral carbon signals of the guest upon complexation with α-CD. The biggest split, 22.5 Hz, was observed for prochiral carbons 8 and 10, depicted in Figure 1 in green color, followed by a split of carbons 3 and 9 (in blue color) and the smallest difference in the magnetic field shielding was detected for carbons 1 and 3 (in red color).

**Figure 2:**
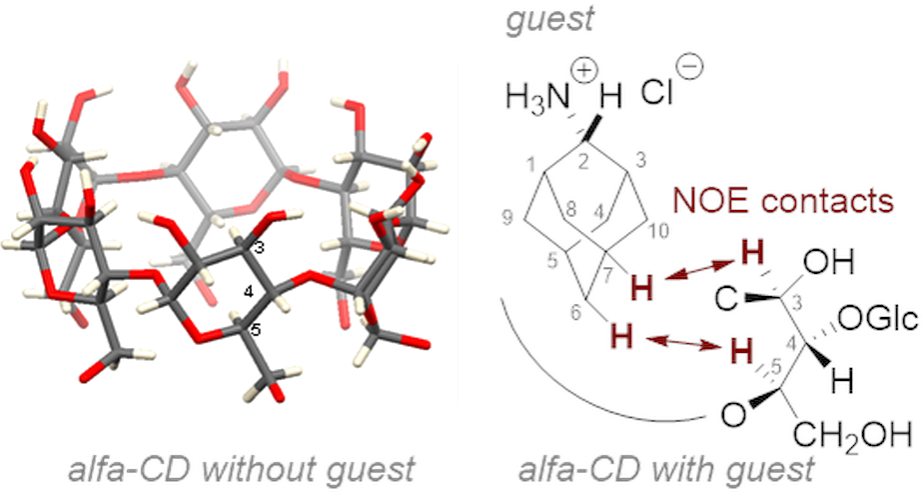
2D ROESY NMR measurements to estimate the depth of the guest molecule **1** (adamantan-2-amine hydrochloride) in the α-CD cavity.

The same 1D ^13^C NMR spectra were measured for guest molecule **1** in host cavities of β-CD and γ-CD, showing almost no split of prochiral carbon peaks and suggesting a higher degree of conformational flexibility of the host–guest complexes than for the complex of **1** with α-CD. Interatomic distances within the host–guest complex were measured using rotating-frame nuclear Overhauser effect spectroscopy (ROESY) measurements. For the complex of **1** with α-CD, the cross-peak in the 2D ROESY spectrum between proton 5 of the glucose moiety of the host α-CD and the protons 6 of molecule **1** reveals deep penetration of the guest into the cavity of α-cyclodextrin (Figure 2). All protons of **1** show ROESY cross-peaks with proton 3 of the glucose unit (see page S73 in Supporting Information File 1).

**Figure 3:**
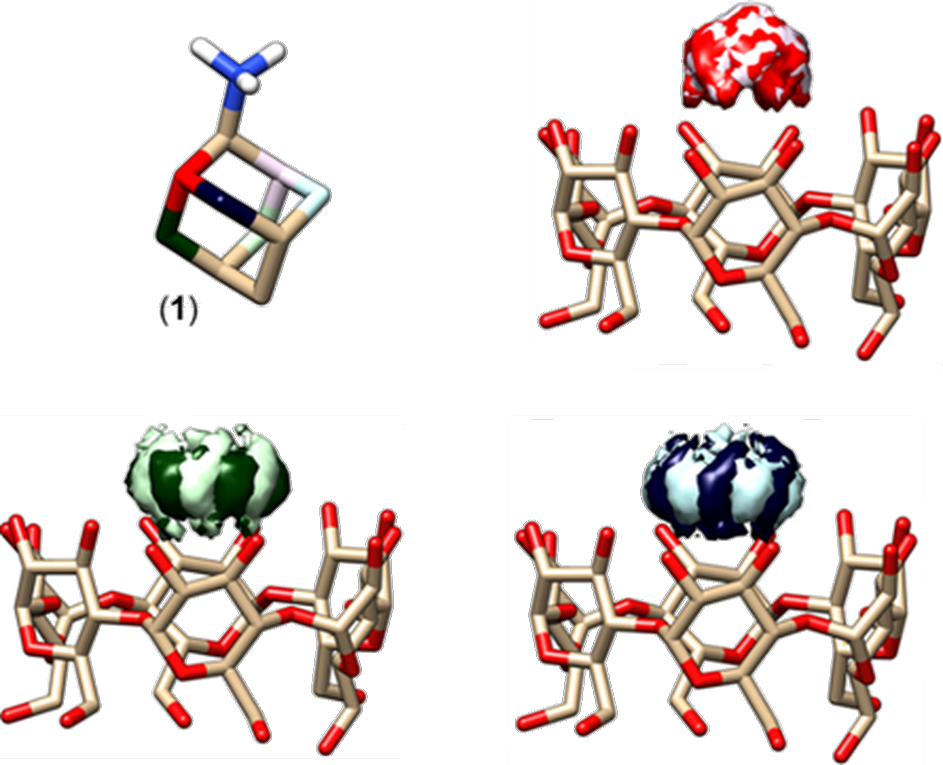
Molecular model of the host (α-CD–guest **1** (adamantan-2-amine hydrochloride)) complex. The 3D densities show the spatial distribution of prochiral atoms within MD simulations.

For complexes of ligands with α-CD, we always performed a series of ten classical molecular dynamics (MD) simulations [16] (each lasting 100 ns, Figure 3). Then, we superimposed α-CD structures from different snapshots of each MD run. Further, the 3D densities, showing the spatial distribution of prochiral atoms of ligands (that rotate and wobble towards α-CD), were calculated. If two atoms have the same spatial density, then the splitting of their signals in ^13^C NMR spectra cannot occur. On the contrary, if these densities, which belong to a pair of prochiral atoms, are well separated, then NMR splitting can be expected due to the anisotropic environment of the chiral α-CD cavity.

The NMR splitting is generally larger if the prochiral guest atoms are located closer to the cavity of the α-CD. In other words, the splitting is larger, if the radius of a density is bigger or the density runs deeper into the α-CD cavity. In Figure 3, the green and blue densities with well-separated light and dark clouds (belonging to different prochiral carbons from a pair) have larger radii and run deeper into the α-CD cavity than the red density with mixed light and dark clouds. Accordingly, our NMR experiment only showed splitting for the green and blue atoms (see Figure 1).

**Figure 4:**
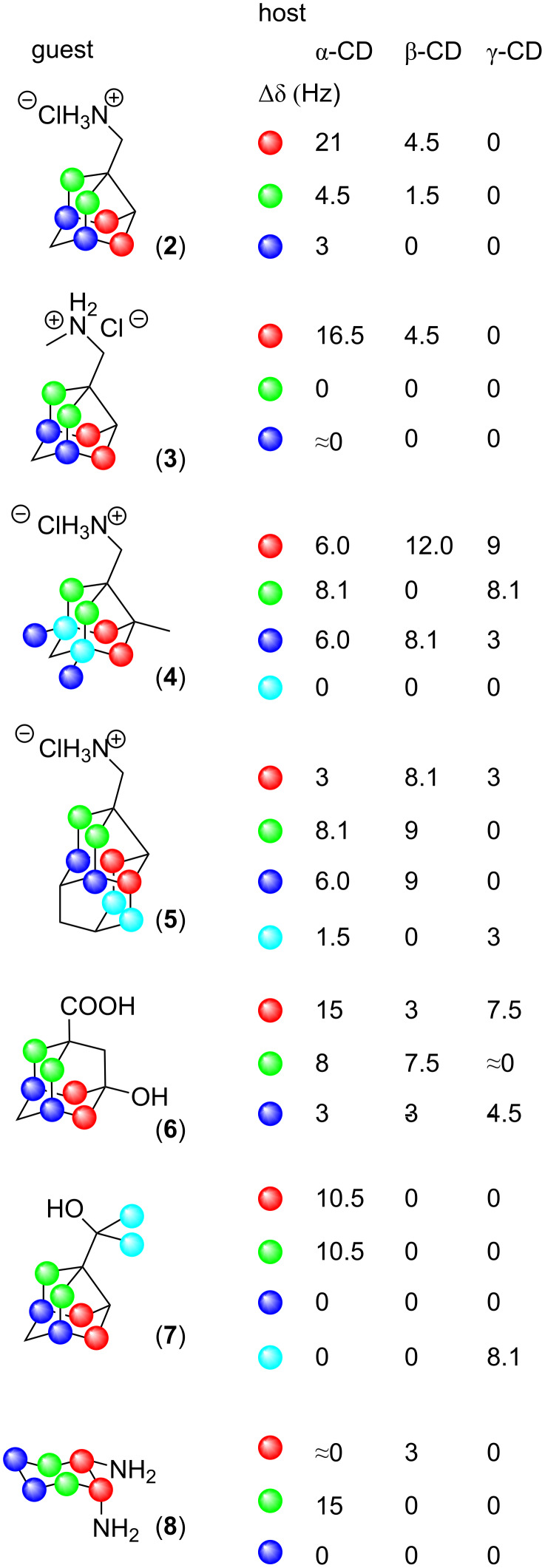
Studied host–guest complexes and splitting of guests’ prochiral carbons in their ^13^C NMR spectra.

Having established the method, we chose various cyclic compounds (noradamantane, adamantane, cyclohexane derivatives) with desired symmetry and measured NMR spectra of these guest molecules (**2**–**8**) in α-cyclodextrin and cyclodextrins with a larger cavity (β-CD and γ-CD, Figure 4).

**Figure 5:**
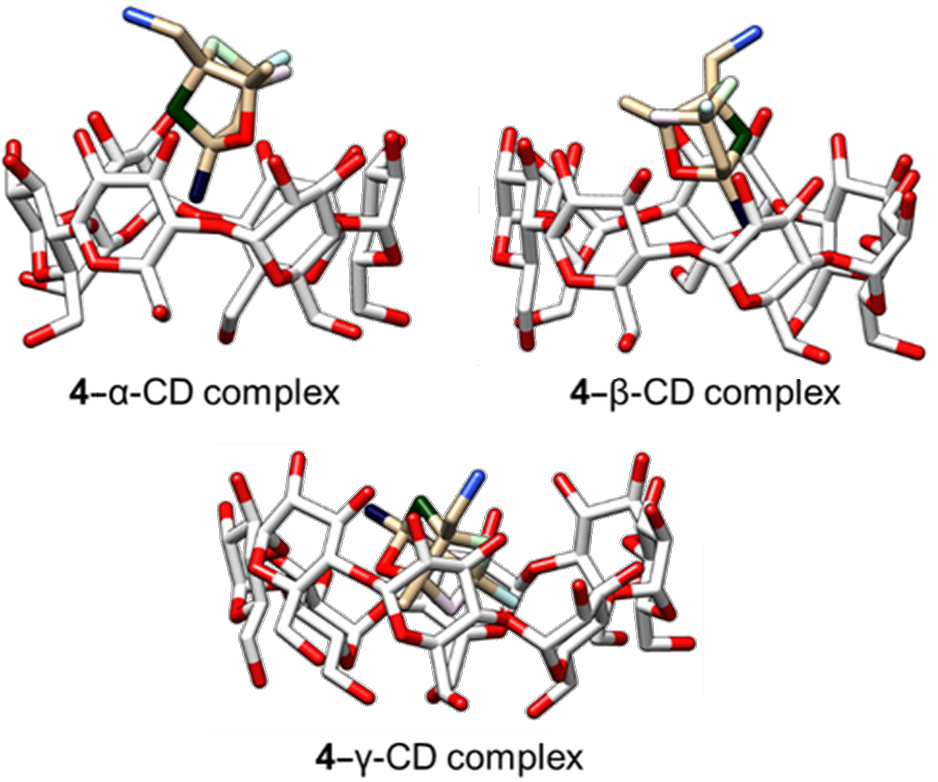
Molecular modelling of the host–guest complexes of compound **4** with α-CD, β-CD and γ-CD.

In all cases, the ^1^H NMR spectra indicated the interaction of the guest molecule with the host. In cases where the split of prochiral carbons was observed, ^13^C NMR spectra showed which pair of prochiral carbons in the guest molecule is close to the wall of the host’s chiral cavity, creating an anisotropic environment. For compound **4,** we were thus able to select representative conformations of the guest molecule in all types of cyclodextrins (Figure 5) using the spatial densities gained from classical MD simulations.

**Figure 6:**
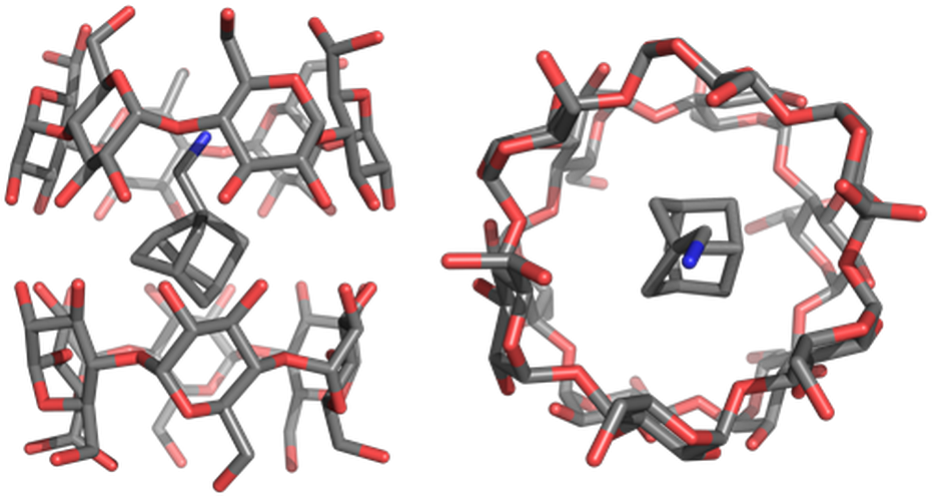
X-ray analysis of the α-CD supramolecular capsule with noradamantane-3-methyleneamine (**2**) as guest molecule (side and top view).

Compounds **1**–**8** (as hydrochloride salts or free bases), were attempted to co-crystallize with α-, β- and γ-cyclodextrins. Compounds **1**, **6** and **8** are commercial, compounds **2**–**5** [21] and **7** [22] were prepared according to published procedures. Compound **2** (as a free amine) crystallized in the supramolecular capsule of two α-cyclodextrins, and the mono-crystal was subjected to X-ray analysis. This experiment was performed to compare the solid-state structure of the guest **2**–α-CD host complex with its proposed solution state conformations (Figure 6).

## Conclusion

We have demonstrated that simple ^13^C NMR analyses of properly chosen *C_s_* symmetric compounds varying in size can be used to estimate the host–guest spatial arrangement in solution and extrapolated for compounds with similar size and bonding nature.

## Supporting Information

File 1 General information, NMR spectra, NMR study, computational study, crystallographic data collection and refinement details.

## Data Availability

All data that supports the findings of this study is available in the published article and/or the supporting information to this article.
