# Supplementary material for: Spatial arrangements of cyclodextrin host–guest complexes in solution studied by 13C NMR and molecular modelling
Source: Beilstein J Org Chem. 2024 Feb 20;20:331–5. doi: 10.3762/bjoc.20.33 (PMC10896224; doi:10.3762/bjoc.20.33)
Supplement: File 1 — General information, NMR spectra, NMR study, computational study, crystallographic data collection and refinement details. [file Beilstein_J_Org_Chem-20-331-s001.pdf]

BEILSTEIN JOURNAL OF ORGANIC CHEMISTRY

Supporting Information

for

Spatial arrangements of cyclodextrin host–guest complexes in solution studied by ^{13}C NMR and molecular modelling

Konstantin Lebedinskiy, Ivan Barvík, Zdeněk Tošner, Ivana Císařová, Jindřich Jindřich and Radim Hrdina

Beilstein J. Org. Chem. **2024,** *20,* 331–335. doi:10.3762/bjoc.20.33

General information, NMR spectra, NMR study, computational study, crystallographic data collection and refinement details

License and Terms: This is a supporting information file under the terms of the Creative Commons Attribution License (https://creativecommons.org/licenses/by/4.0). Please note that the reuse, redistribution and reproduction in particular requires that the author(s) and source are credited and that individual graphics may be subject to special legal provisions.
The license is subject to the *Beilstein Journal of Organic Chemistry* terms and conditions: (https://www.beilstein-journals.org/bjoc/terms)

Content

1. General information S2

2. NMR spectra S3

3. NMR study S72

4. Computational study S73

5. Crystallographic data collection and refinement details S84

1. General information

α-, β- and γ-cyclodextrins (CDs) were purchased from Wacker company (Germany), and before use, they were recrystallized from a hot water solution (β-CD) or water/methanol solution (α-CD, γ-CD). Other chemicals (including compounds **1**, **6**, **8**) were supplied by common commercial sources (Sigma-Aldrich, FluoroChem, etc). Compounds **2**, **3**, **4**, **5** and **7** were synthesized according to the described procedures cited in the article (Scheme 1). NMR spectra were measured on a Bruker Avance III HD 600 spectrometer at 25 °C. Signals of tetramethylsilane (for ^{1}H NMR) and chloroform (for ^{13}C NMR) served as internal standards. All samples for NMR analysis were measured in deuterated water. To obtain reasonable ^{13}C NMR spectra, solutions of a guest (7–15 mM) were prepared with no buffer. Each guest solution was partitioned to prepare complex solutions with a corresponding cyclodextrin whose concentration twice exceeded guest concentration for α- and γ-cyclodextrins and was equimolar solutions for β-cyclodextrin. ^{13}C and ^{13}C-DEPT spectra (2000 scans) of these samples were measured together with blank solutions with a corresponding guest. To measure ^{1}H and 2D NMR (COSY, HSQC, HMBS, ROESY, NOESY) spectra, less concentrated solutions ($C_{gues\ t} \approx$ 2–3 mmol; $C_{CD} \approx$ 3–6 mmol) were used.

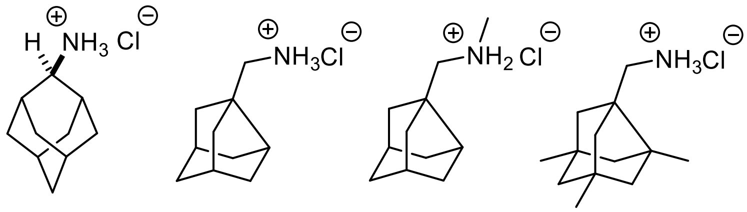

(1) (2) (3) (4)

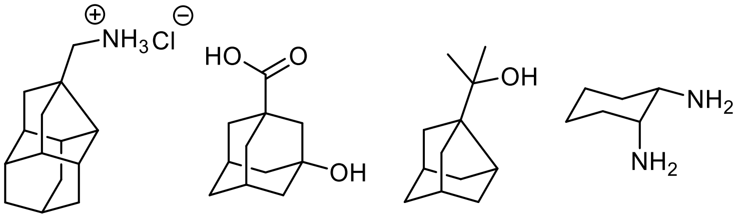

(5) (6) (7) (8)

Scheme 1 Guest compounds used for complexations with cyclodextrins

2. NMR spectra

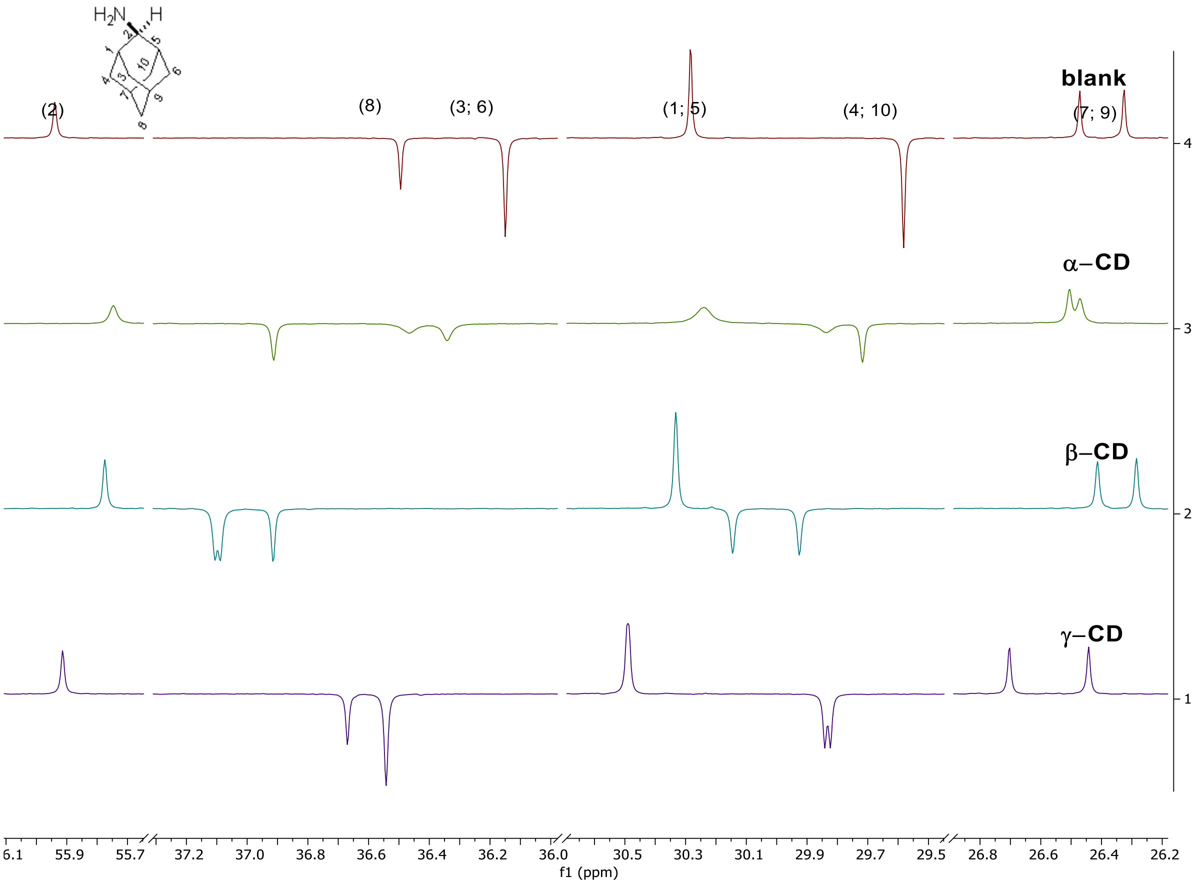

Figure S1 The partial ^{13}C DEPT spectra of **1** (15 mM) water solution in the absence of any host (blank); in the presence of α-CD (30 mM); β-CD (15 mM); γ-CD (30 mM) (no buffer)

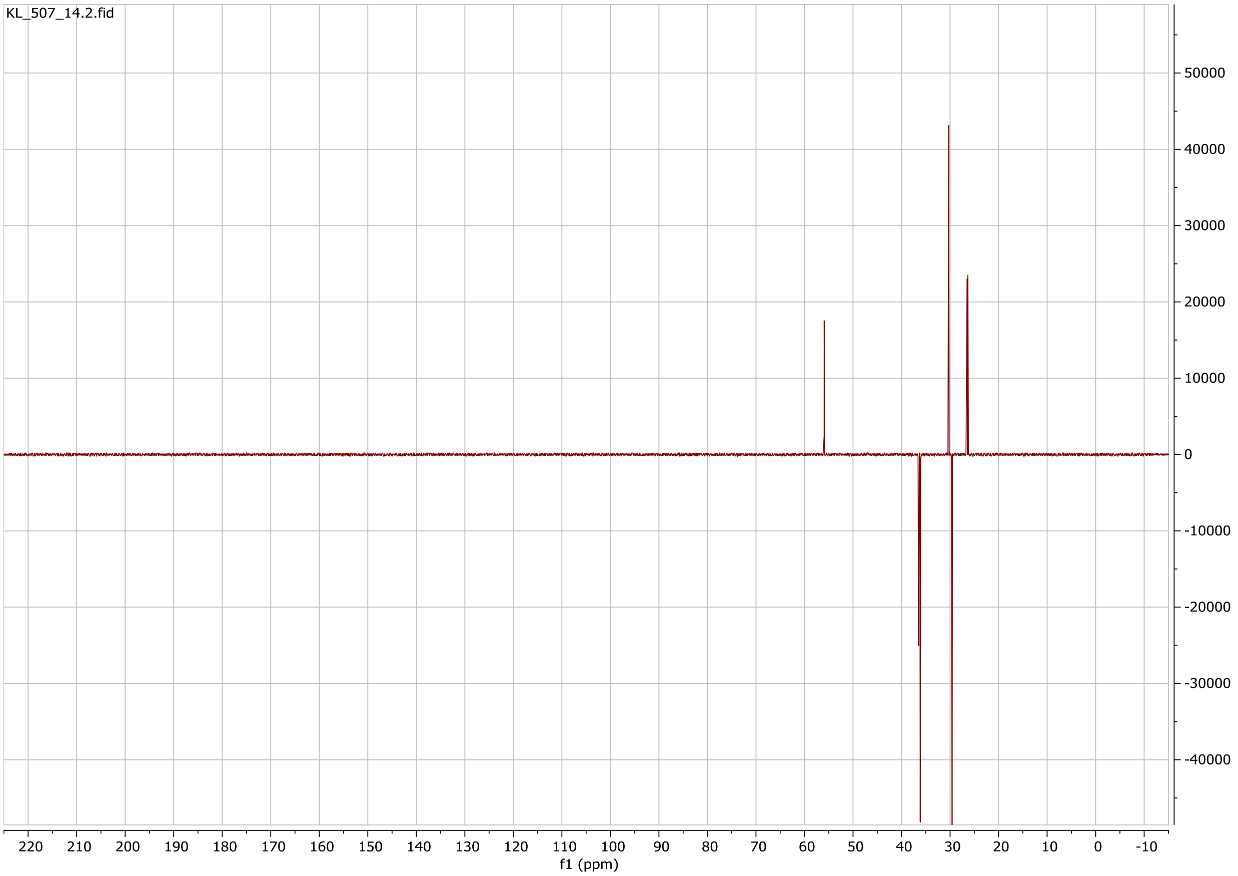

Figure S2 The ^{13}C DEPT spectra of **1** (15 mM) water solution (no buffer)

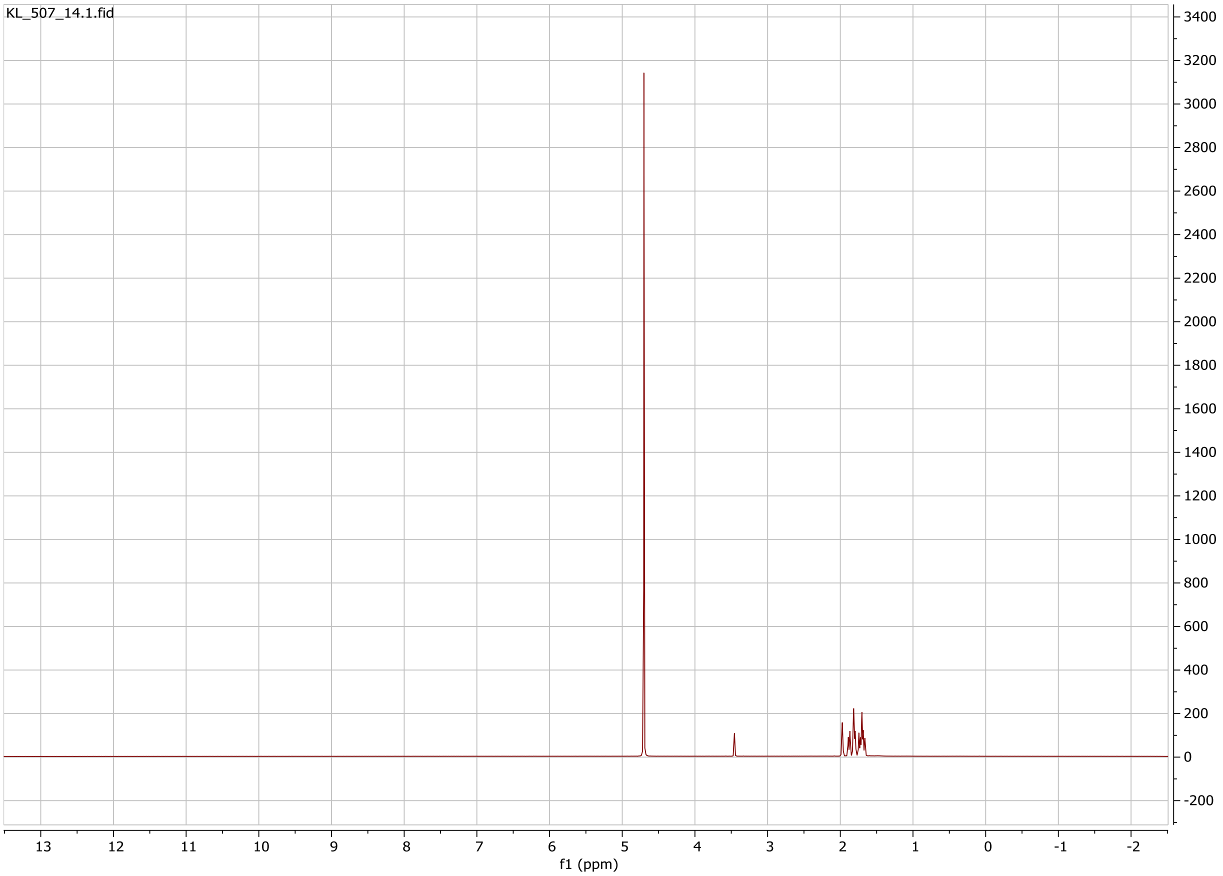

Figure S3 The ^{1}H NMR spectra of **1** (15 mM) water solution (no buffer)

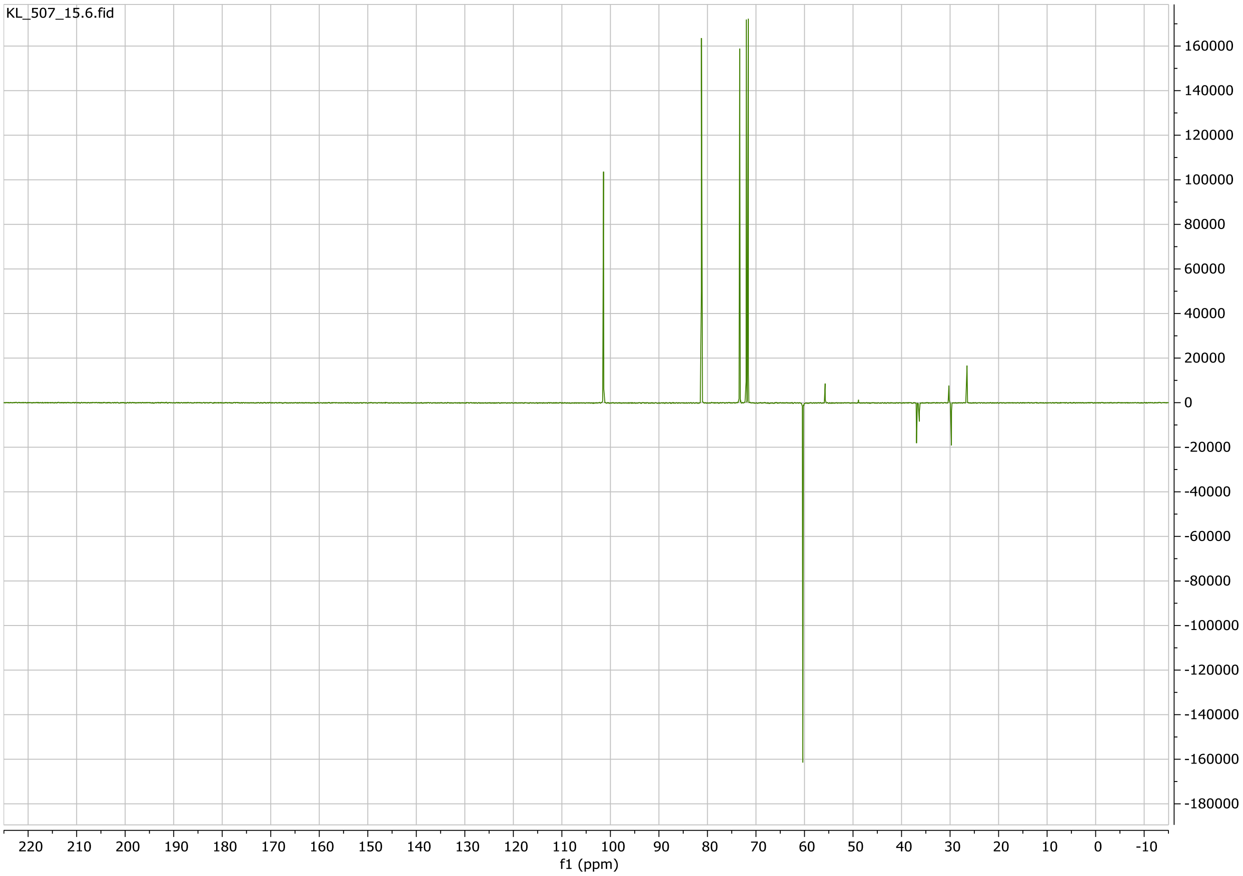

Figure S4 The ^{13}C DEPT spectra of **1** (15 mM) water solution in the presence of α-CD (30 mM); (no buffer)

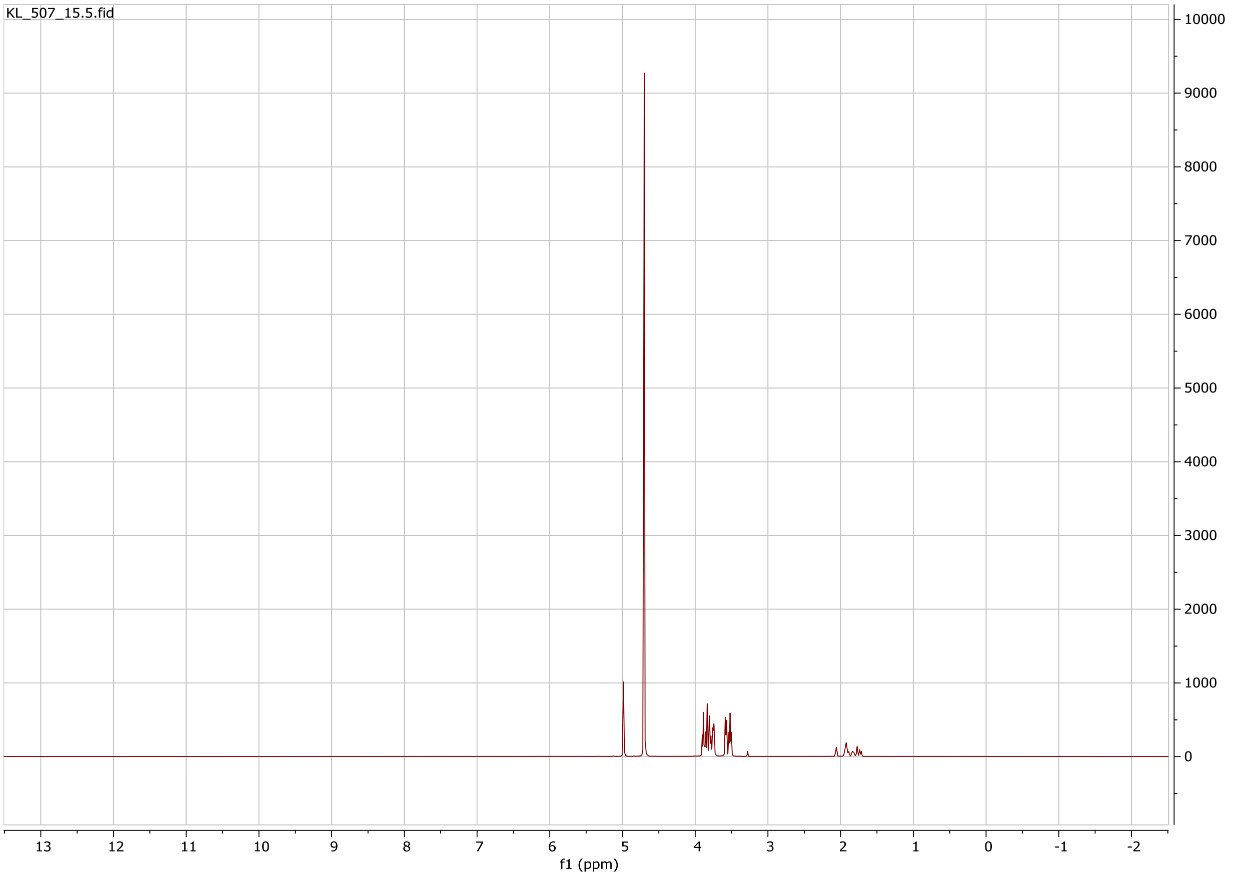

Figure S5 The ^{1}H NMR spectra of **1** (15 mM) water solution in the presence of α-CD (30 mM); (no buffer)

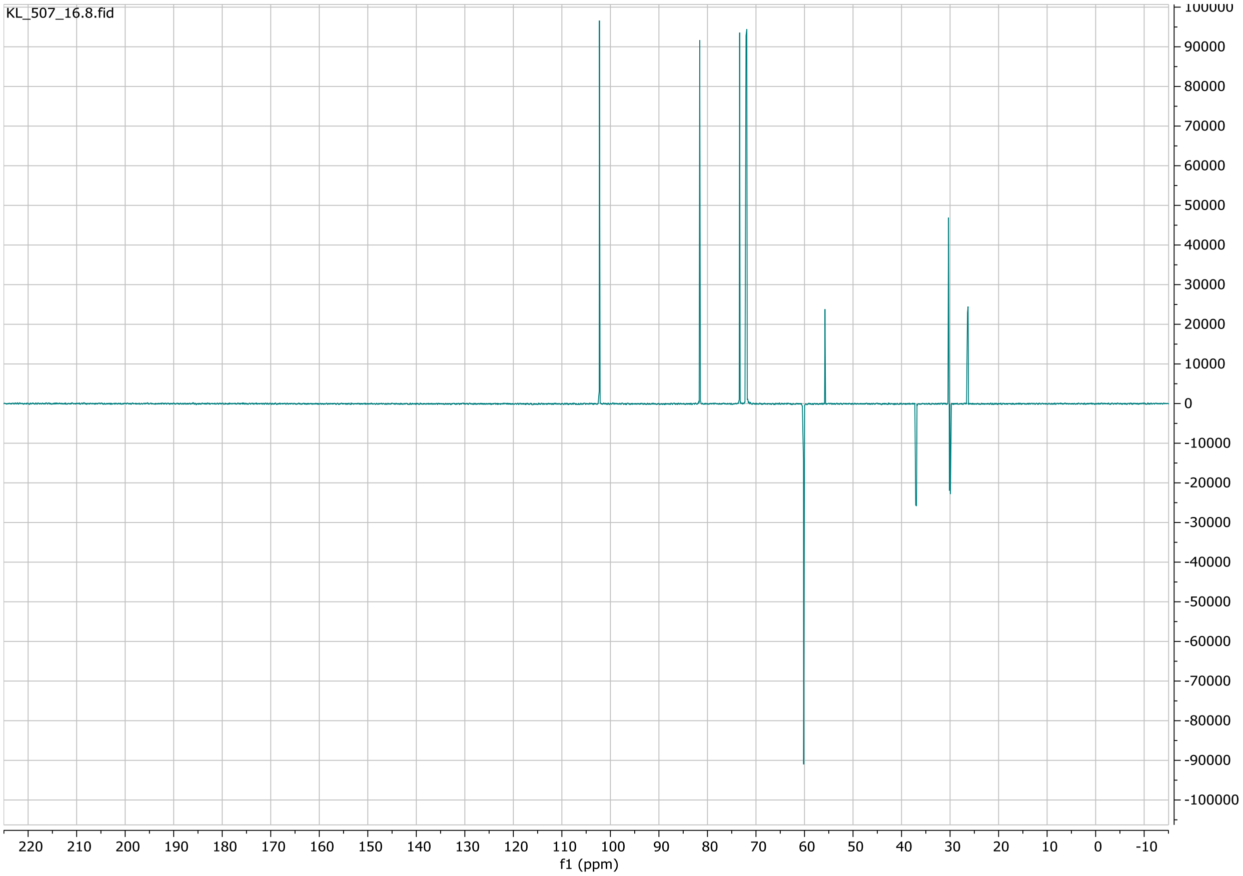

Figure S6 The ^{13}C DEPT spectra of **1** (15 mM) water solution in the presence of β-CD (15 mM) (no buffer)

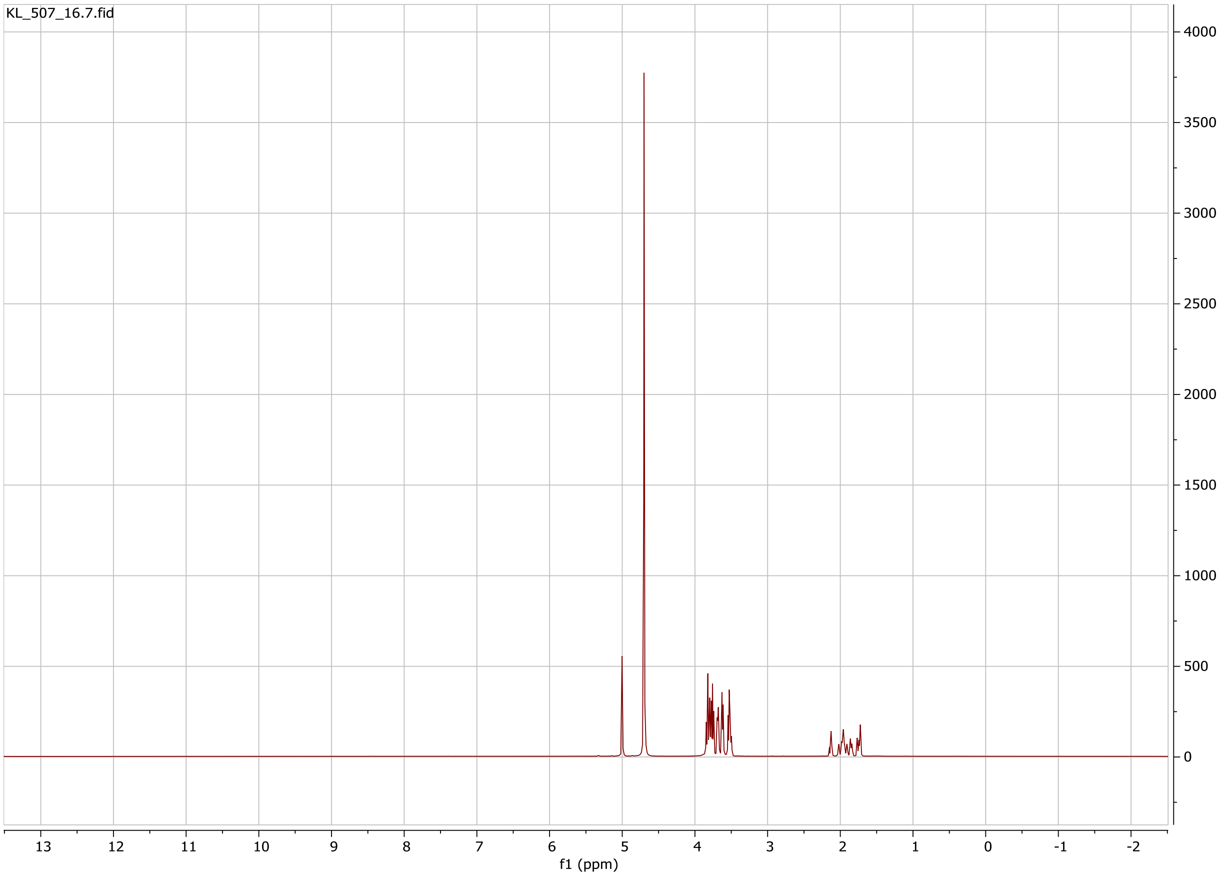

Figure S7 The ^{1}H NMR spectra of **1** (15 mM) water solution in the presence of β-CD (15 mM) (no buffer)

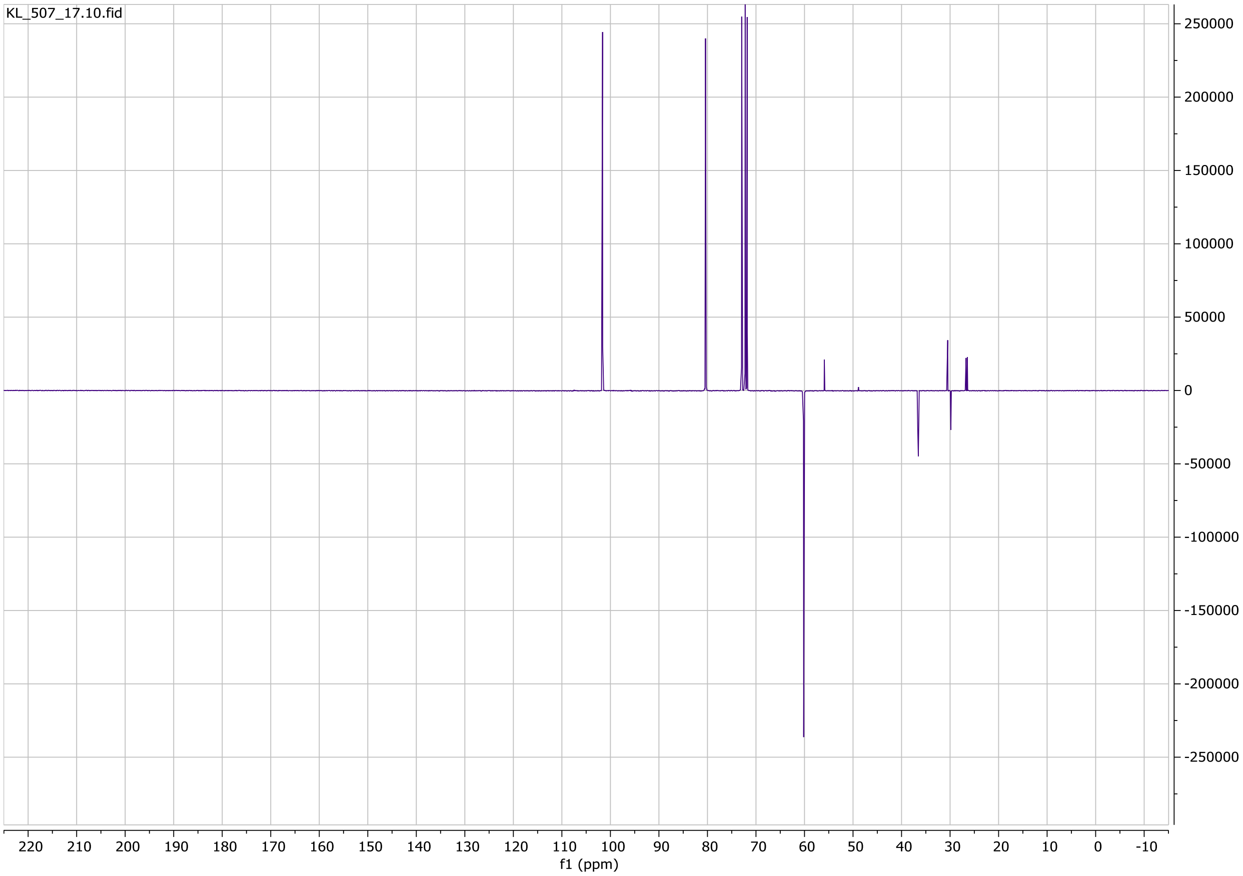

Figure S8 The ^{13}C DEPT spectra of **1** (15 mM) water in the presence of γ-CD (30 mM) (no buffer)

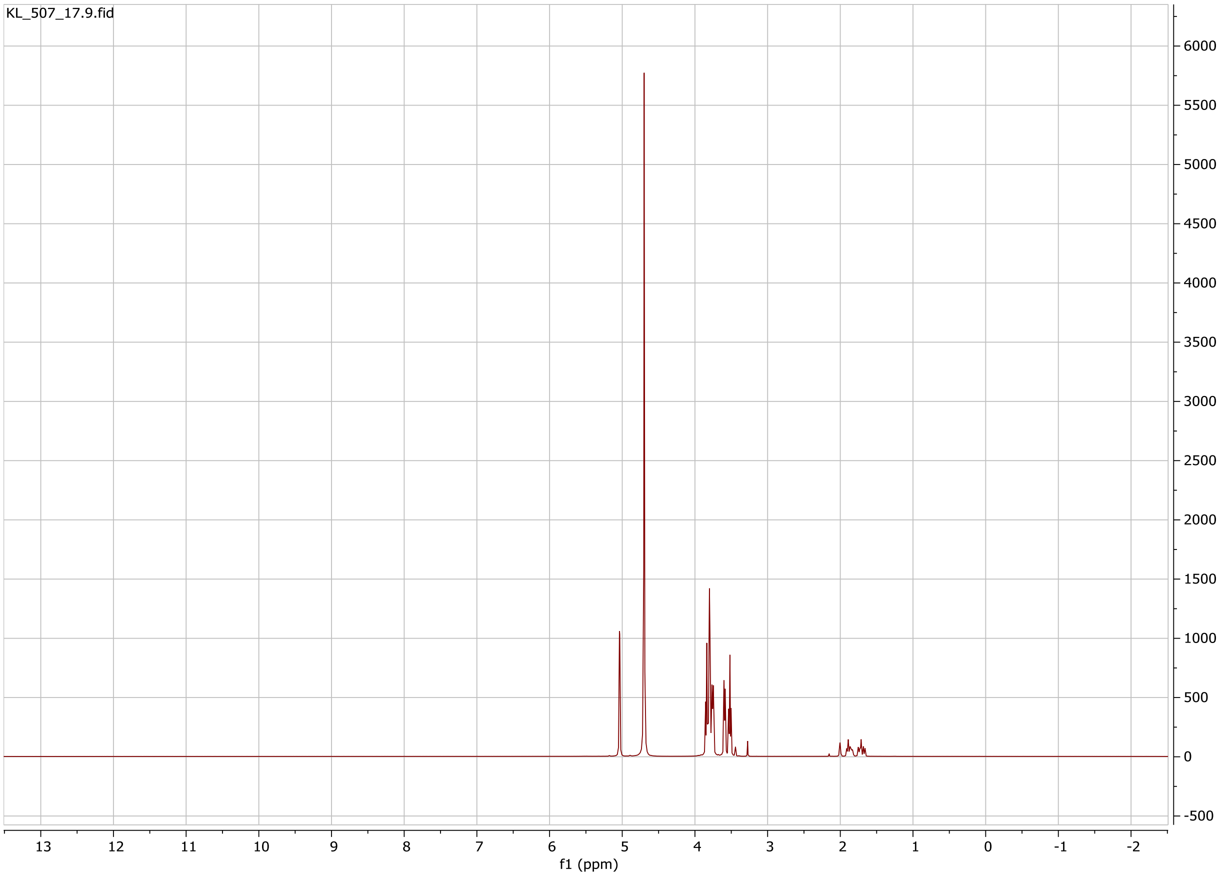

Figure S9 The ^{1}H NMR spectra of **1** (15 mM) water in the presence of γ-CD (30 mM) (no buffer)

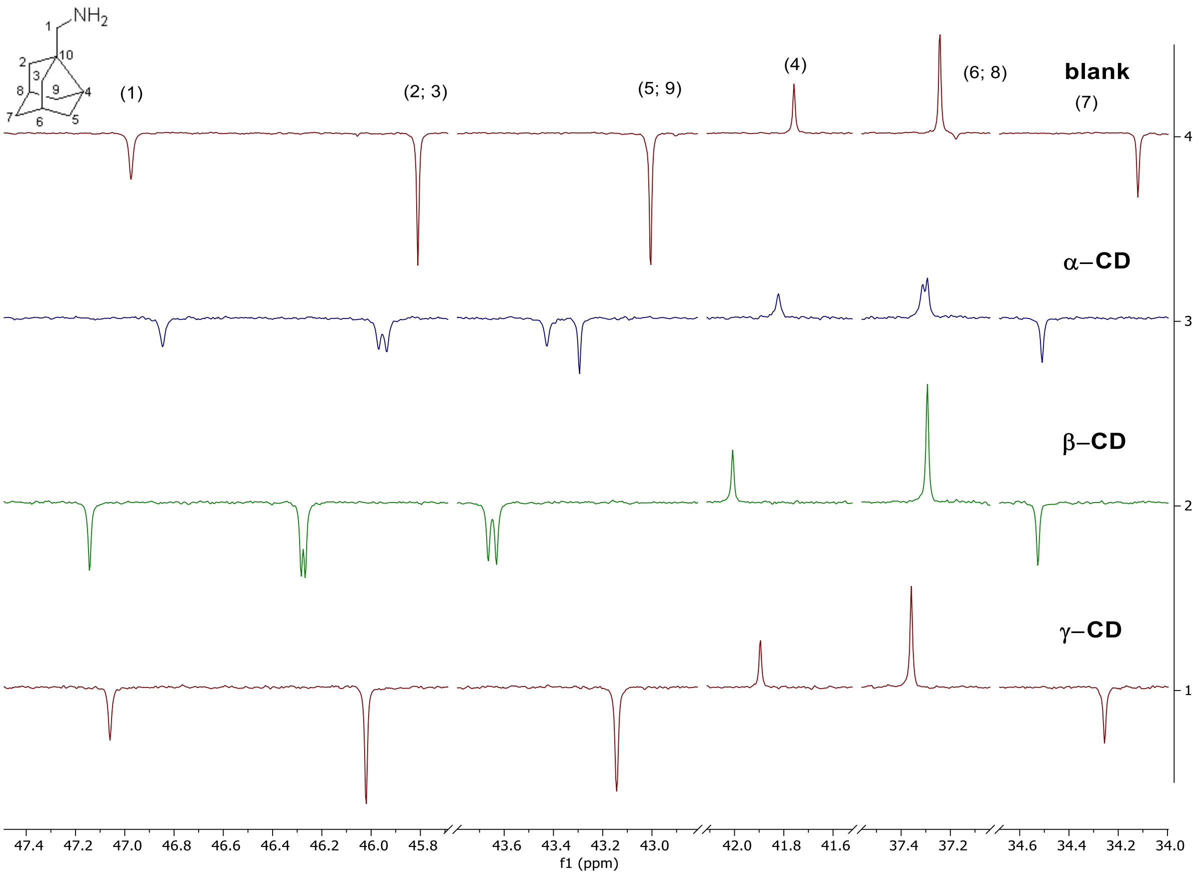

Figure S10 The partial ^{13}C DEPT spectra of **2** (10 mM) water solution in the absence of any host (blank); in the presence of α-CD (20 mM); β-CD (10 mM); γ-CD (20 mM) (no buffer).

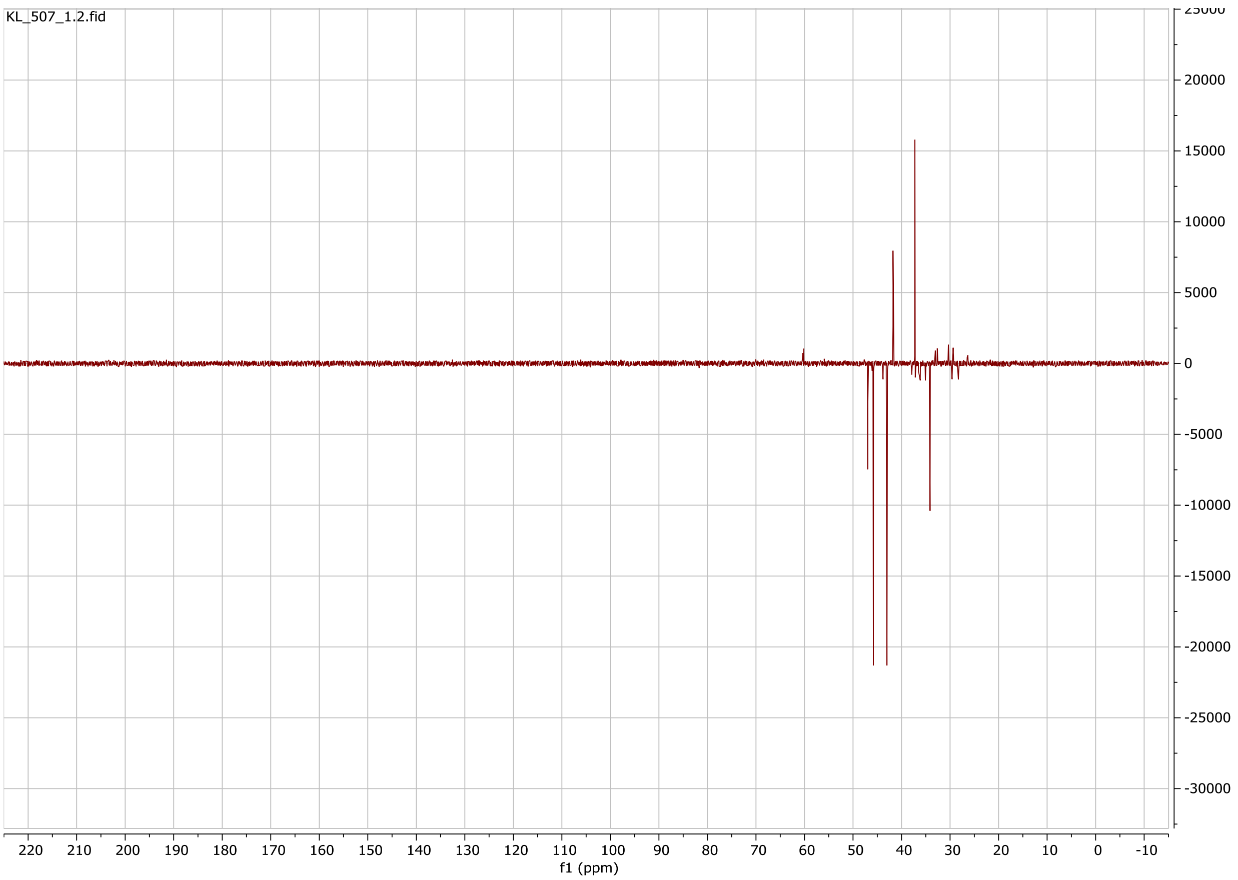

Figure S11 The ^{13}C DEPT spectra of **2** (10 mM) water solution (no buffer).

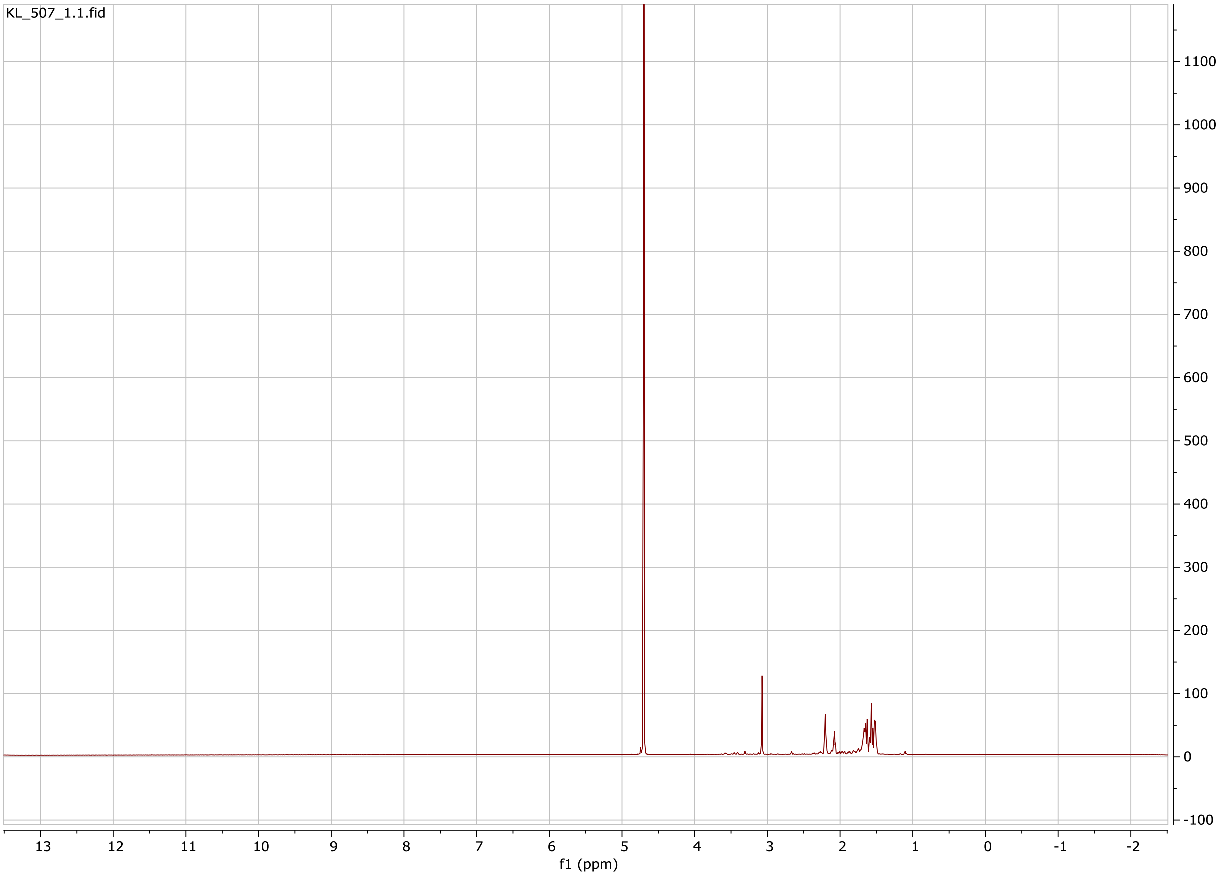

Figure S12 The ^{1}H NMR spectra of **2** (10 mM) water solution (no buffer).

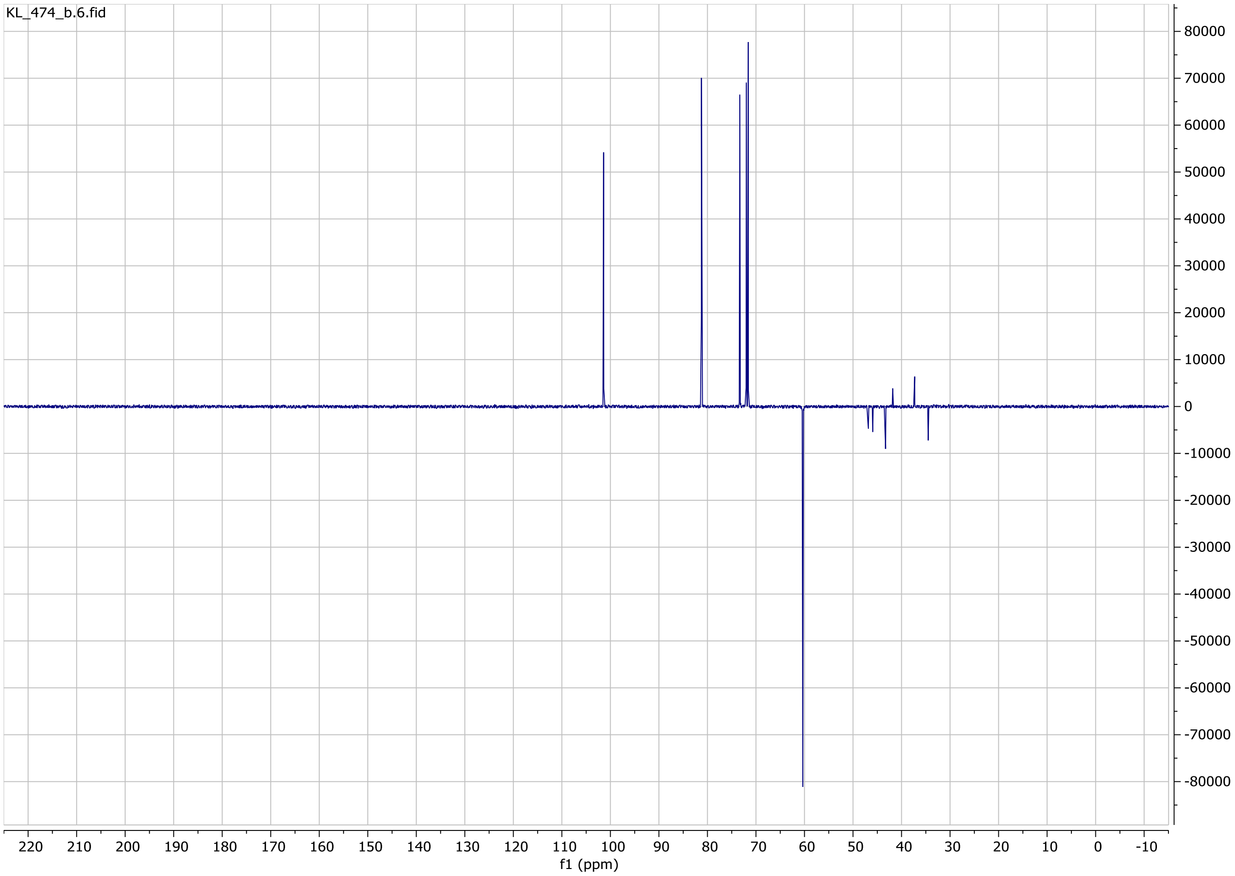

Figure S13 The ^{13}C DEPT spectra of **2** (10 mM) water solution in the presence of α-CD (20 mM) (no buffer).

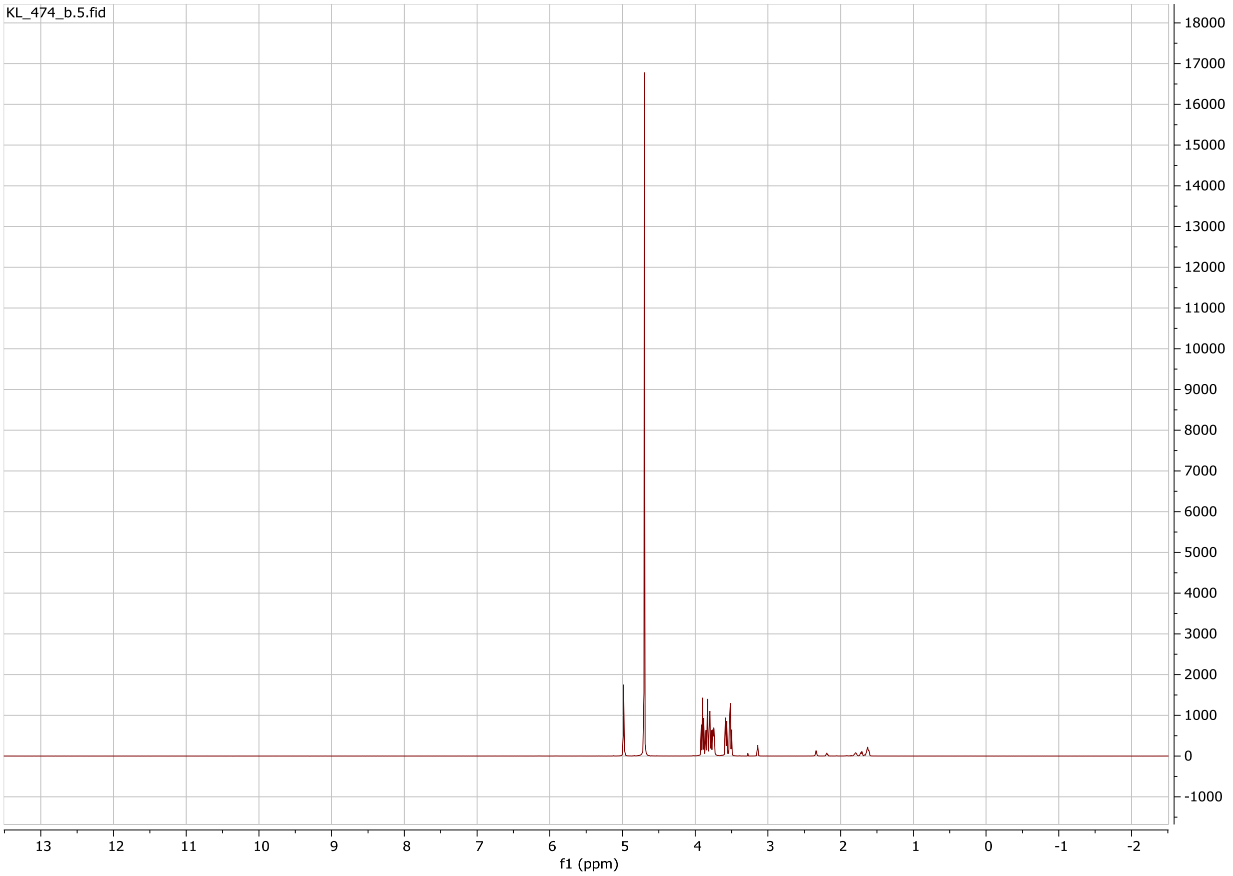

Figure S14 The ^{1}H NMR spectra of **2** (10 mM) water solution in the presence of α-CD (20 mM) (no buffer).

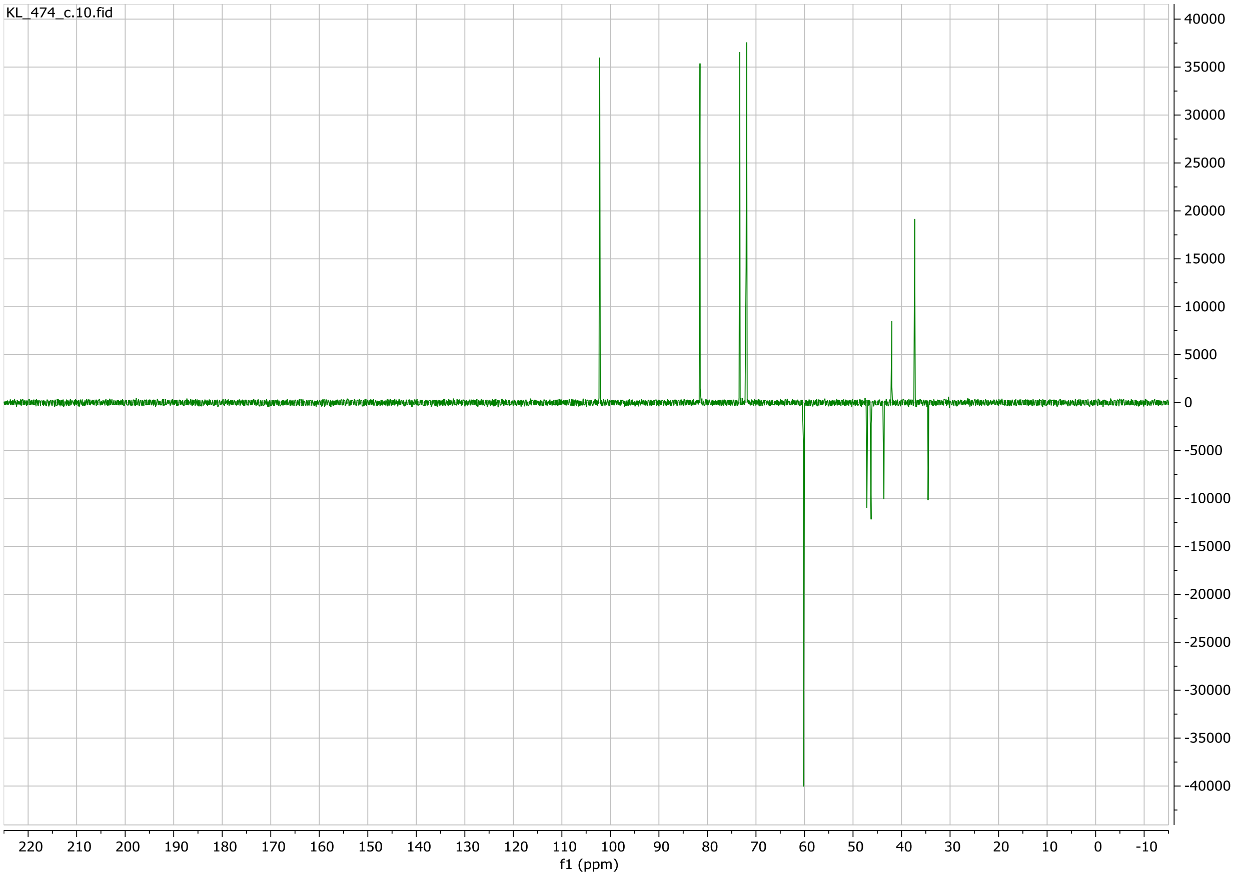

Figure S15 The ^{13}C NMR spectra of **2** (10 mM) water solution in the presence of β-CD (10 mM) (no buffer).

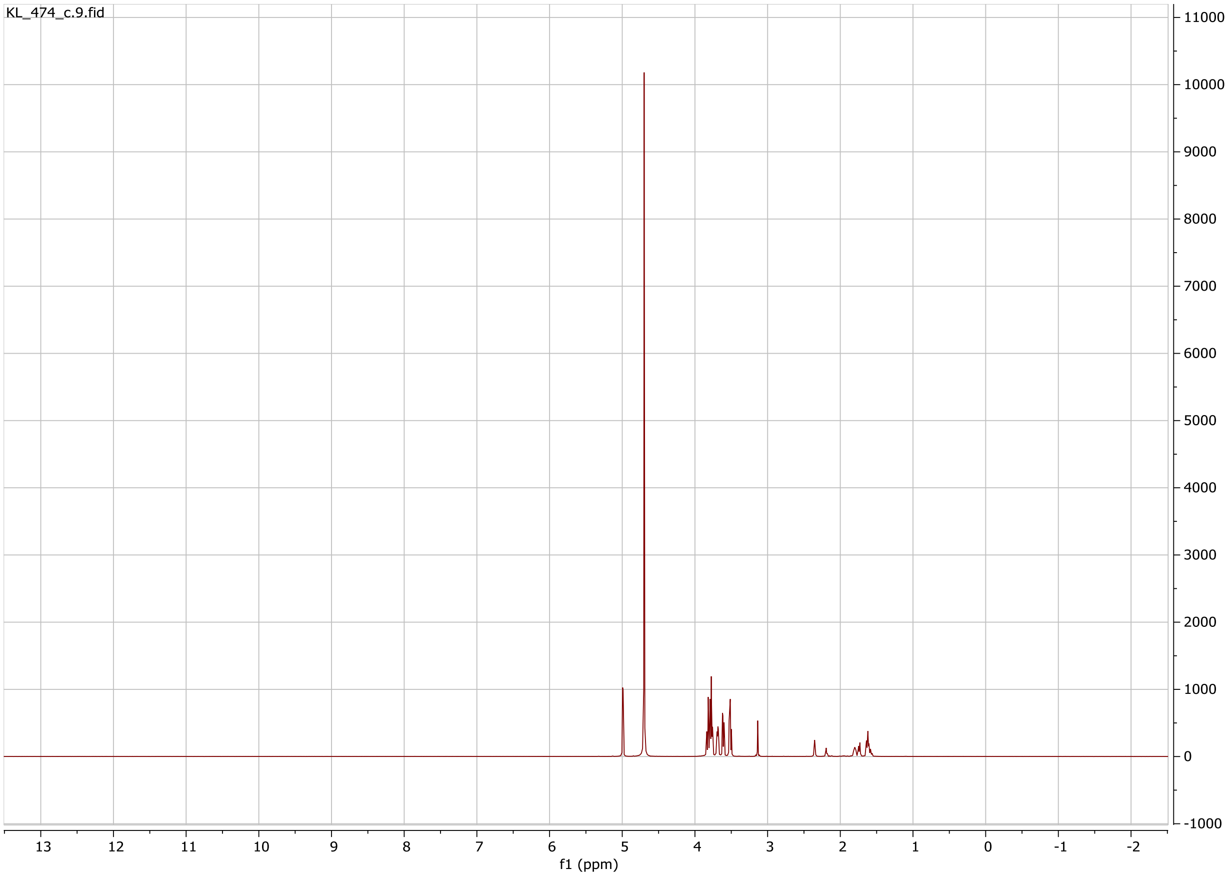

Figure S16 The ^{1}H NMR spectra of **2** (10 mM) water solution in the presence of β-CD (10 mM) (no buffer).

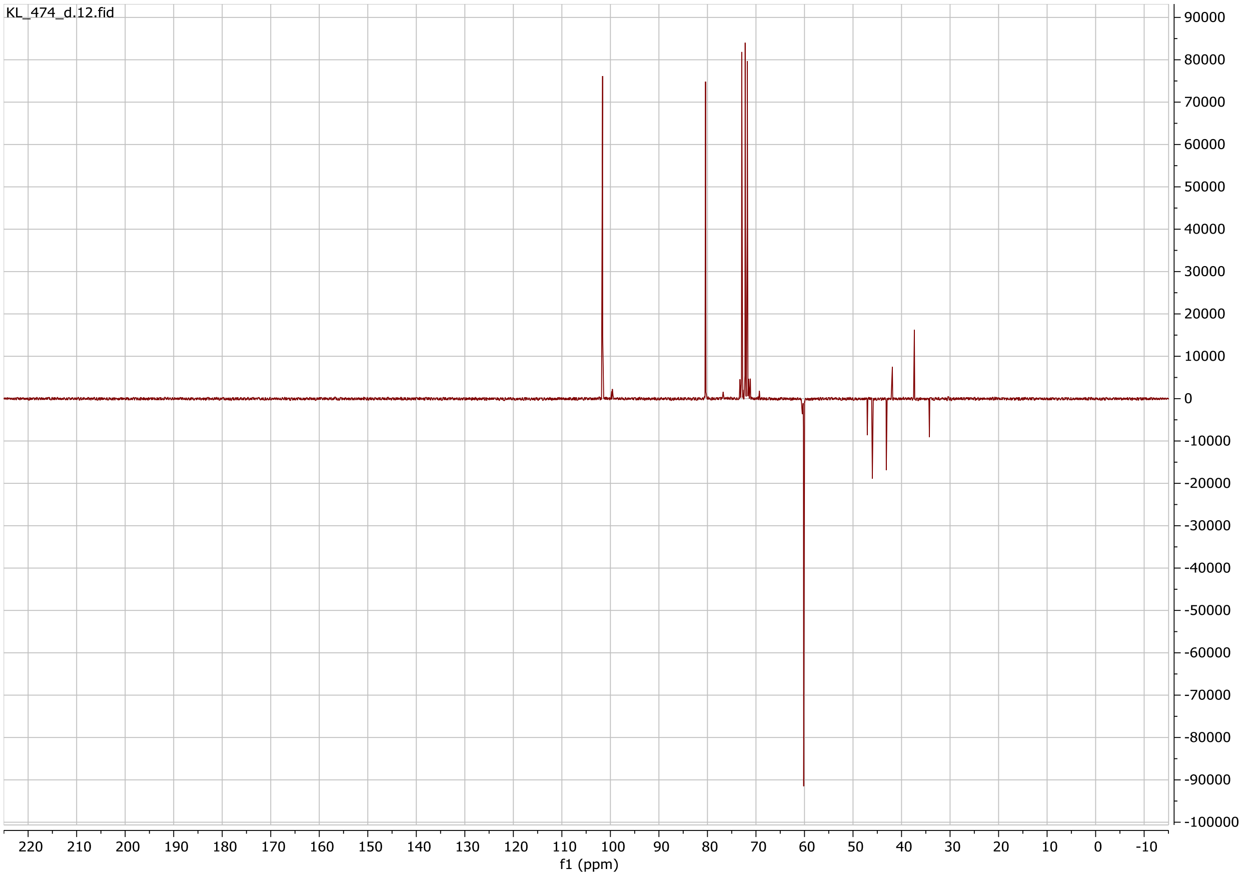

Figure S17 The ^{13}C DEPT spectra of **2** (10 mM) water solution in the presence of γ-CD (20 mM) (no buffer).

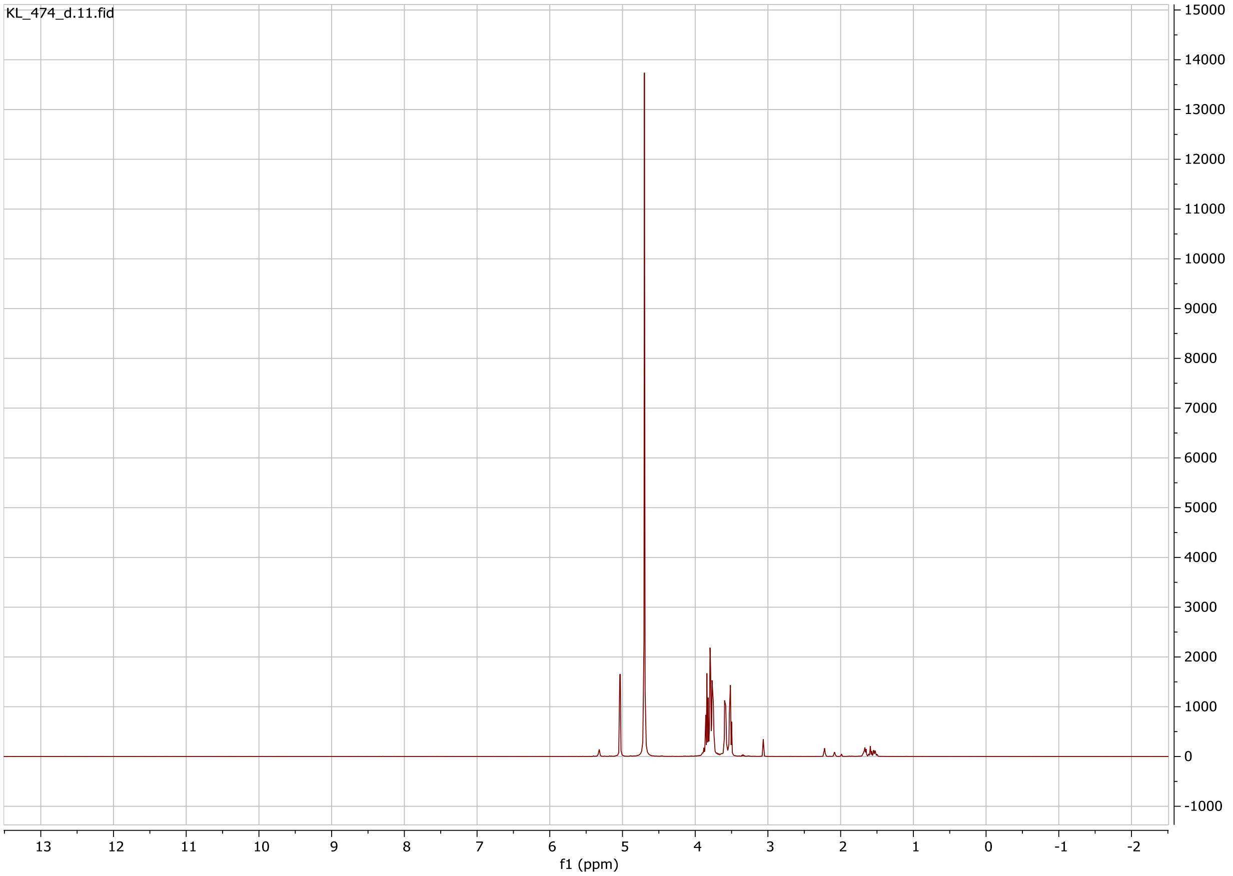

Figure S18 The ^{1}H NMR spectra of **2** (10 mM) water solution in the presence of γ-CD (20 mM) (no buffer).

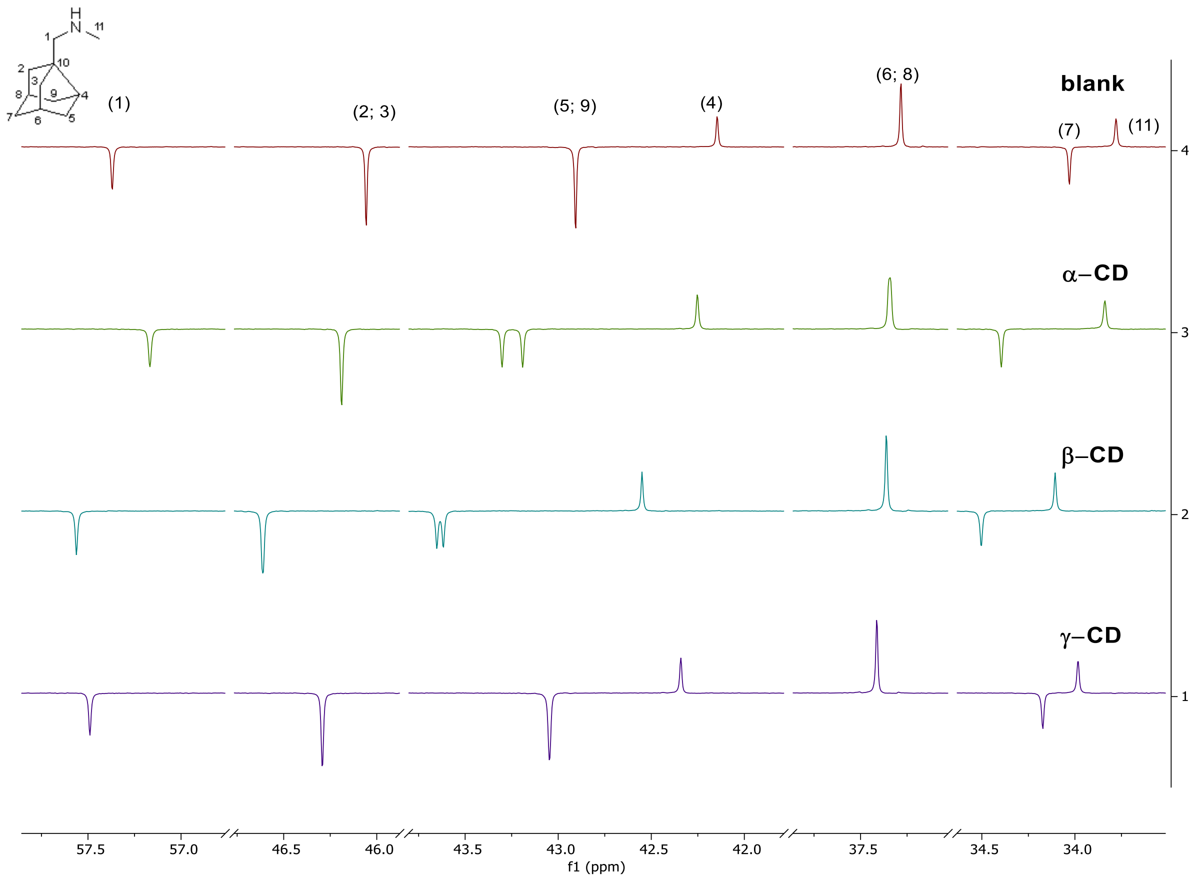

Figure S19 The partial ^{13}C DEPT spectra of **3** (15 mM) water solution in the absence of any host (blank); in the presence of α-CD (30 mM); β-CD (15 mM); γ-CD (30 mM) (no buffer)

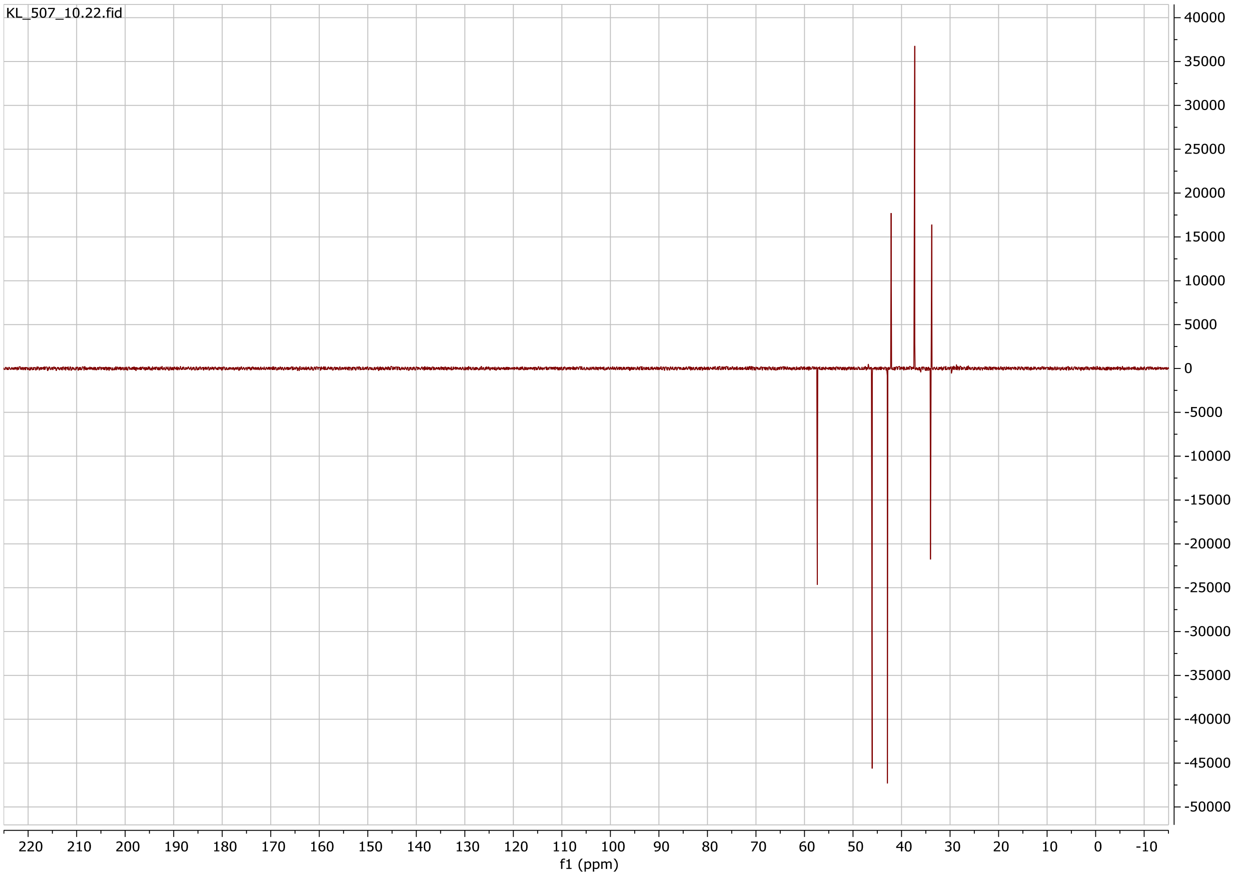

Figure S20 The ^{13}C DEPT spectra of **3** (15 mM) water solution (no buffer)

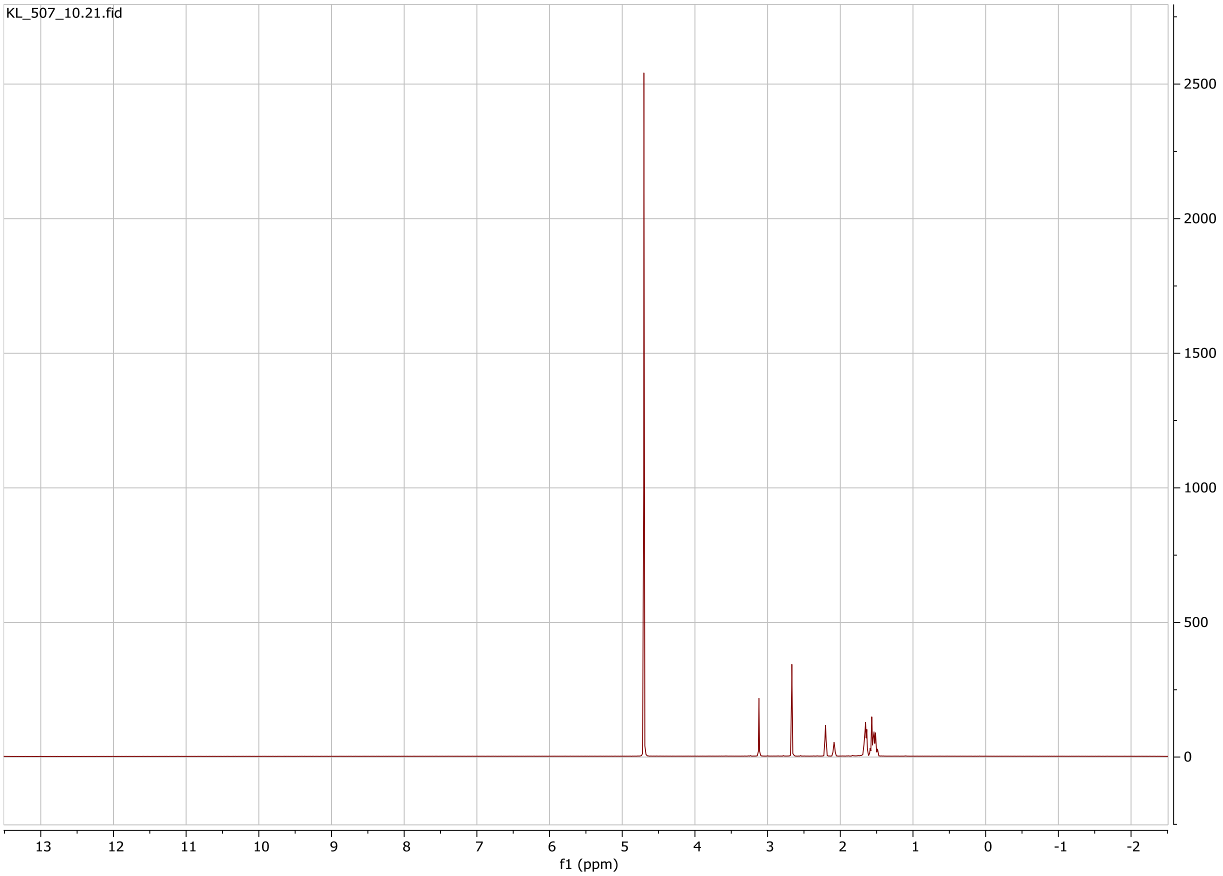

Figure S21 The ^{1}H NMR spectra of **3** (15 mM) water solution (no buffer)

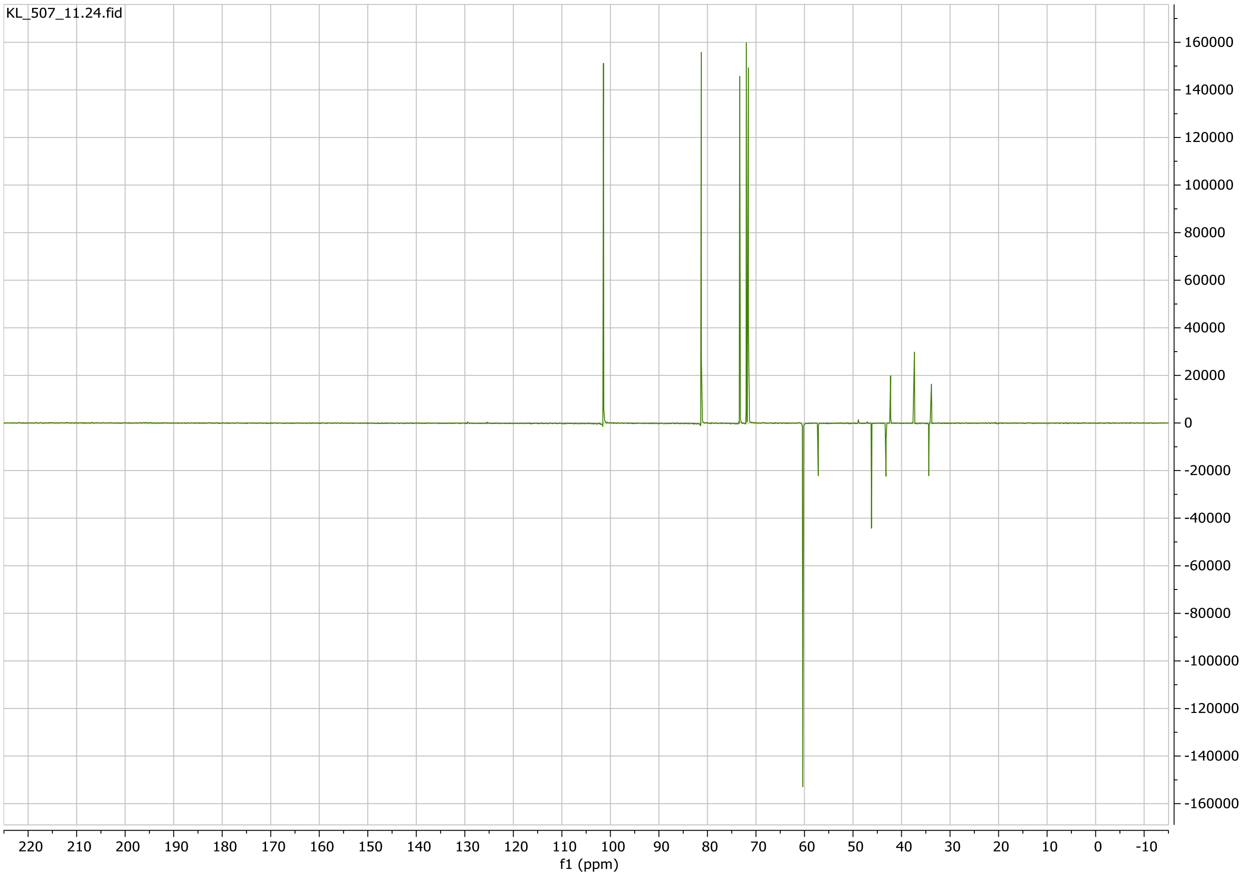

Figure S22 The ^{13}C DEPT spectra of **3** (15 mM) water solution in the presence of α-CD (30 mM) (no buffer)

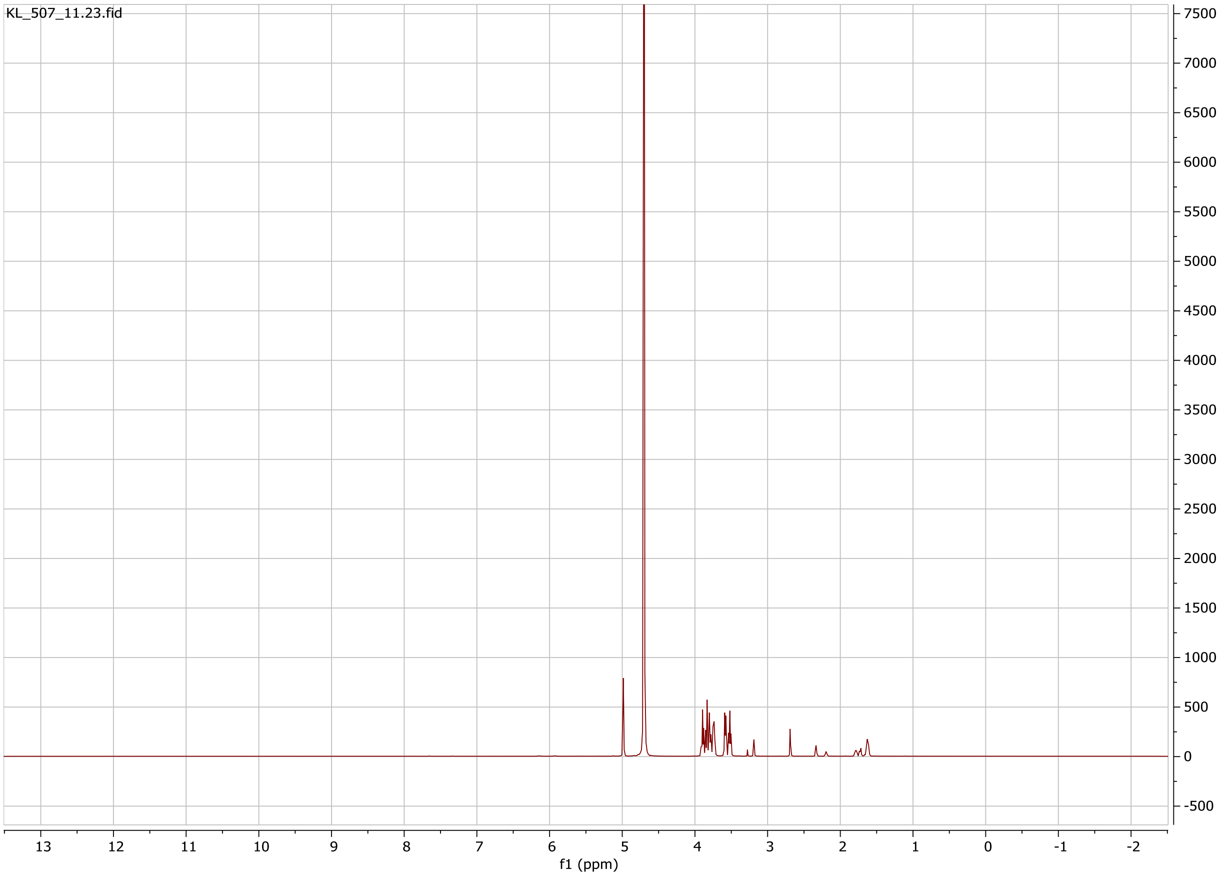

Figure S23 The ^{1}H NMR spectra of **3** (15 mM) water solution in the presence of α-CD (30 mM) (no buffer)

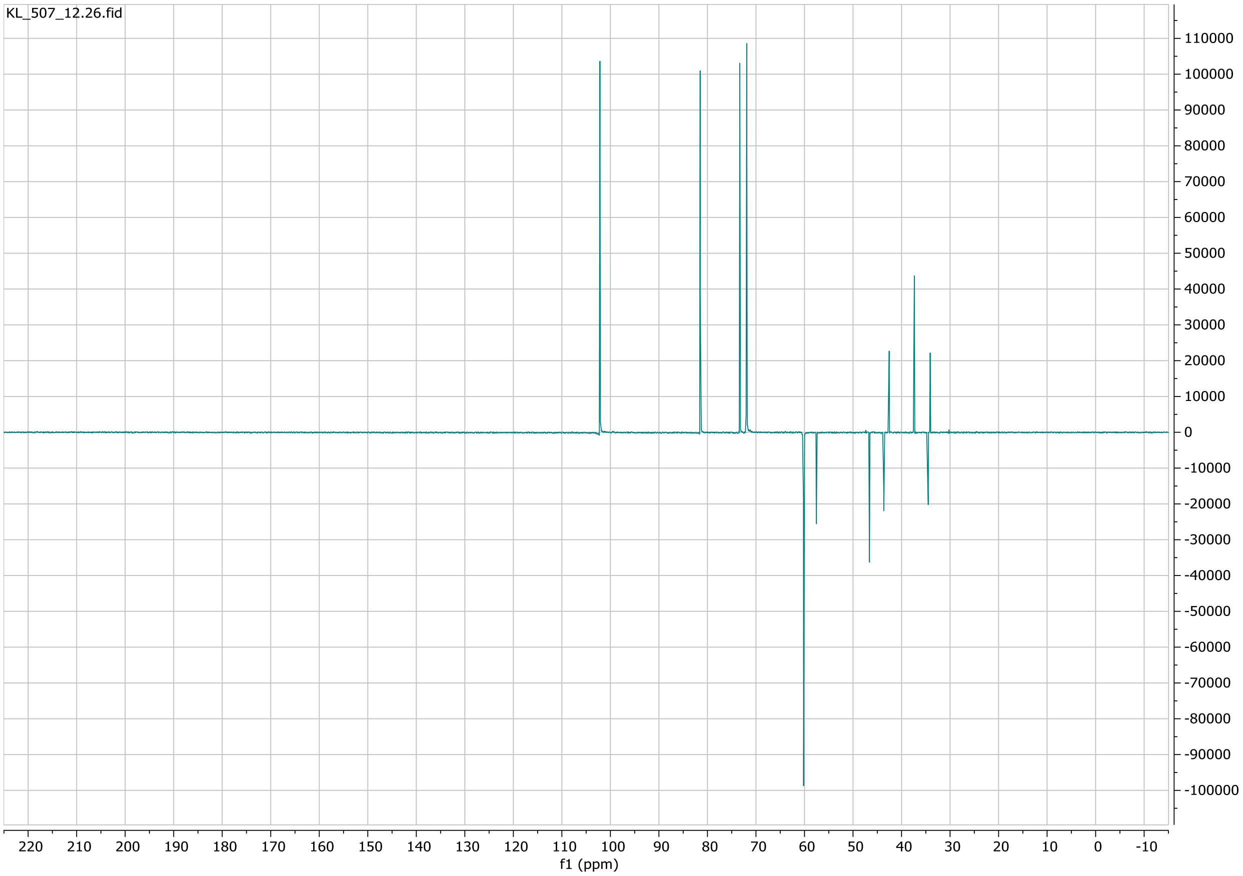

Figure S24 The ^{13}C DEPT spectra of **3** (15 mM) water solution in the presence of β-CD (15 mM) (no buffer)

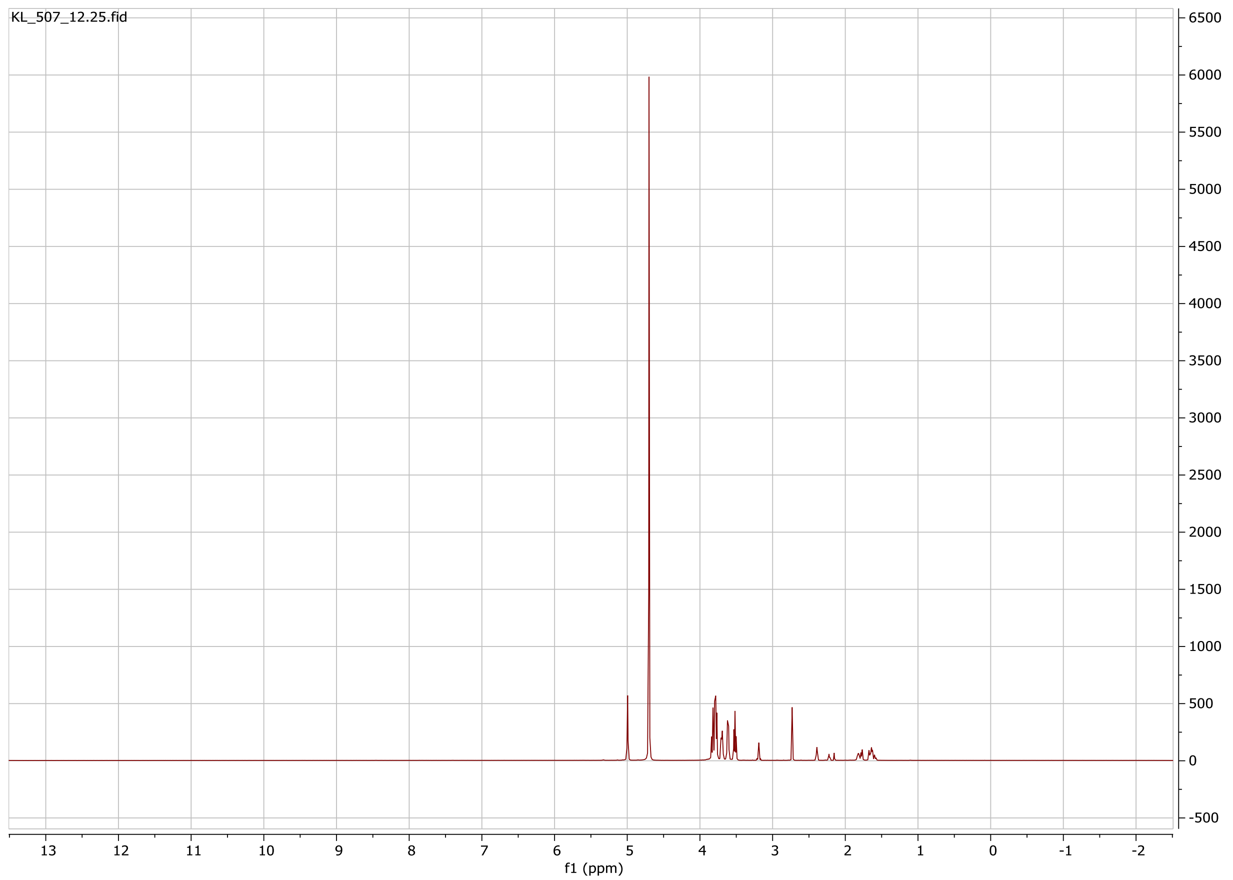

Figure S25 The ^{1}H NMR spectra of **3** (15 mM) water solution in the presence of β-CD (15 mM) (no buffer)

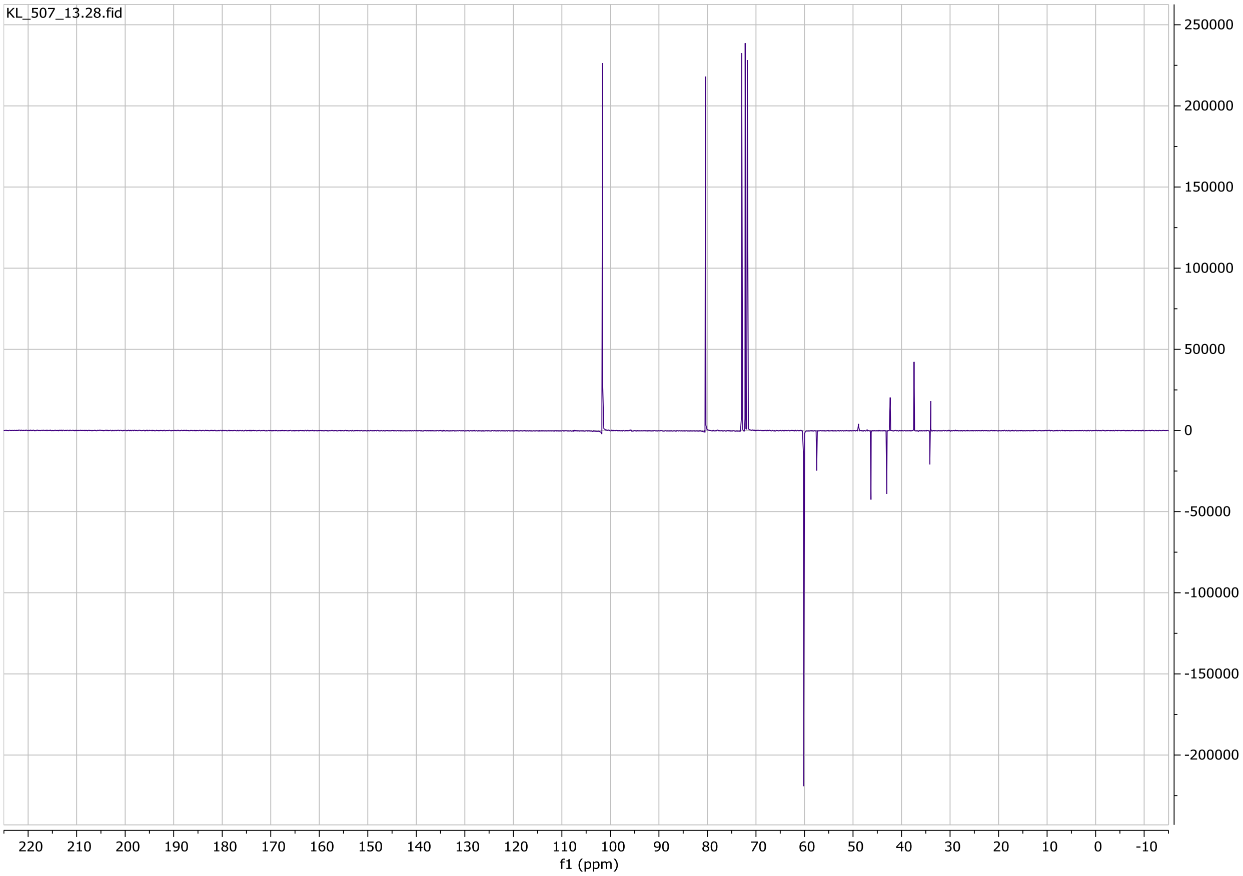

Figure S26 The ^{13}C DEPT spectra of **3** (15 mM) water solution in the presence of γ-CD (30 mM) (no buffer)

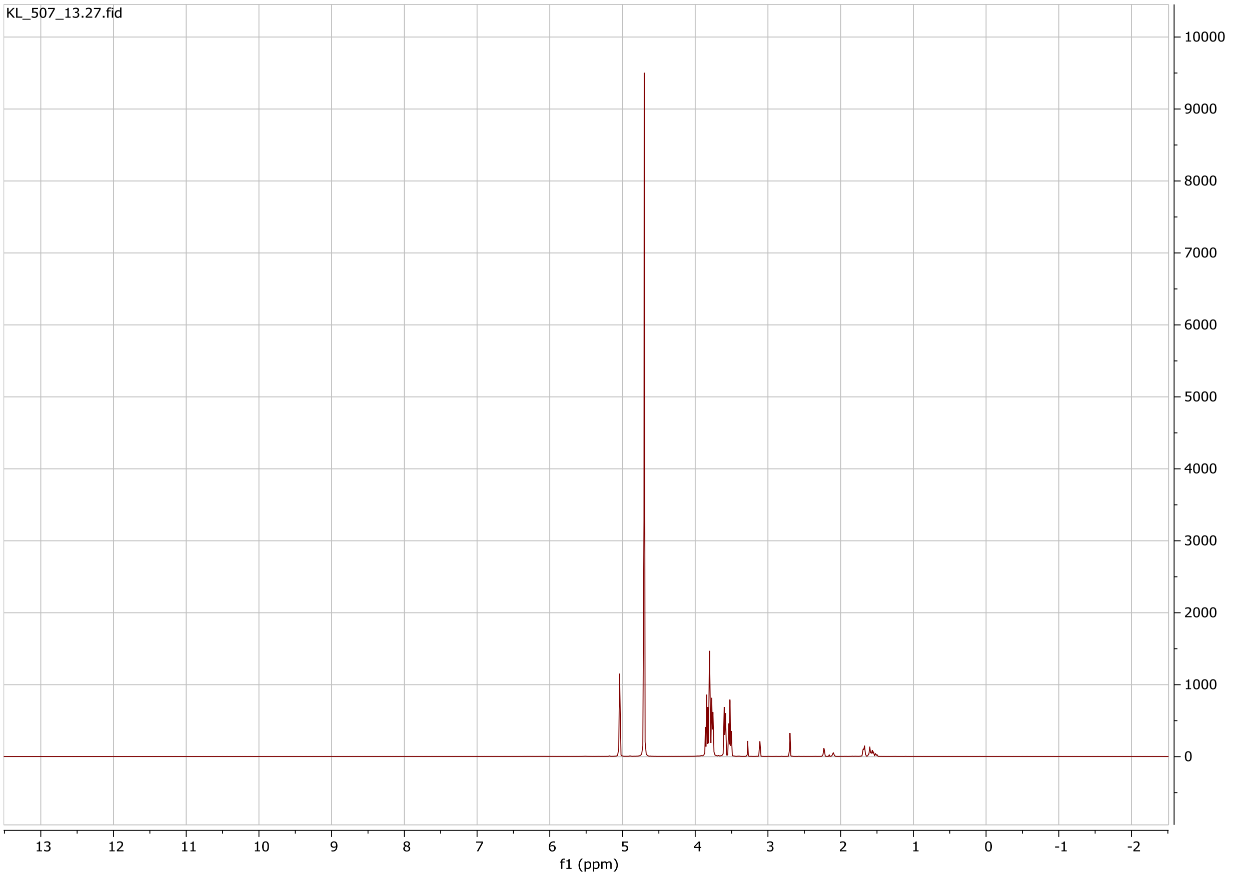

Figure S27 The ^{1}H NMR spectra of **3** (15 mM) water solution in the presence of γ-CD (30 mM) (no buffer)

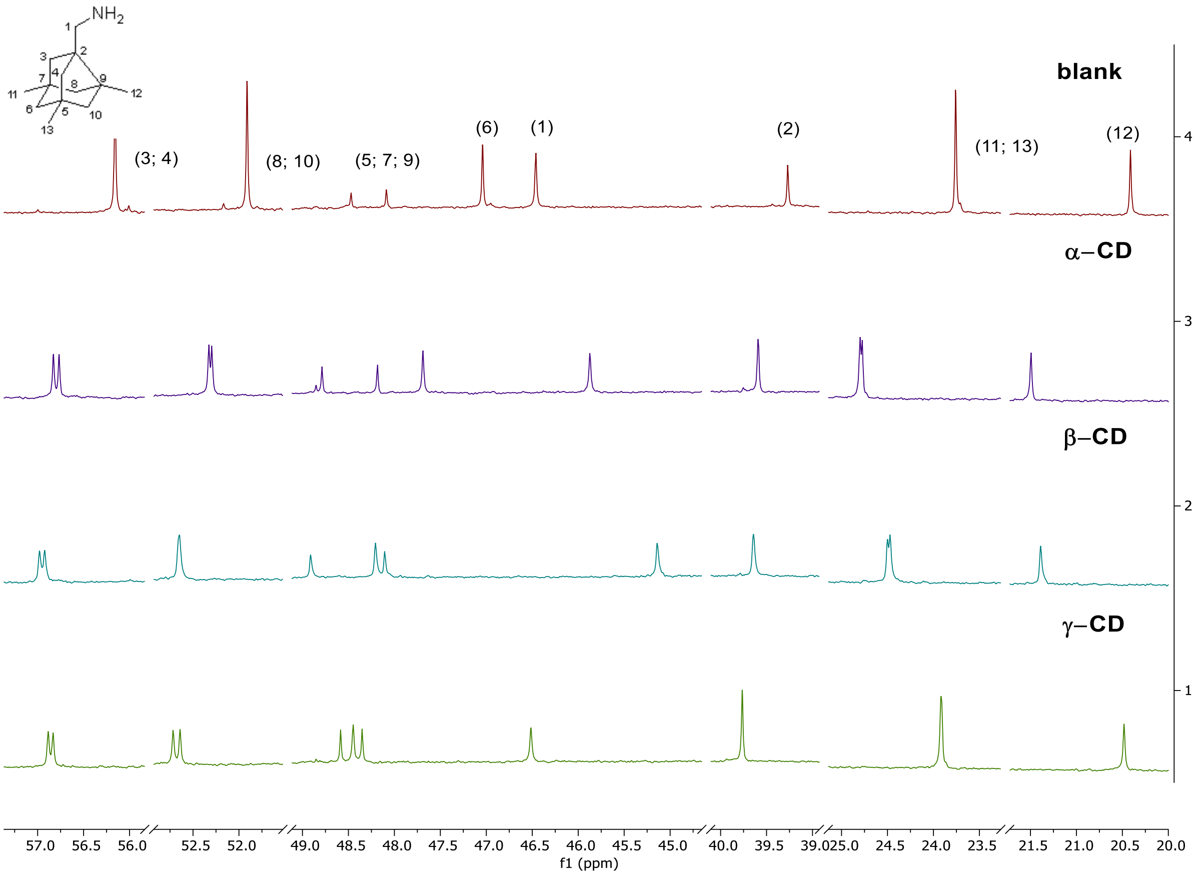

Figure S28 The partial ^{13}C NMR spectra of **4** (6 mM) water solution in the absence of any host (blank); in the presence of α-CD (12 mM); β-CD (6 mM); γ-CD (12 mM) (no buffer)

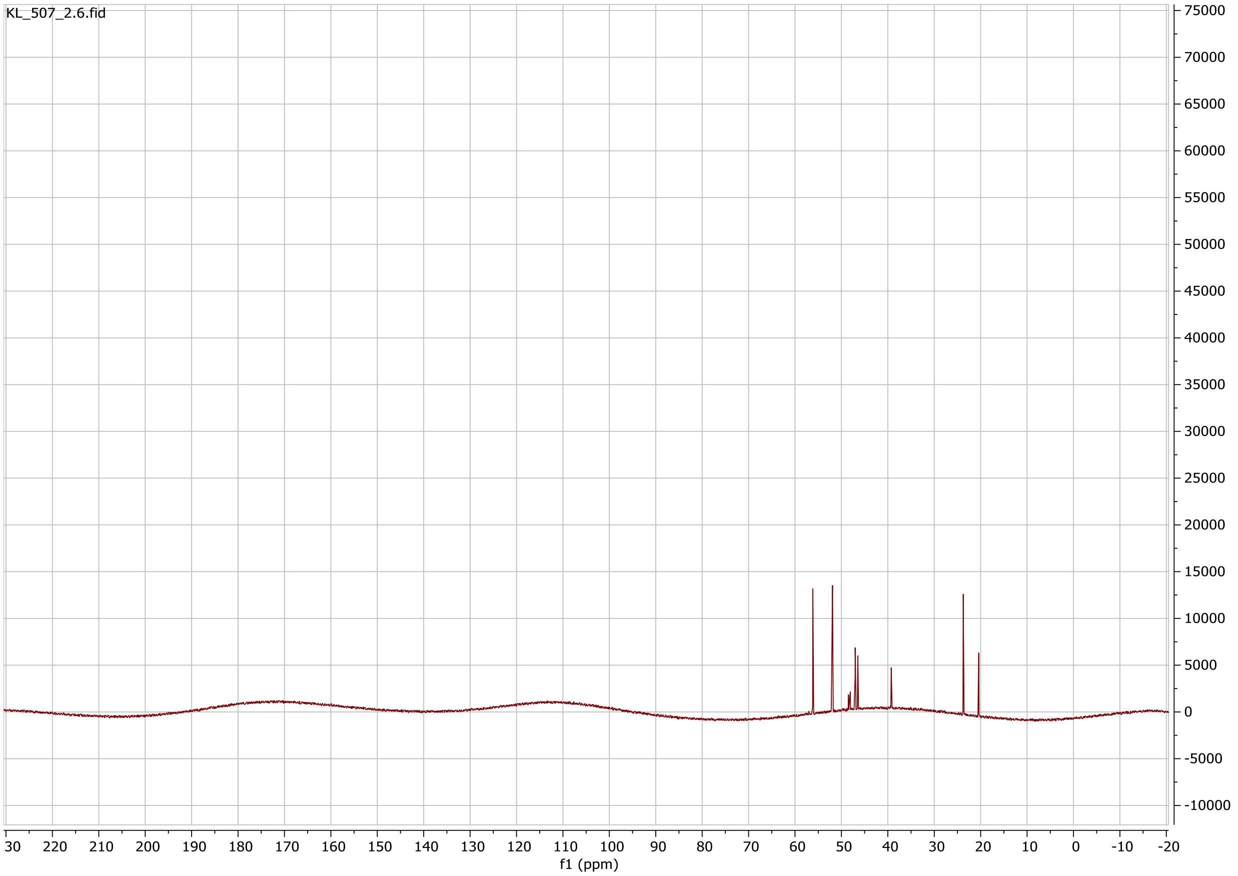

Figure S29 The ^{13}C NMR spectra of **4** (6 mM) water solution (no buffer).

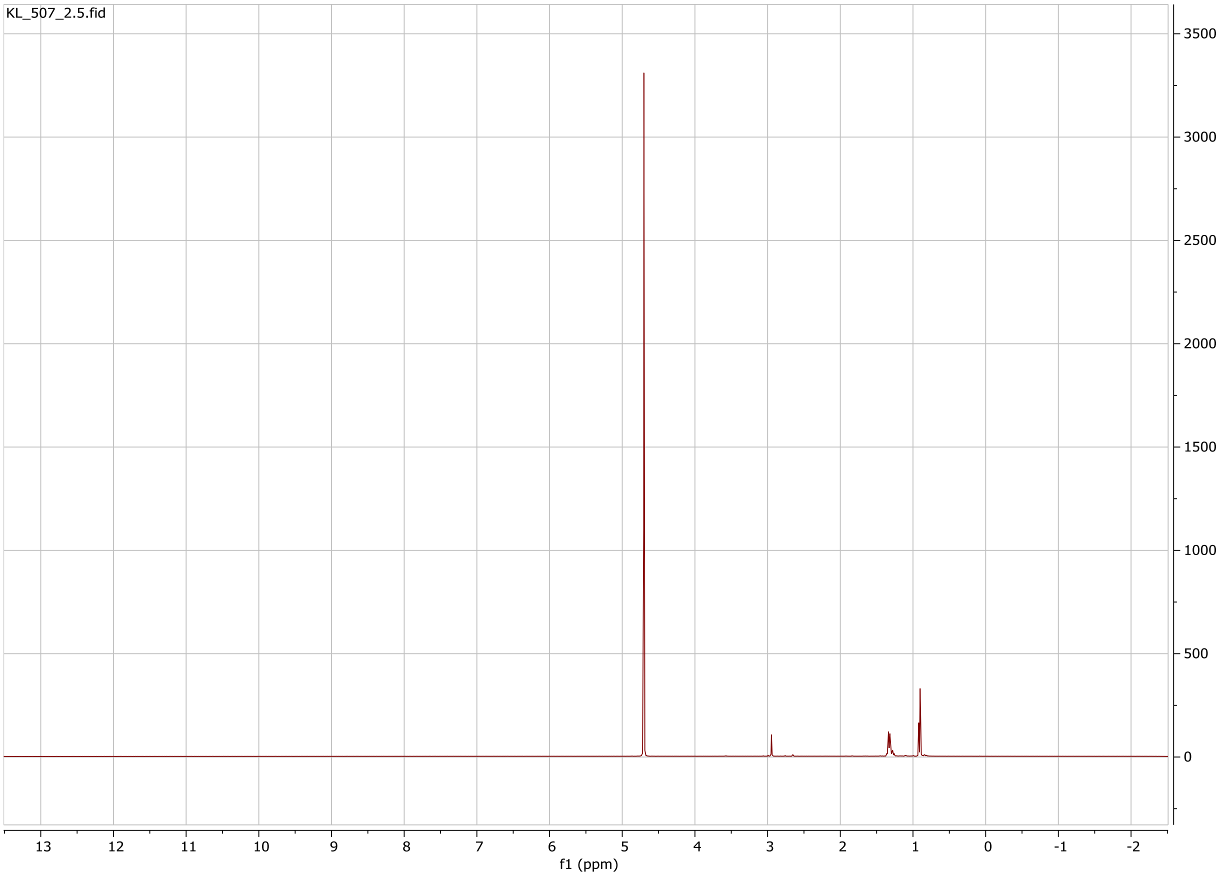

Figure S30 The ^{1}H NMR spectra of **4** (6 mM) water solution (no buffer).

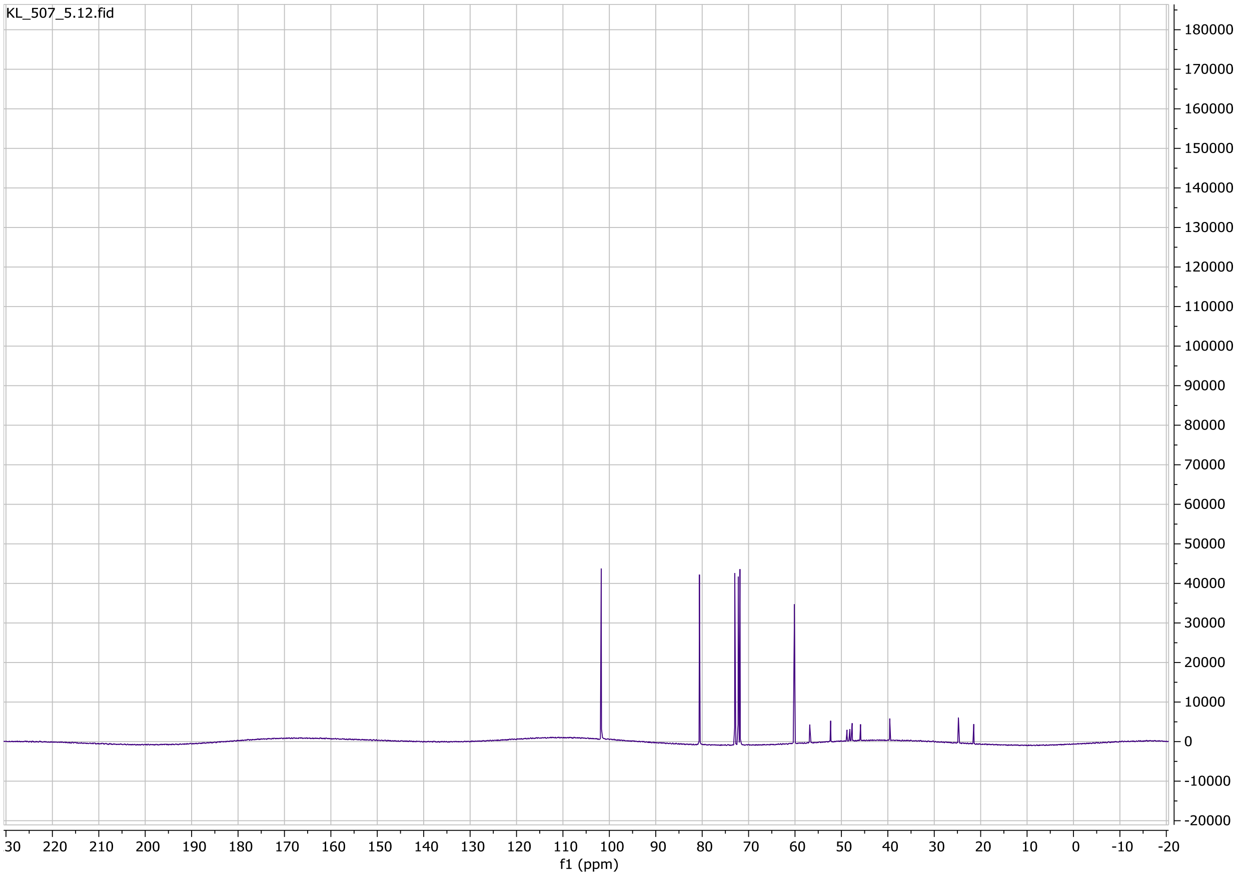

Figure S31 The ^{13}C NMR spectra of **4** (6 mM) water solution in the presence of α-CD (12 mmol) (no buffer).

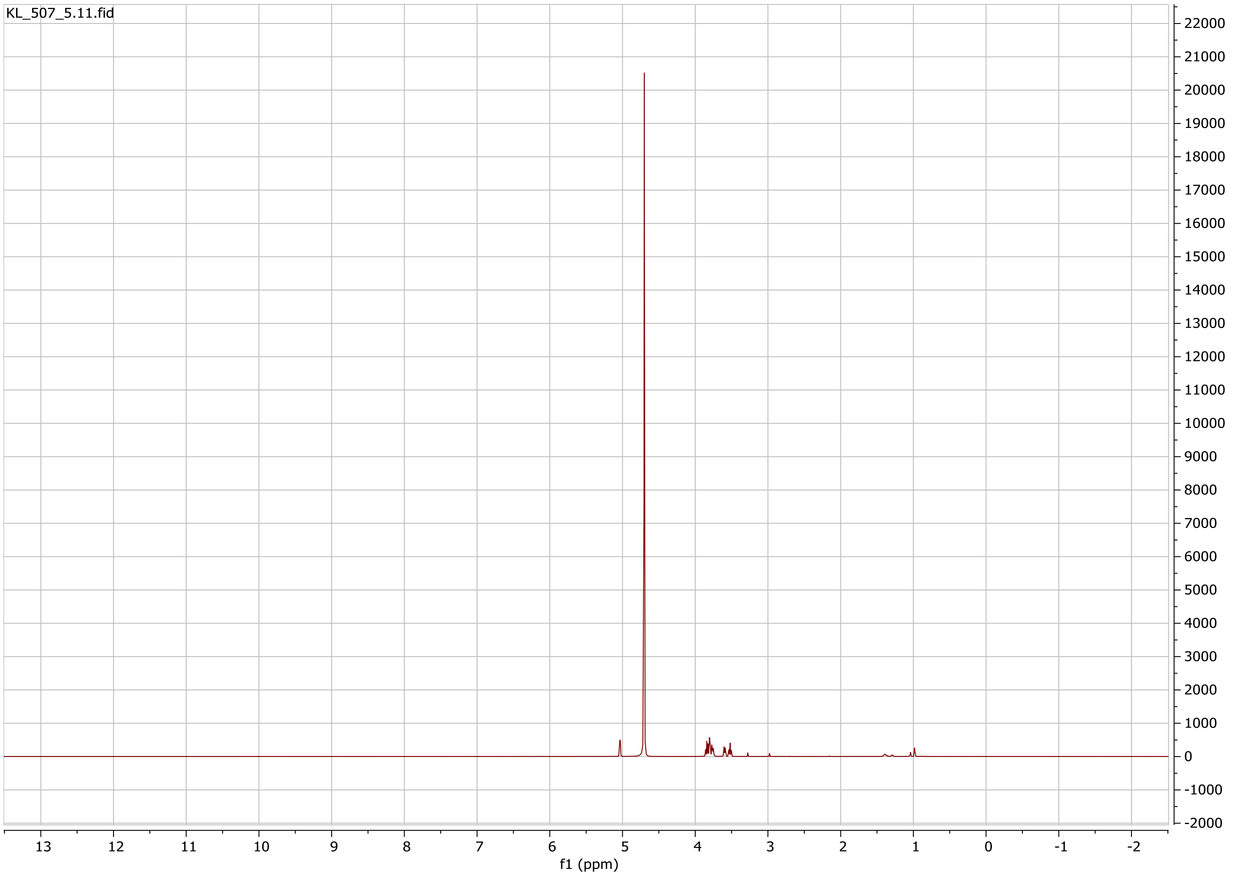

Figure S32 The ^{1}H NMR spectra of **4** (6 mM) water solution in the presence of α-CD (12 mmol) (no buffer).

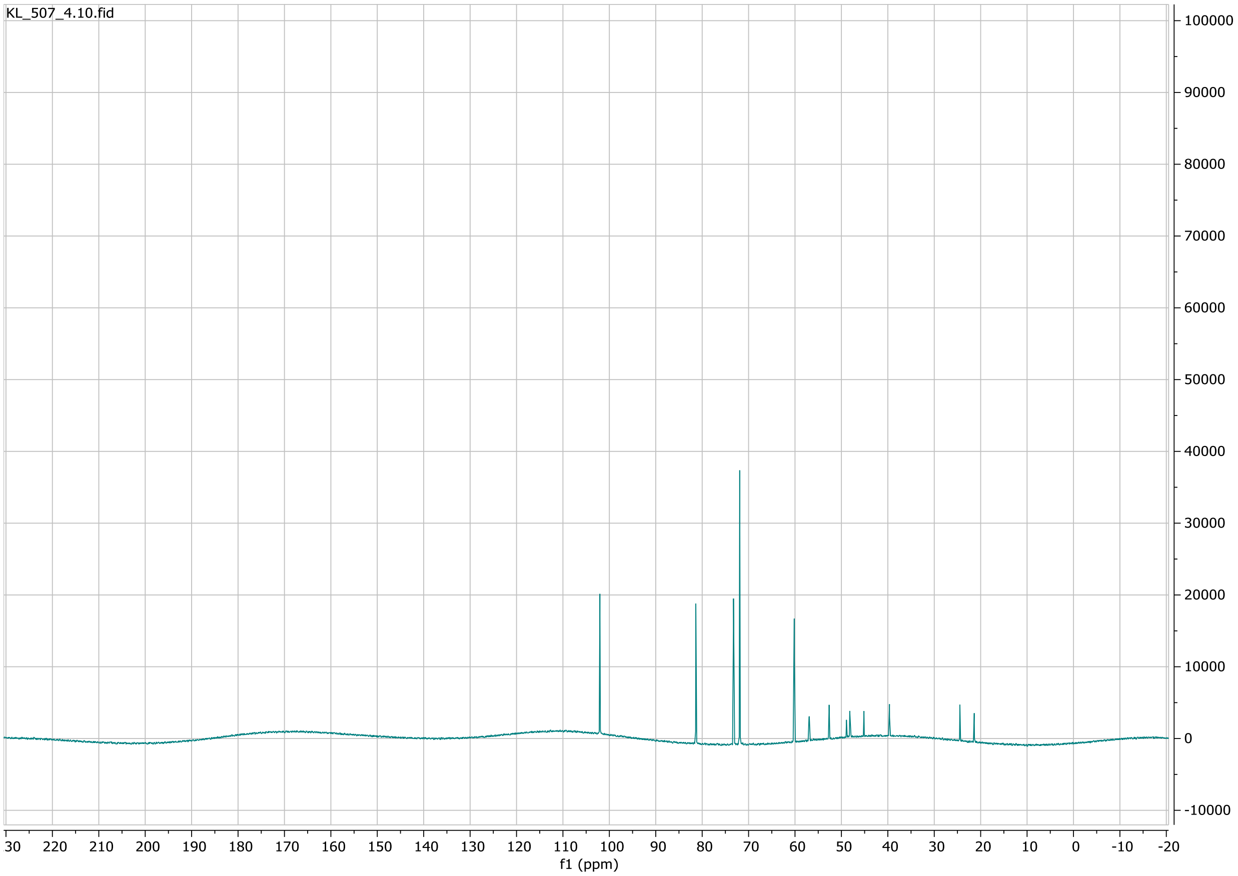

Figure S33 The ^{13}C NMR spectra of **4** (6 mM) water solution in the presence of β-CD (6 mmol) (no buffer).

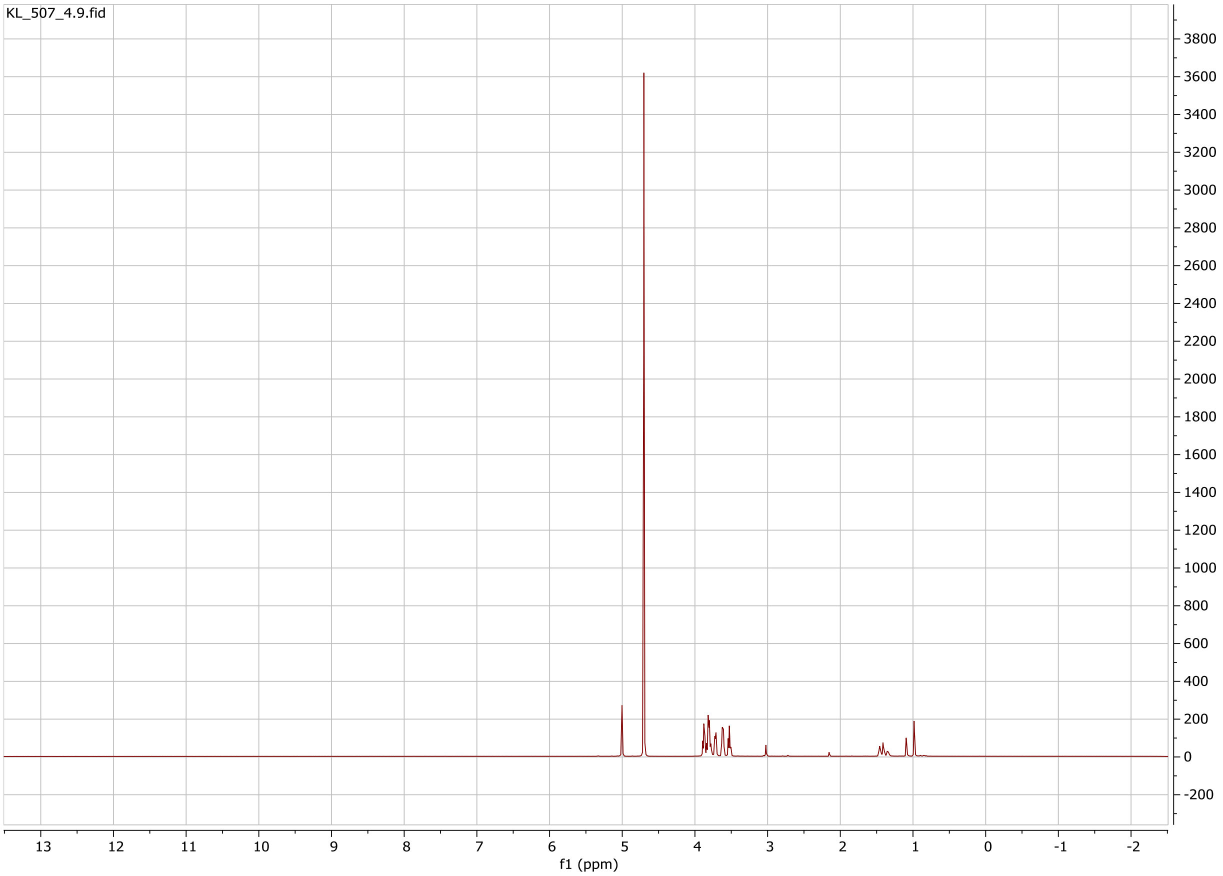

Figure S34 The ^{13}C NMR spectra of **4** (6 mM) water solution in the presence of γ-CD (12 mmol) (no buffer).

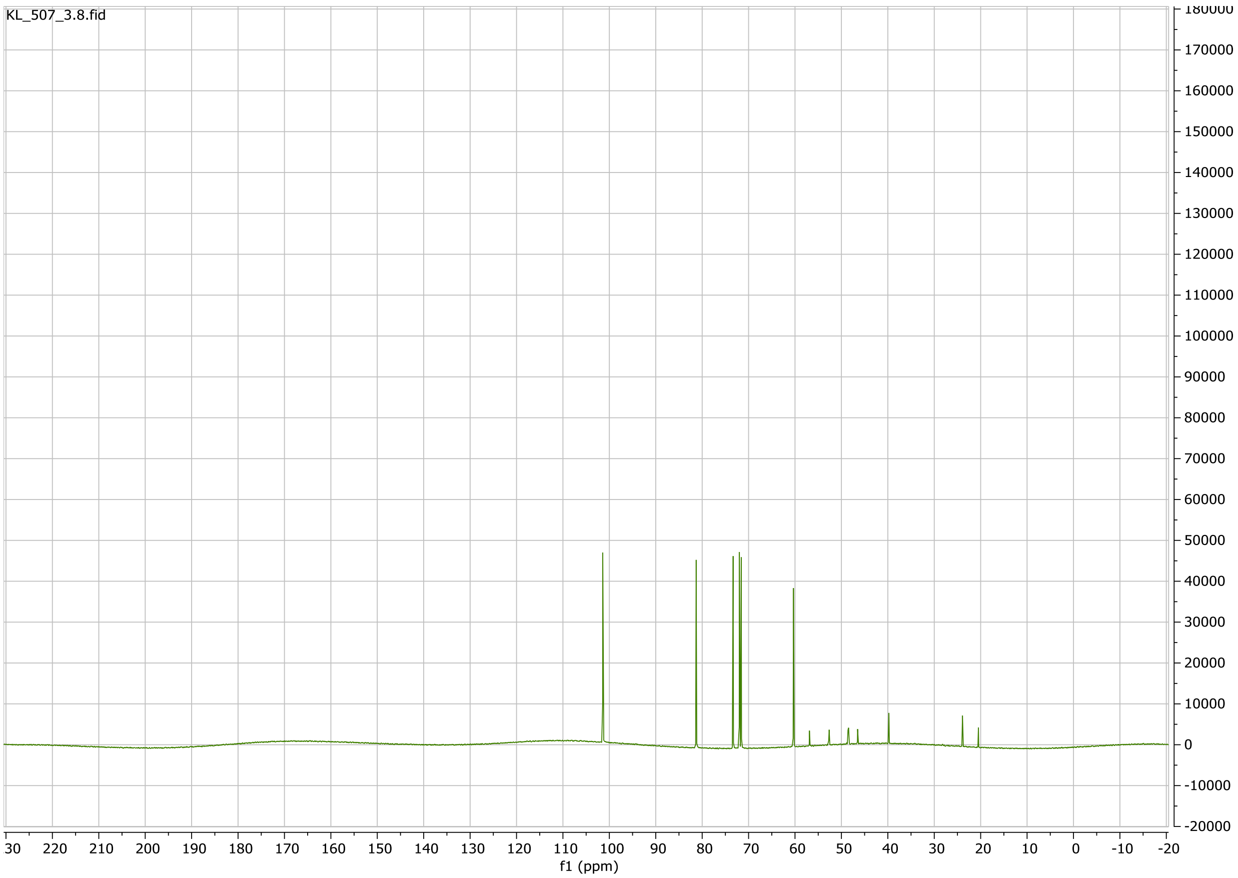

Figure S35 The ^{13}C NMR spectra of **4** (6 mM) water solution in the presence of γ-CD (12 mmol) (no buffer).

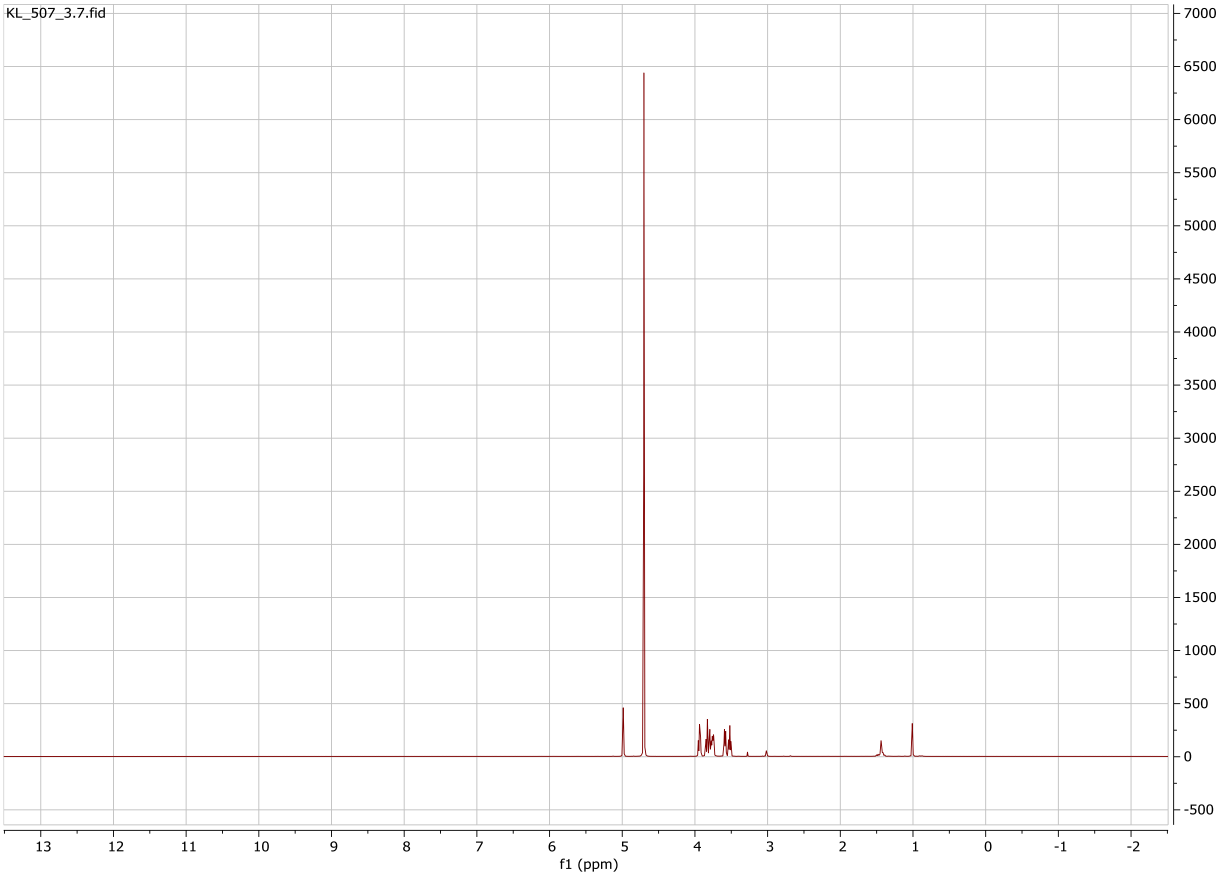

Figure S36 The ^{1}H NMR spectra of **4** (6 mM) water solution in the presence of γ-CD (12 mmol) (no buffer).

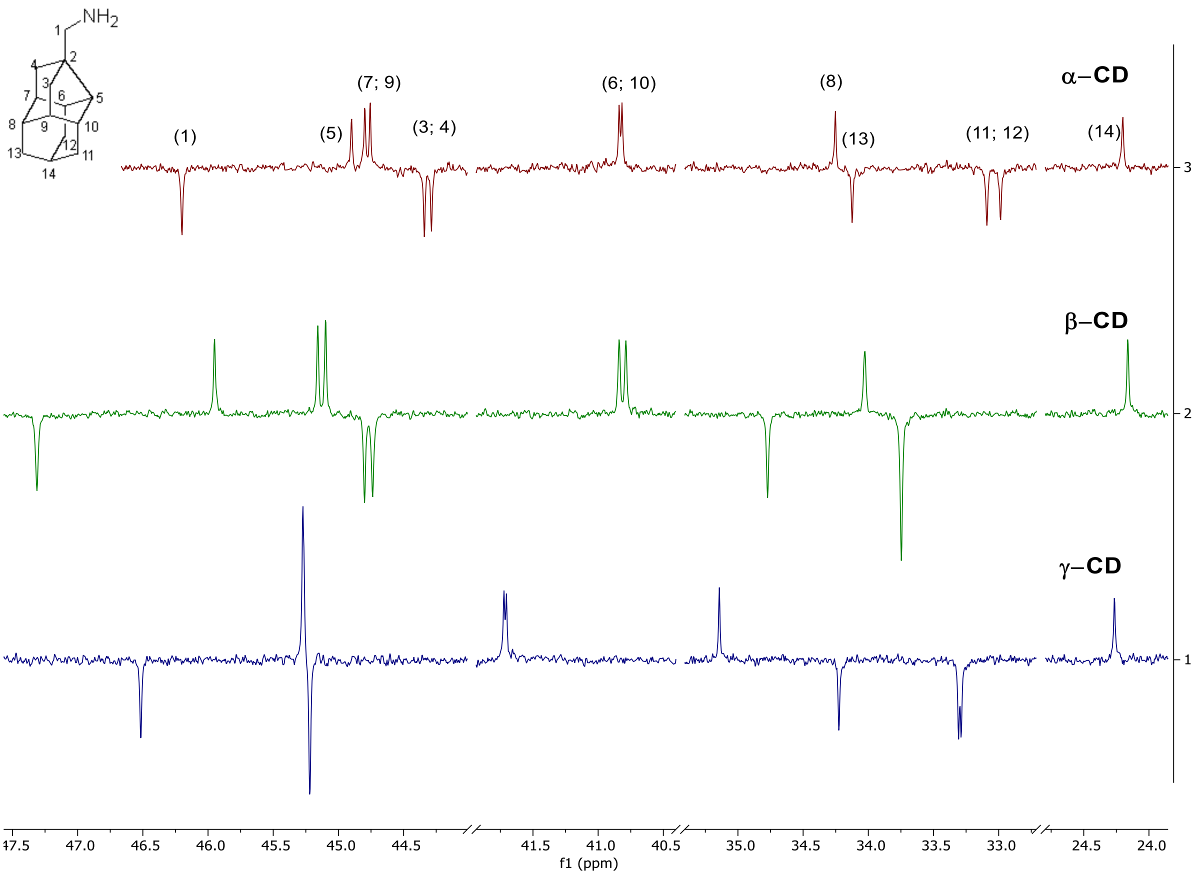

Figure S37 The partial ^{13}C NMR spectra of **5** (5 mM) water solution in the presence of α-CD (10 mM); β-CD (5 mM); γ-CD (10 mM) (no buffer)

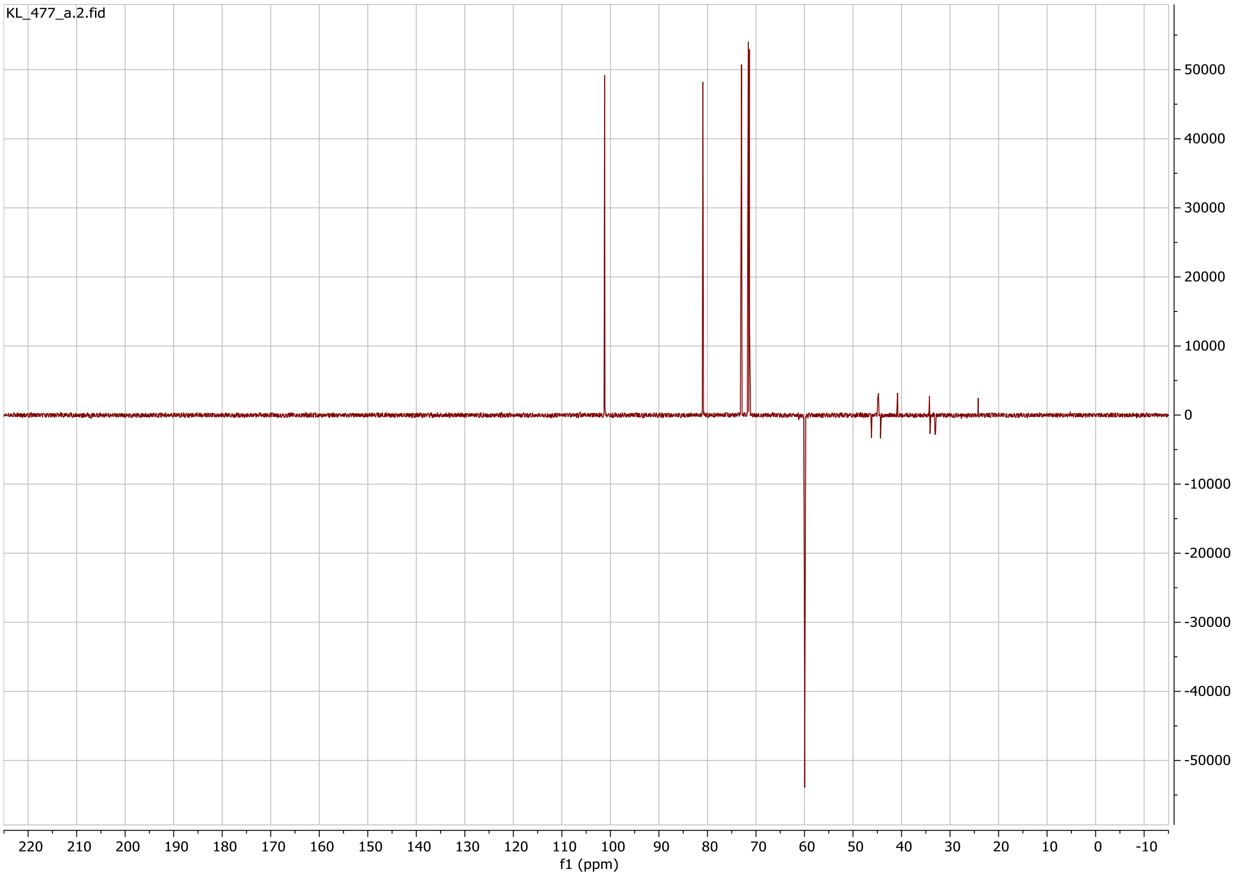

Figure S38 The ^{13}C DEPT spectra of **5** (5 mM) water solution in the presence of α-CD (10 mM) (no buffer)

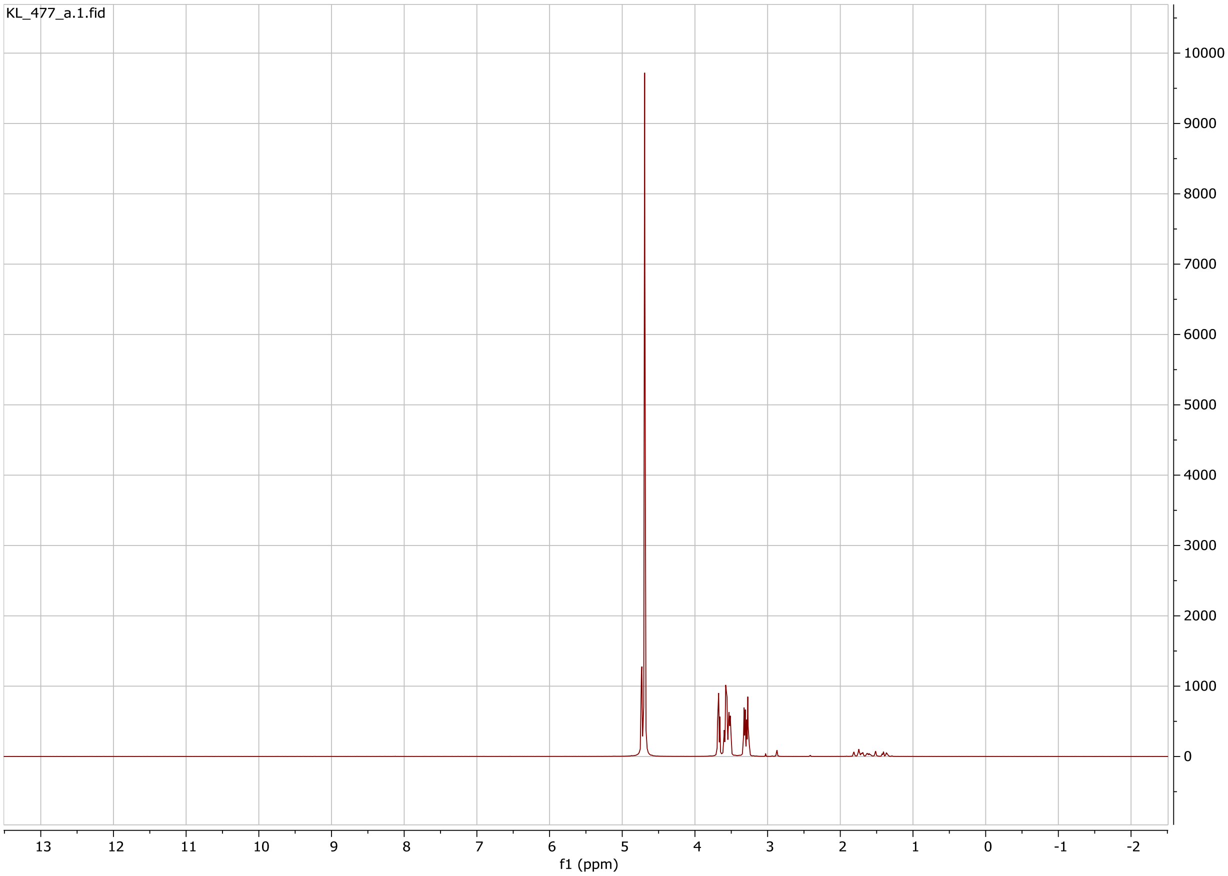

Figure S39 The ^{1}H NMR spectra of **5** (5 mM) water solution in the presence of α-CD (10 mM) (no buffer)

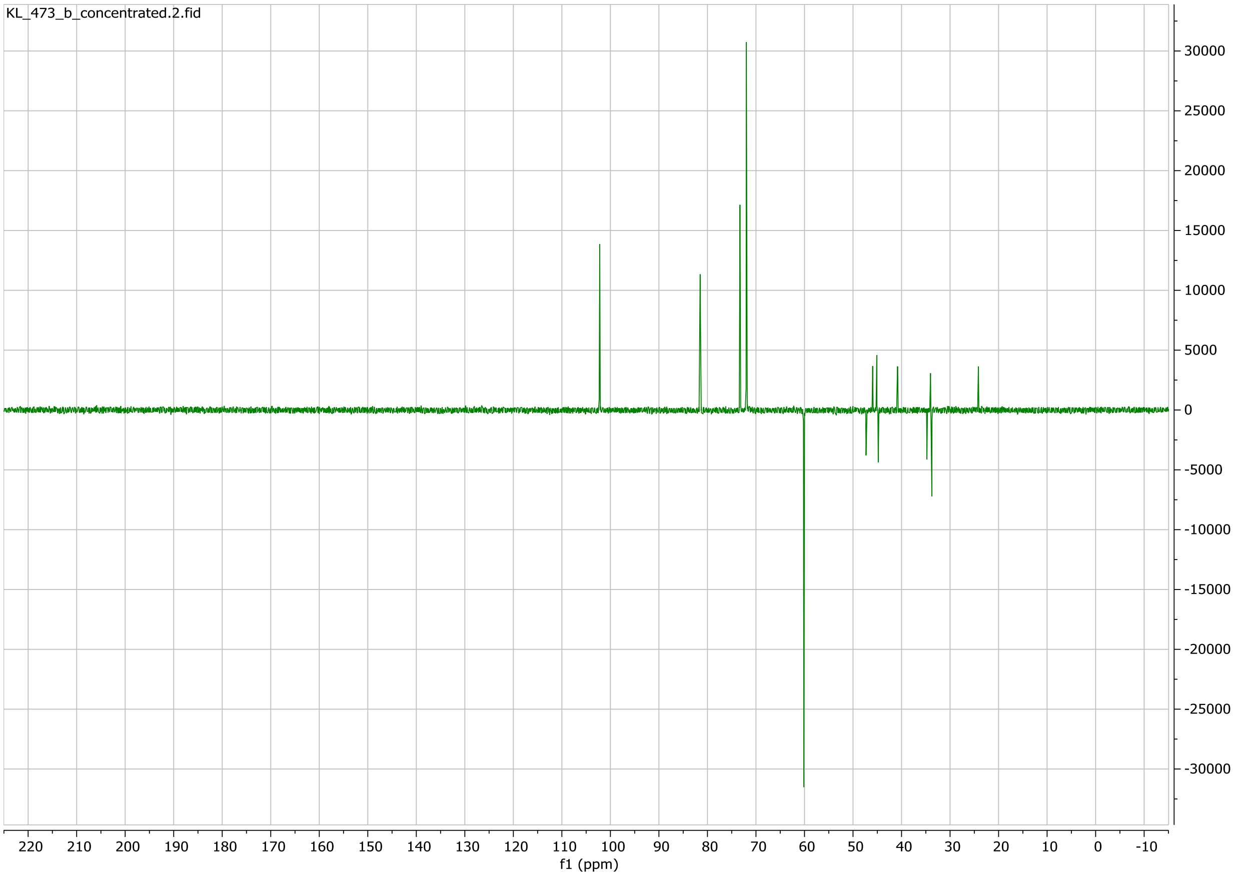

Figure S40 The ^{13}C DEPT spectra of **5** (5 mM) water solution in the presence of β-CD (5 mM) (no buffer).

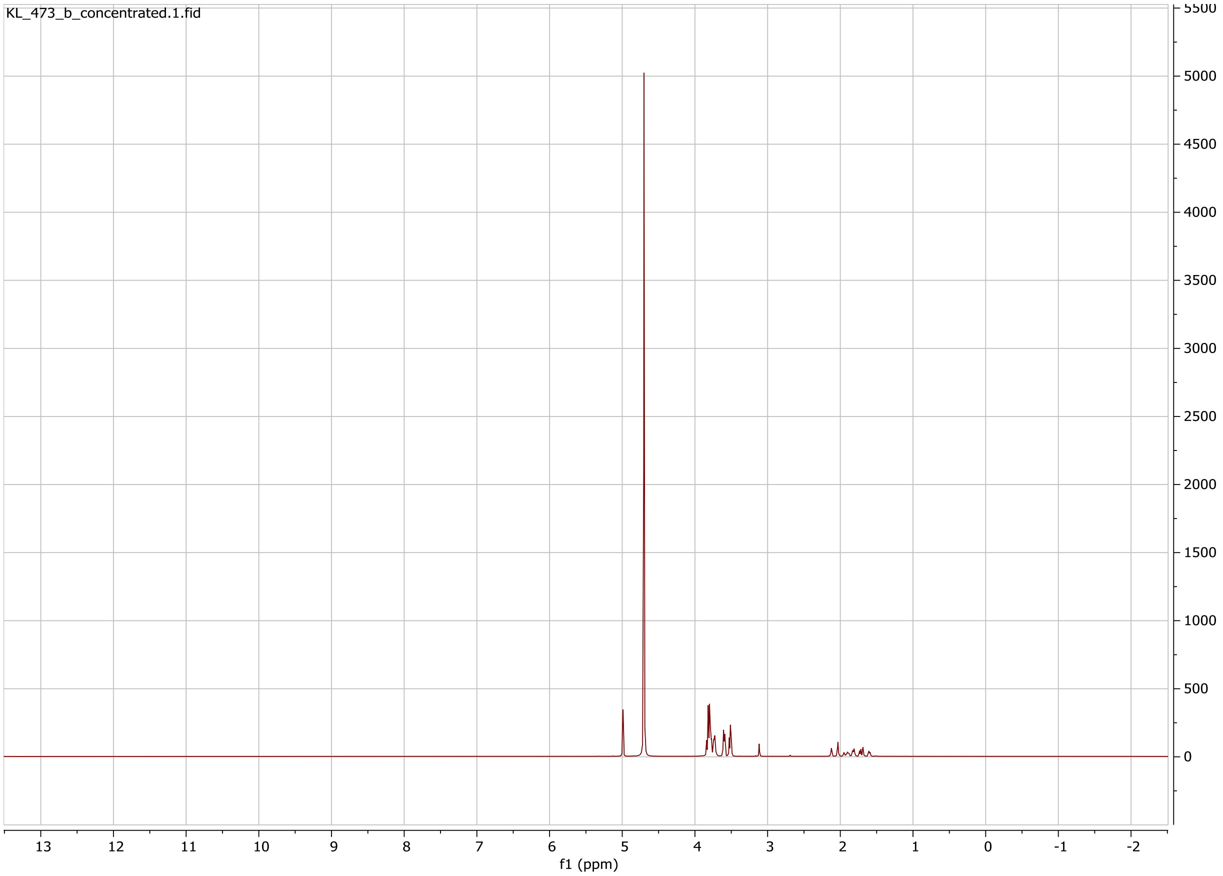

Figure S41 The ^{1}H NMR spectra of **5** (5 mM) water solution in the presence of β-CD (5 mM) (no buffer).

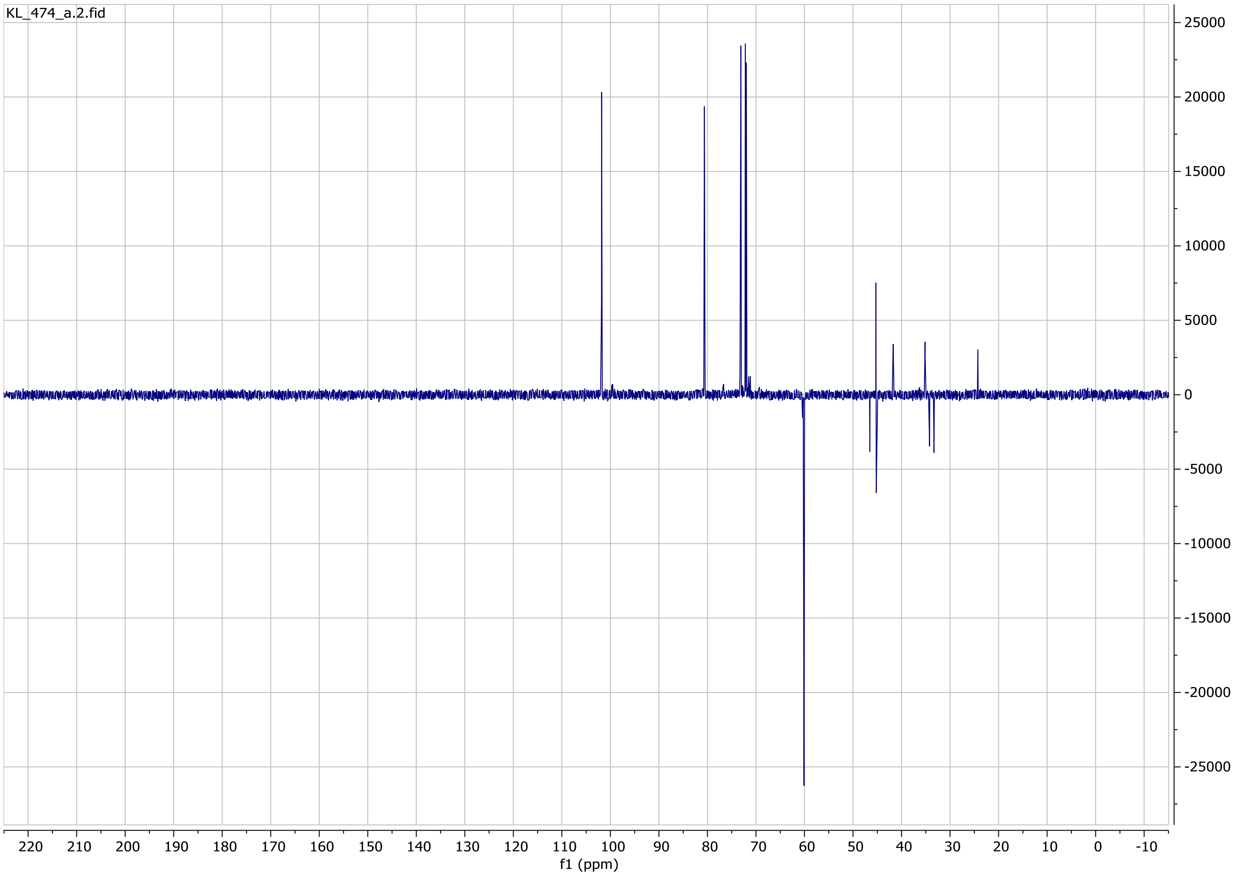

Figure S42 The ^{13}C DEPT spectra of **5** (5 mM) water solution in the presence of γ-CD (10 mM) (no buffer).

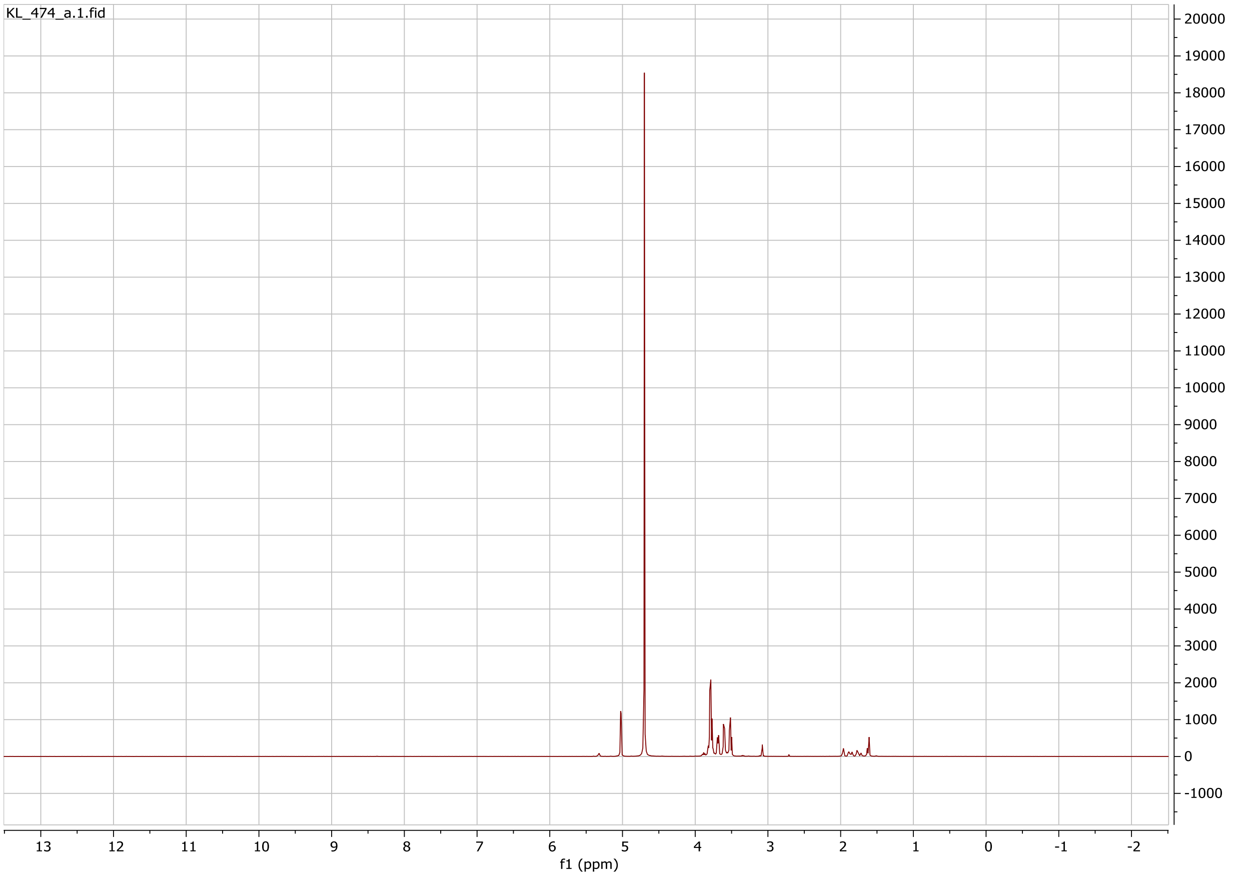

Figure S43 The ^{1}H NMR spectra of **5** (5 mM) water solution in the presence of γ-CD (10 mM) (no buffer).

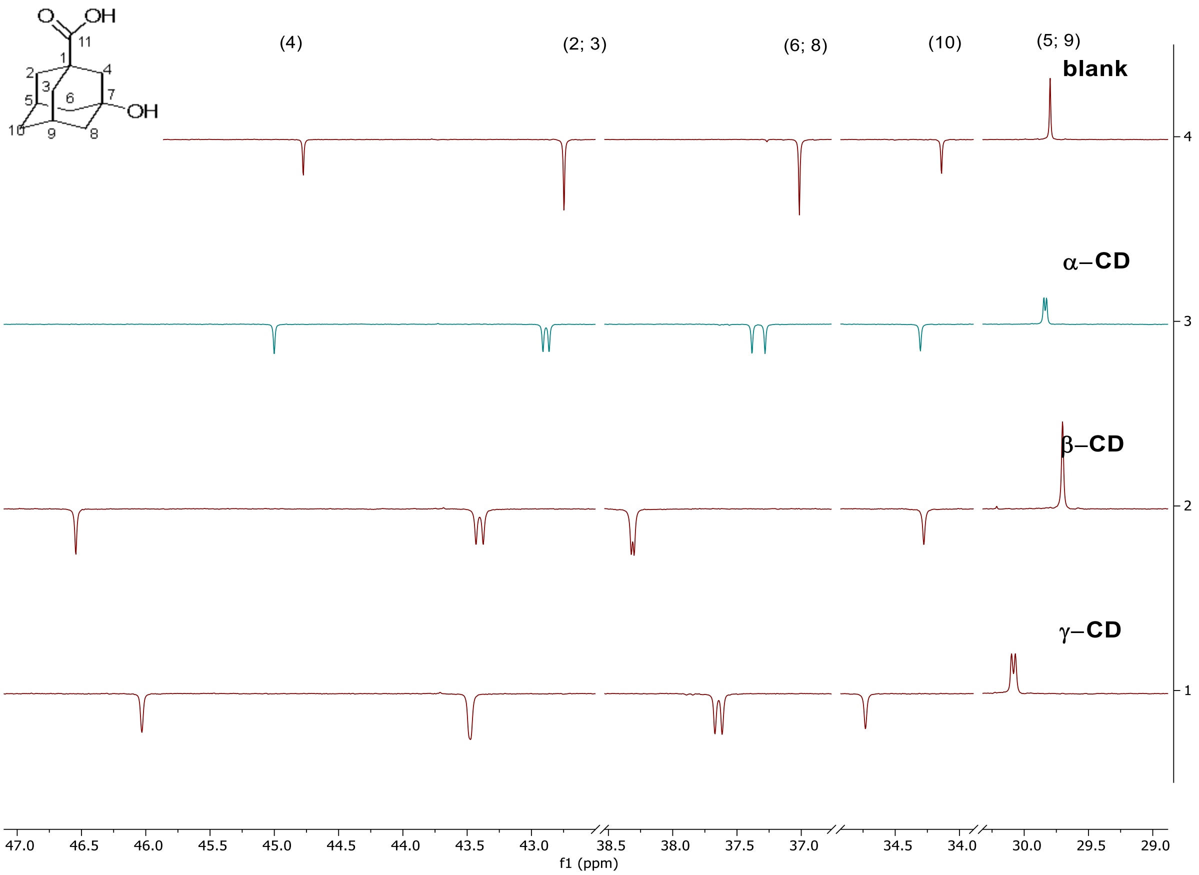

Figure S44 The partial ^{13}C DEPT spectra of **6** (14 mM) water solution in the absence of any host (blank); in the presence of α-CD (28 mM); β-CD (14 mM); γ-CD (28 mM) (no buffer)

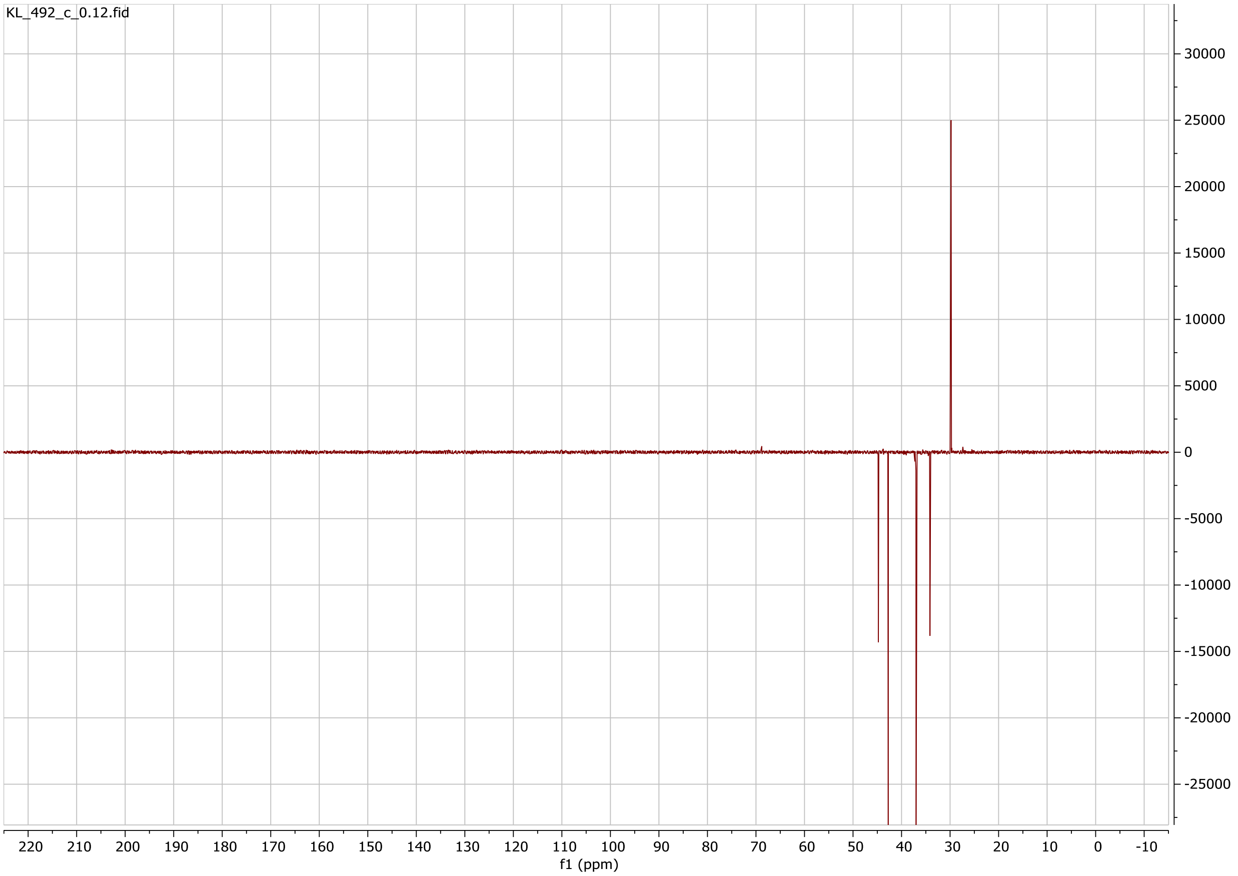

Figure S45 The ^{13}C DEPT spectra of **6** (14 mM) water solution (no buffer).

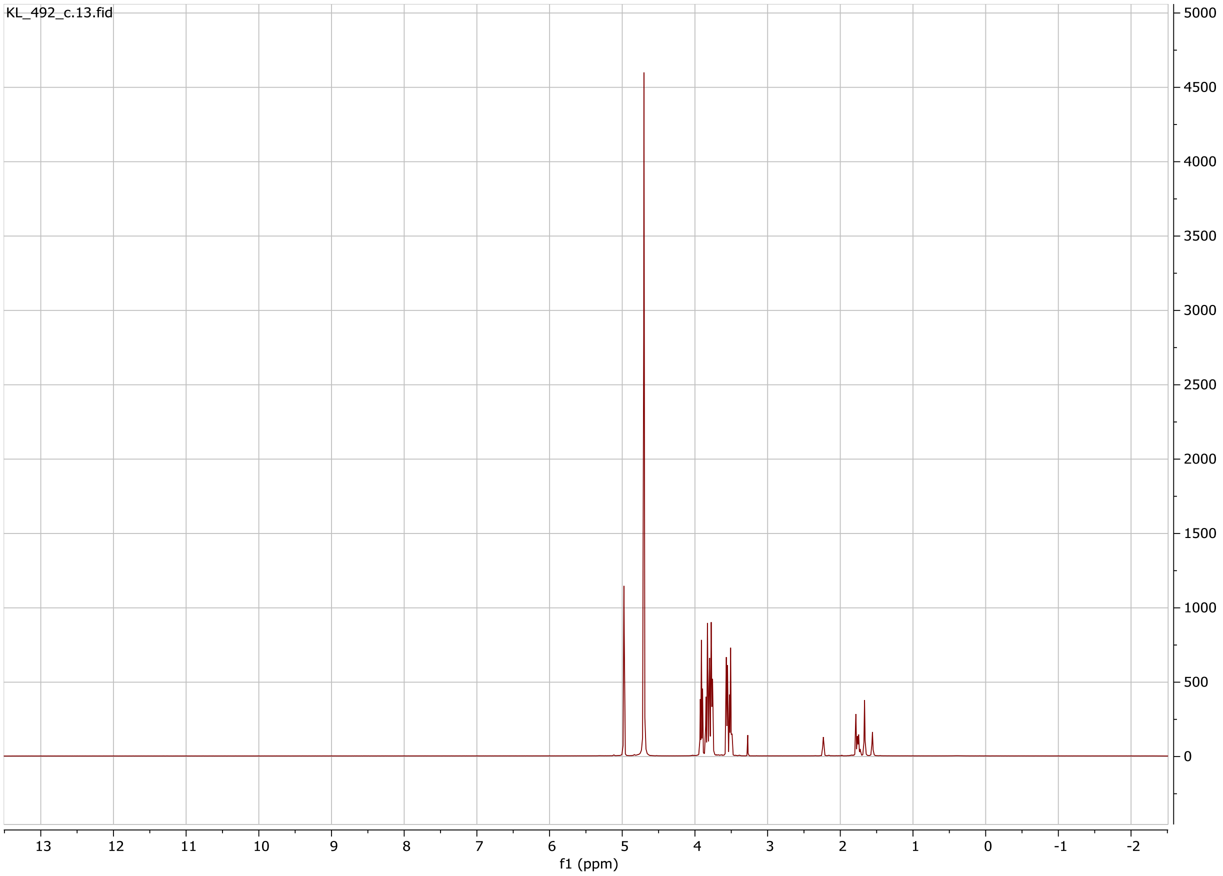

Figure S46 The ^{1}H NMR spectra of **6** (14 mM) water solution (no buffer).

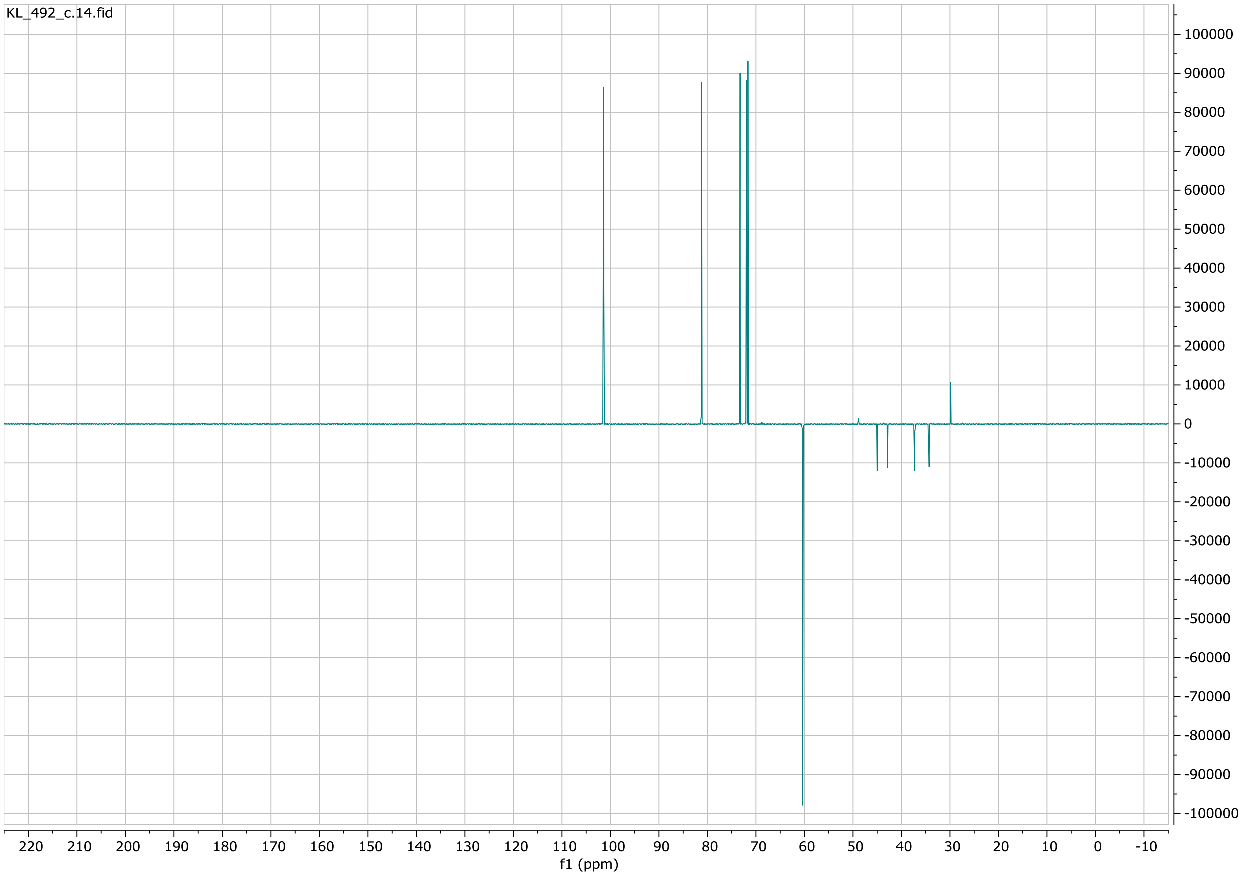

Figure S47 The ^{13}C DEPT spectra of **6** (14 mM) water solution in the presence of α-CD (28 mM) (no buffer).

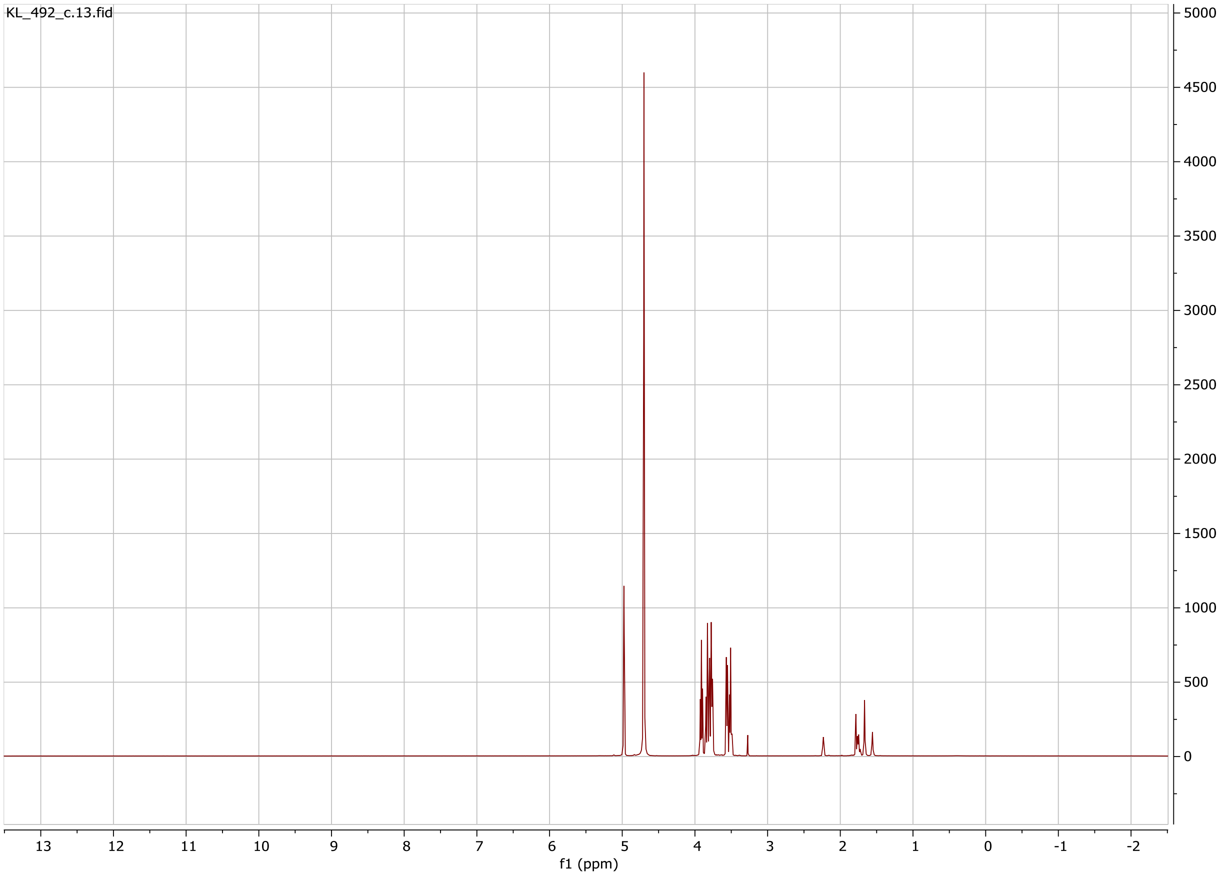

Figure S48 The ^{1}H NMR spectra of **6** (14 mM) water solution in the presence of α-CD (28 mM) (no buffer).

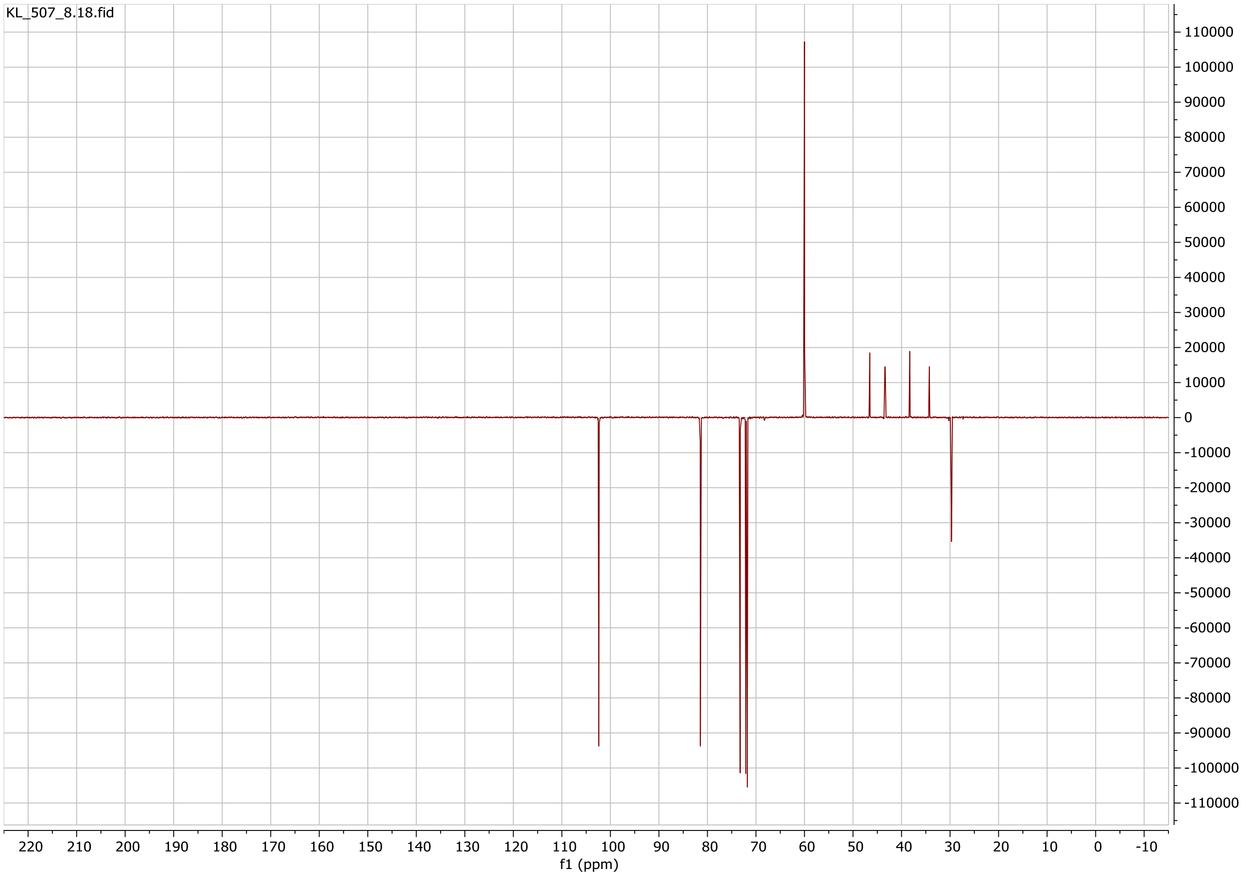

Figure S49 The ^{13}C DEPT spectra of **6** (14 mM) water solution in the presence of β-CD (14 mM) (no buffer).

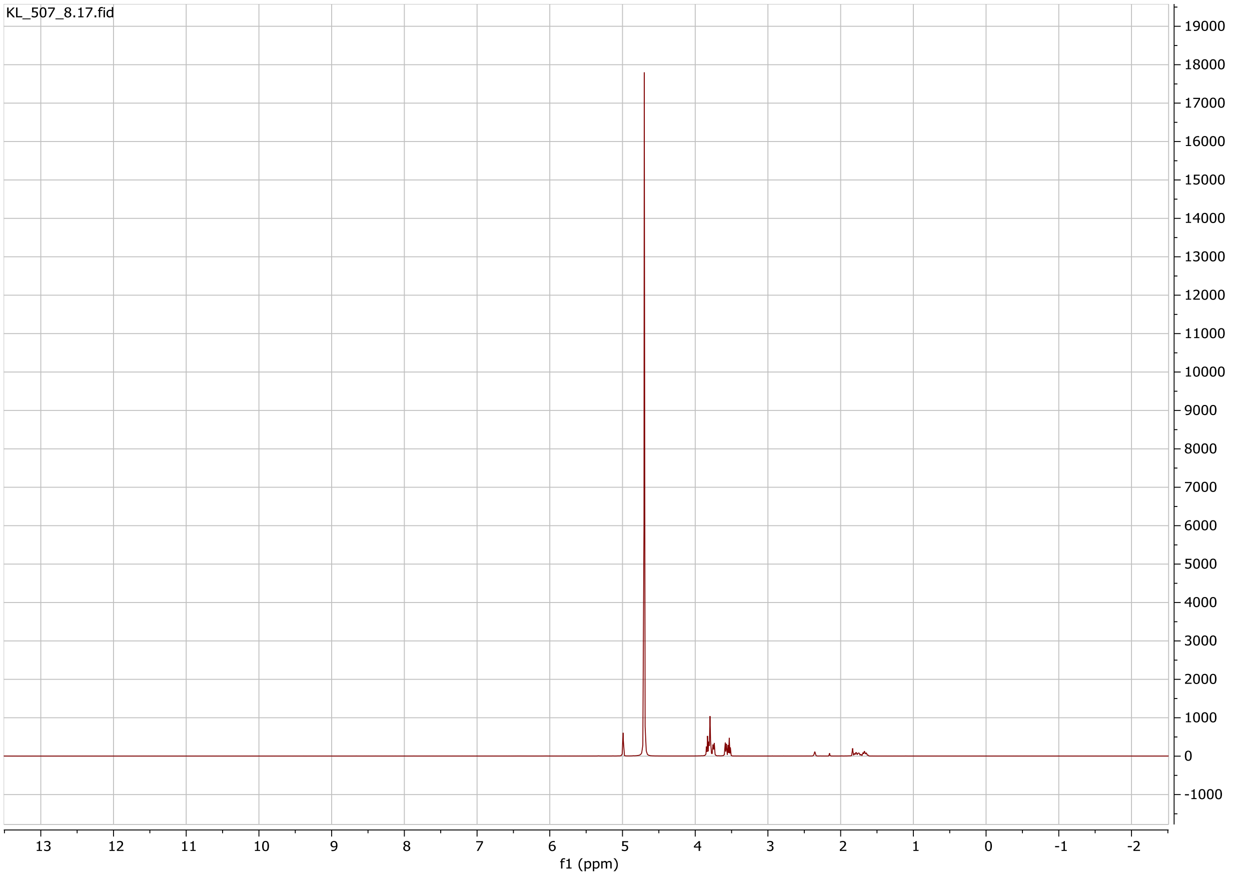

Figure S50 The ^{1}H NMR spectra of **6** (14 mM) water solution in the presence of β-CD (14 mM) (no buffer).

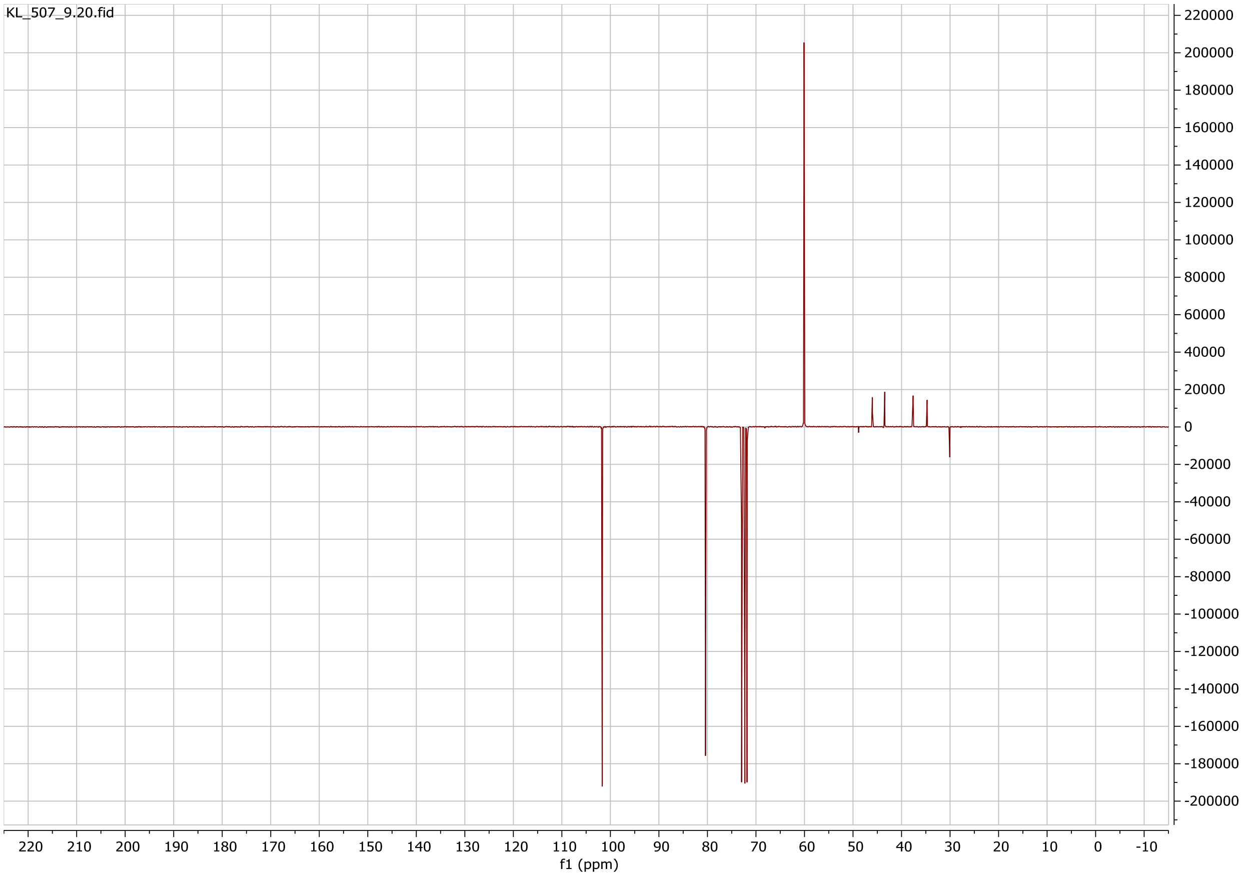

Figure S51 The ^{13}C DEPT spectra of **6** (14 mM) water solution in the presence of γ-CD (28 mM) (no buffer).

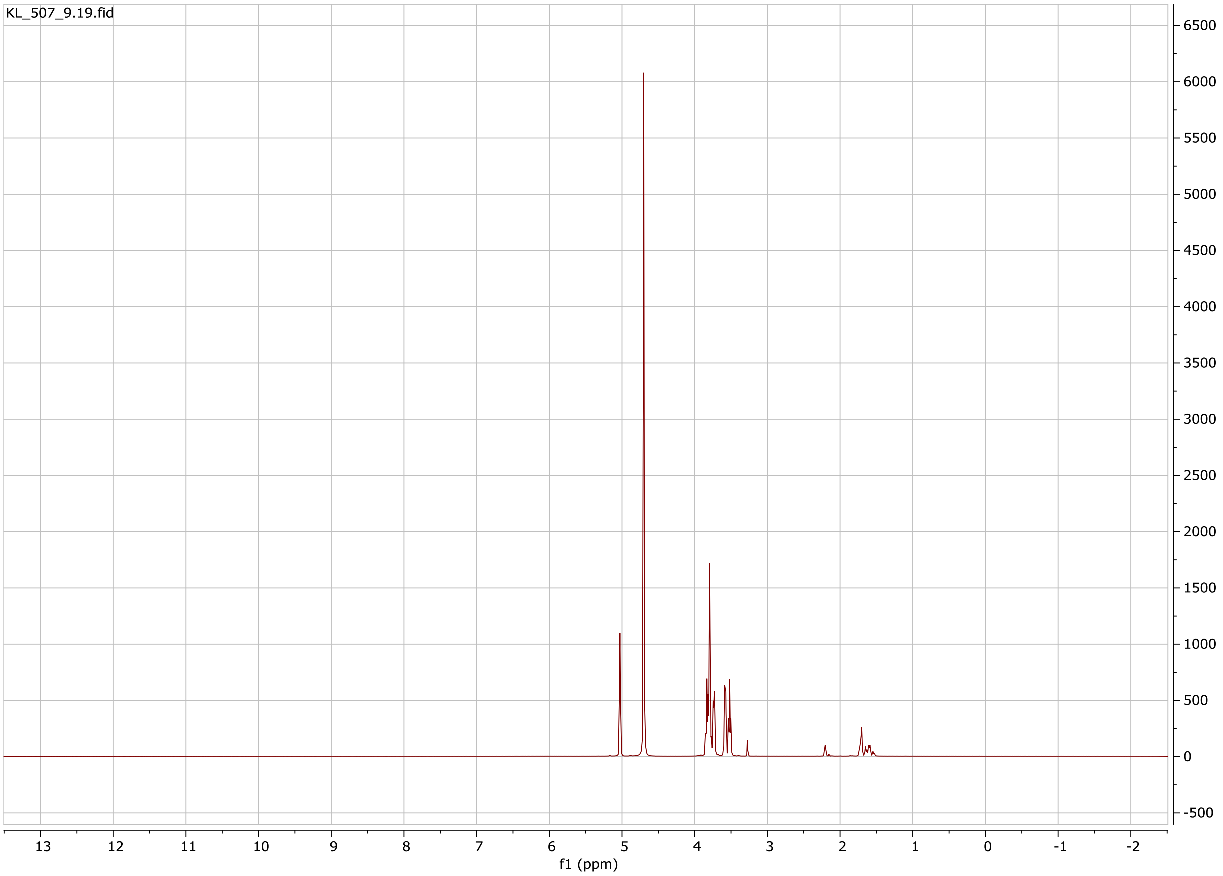

Figure S52 The ^{13}C NMR spectra of **6** (14 mM) water solution in the presence of γ-CD (28 mM) (no buffer).

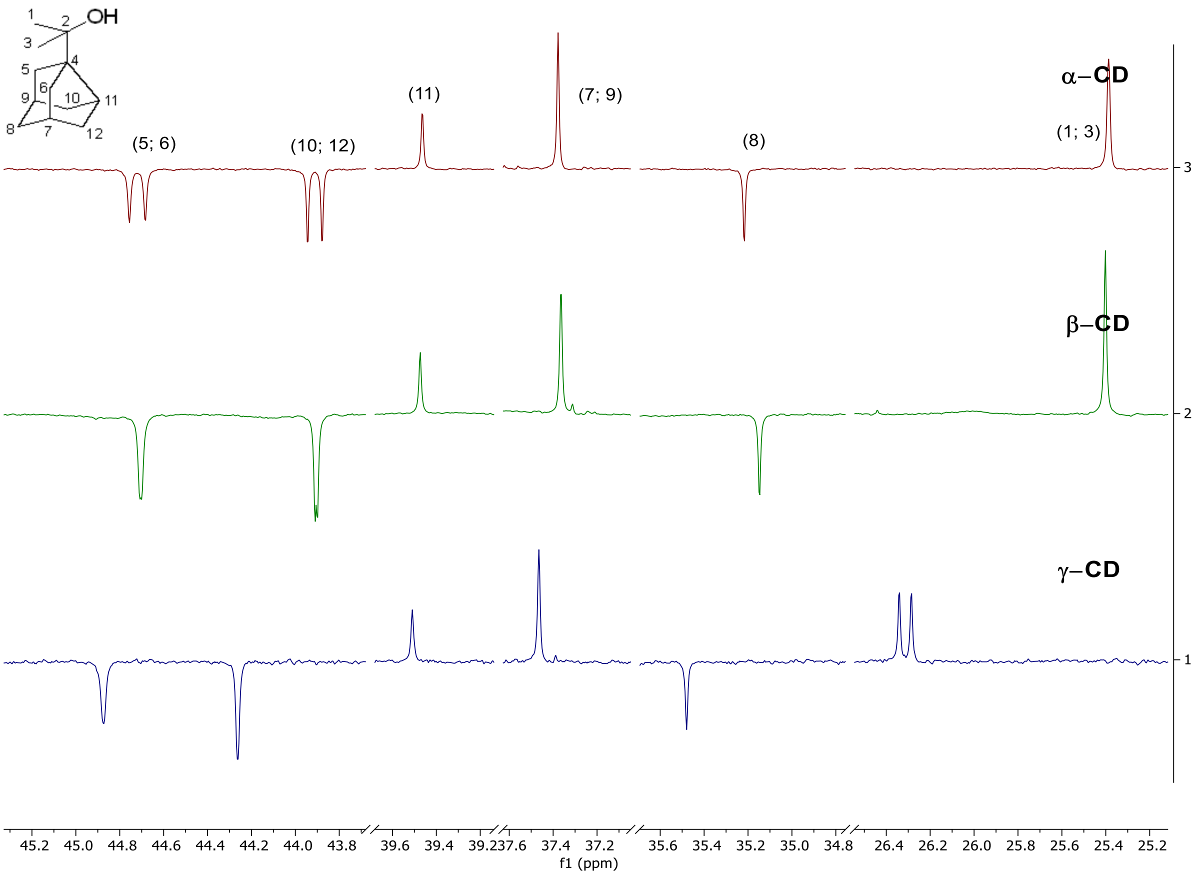

Figure S53 The partial ^{13}C DEPT spectra of **7** (10 mM) water solution in the presence of α-CD (20 mM); β-CD (10 mM); γ-CD (20 mM) (no buffer).

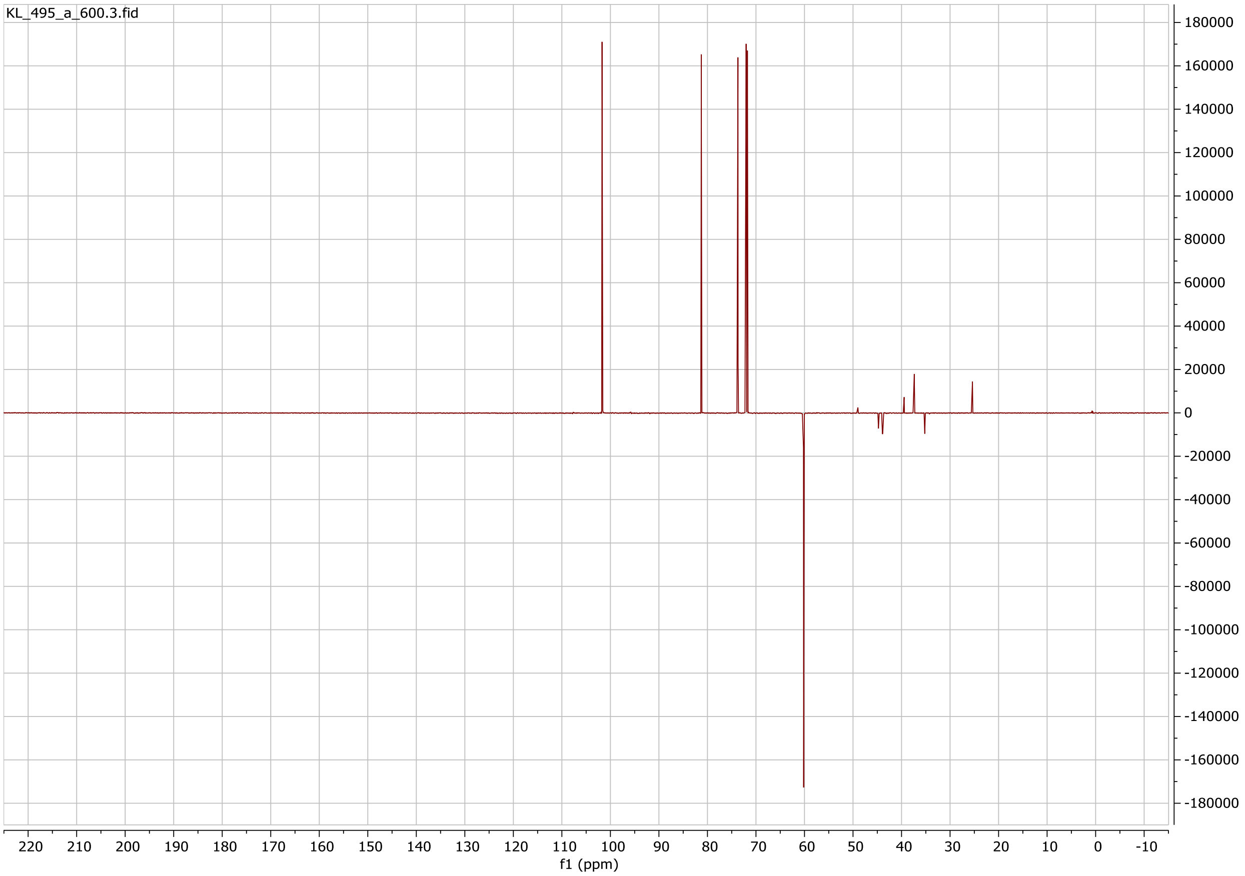

Figure S54 The ^{13}C DEPT spectra of **7** (10 mM) water solution in the presence of α-CD (20 mM) (no buffer).

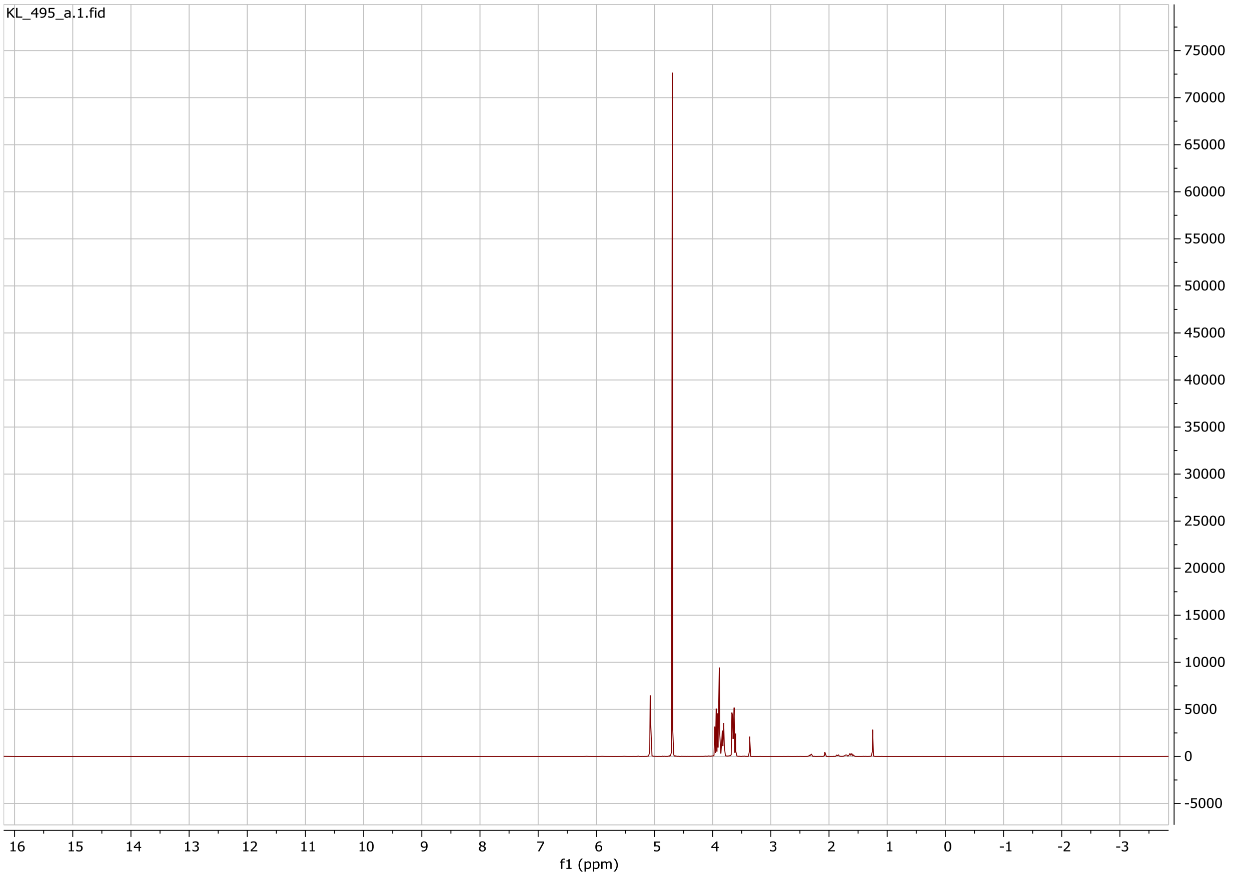

Figure S55 The ^{1}H NMR spectra of **7** (10 mM) water solution in the presence of α-CD (20 mM) (no buffer).

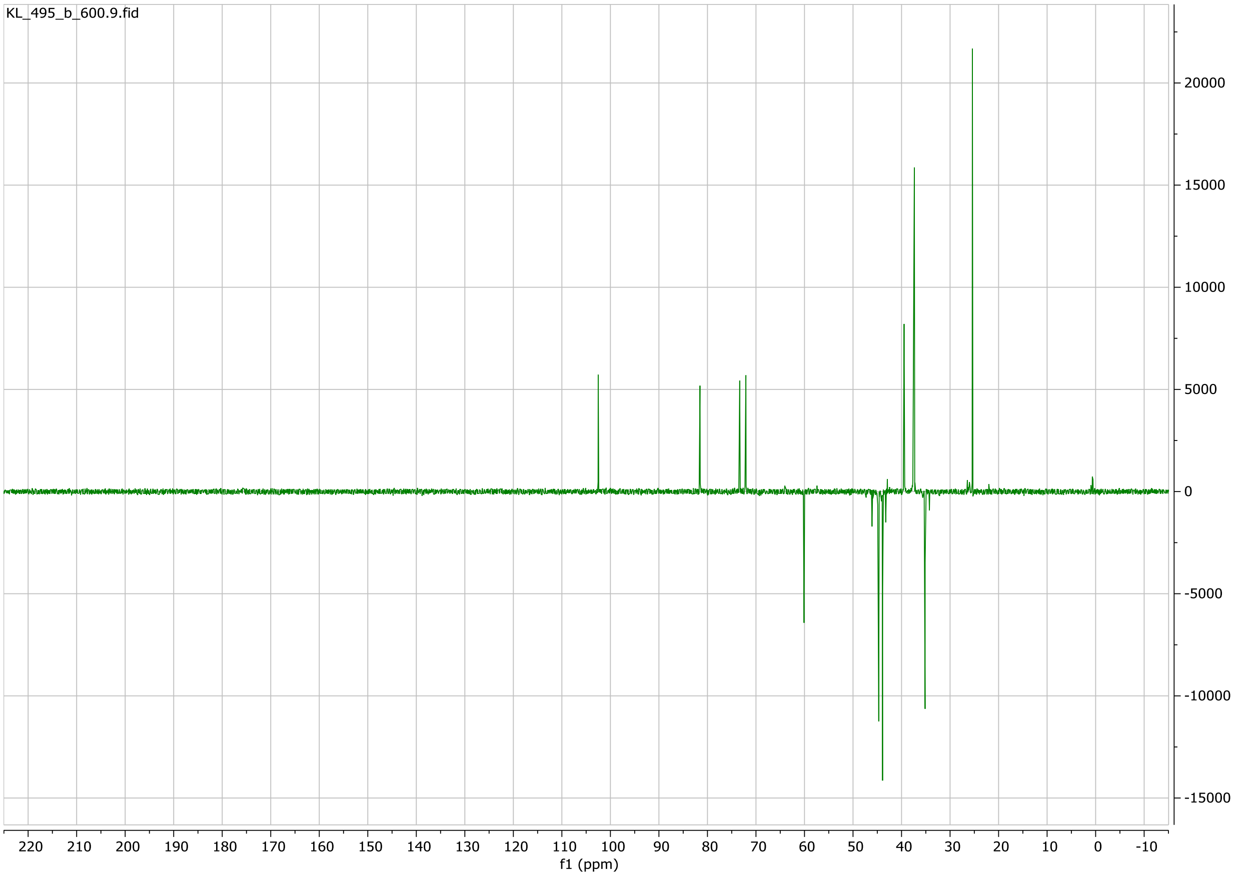

Figure S56 The ^{13}C DEPT spectra of **7** (10 mM) water solution in the presence of β-CD (10 mM) (no buffer).

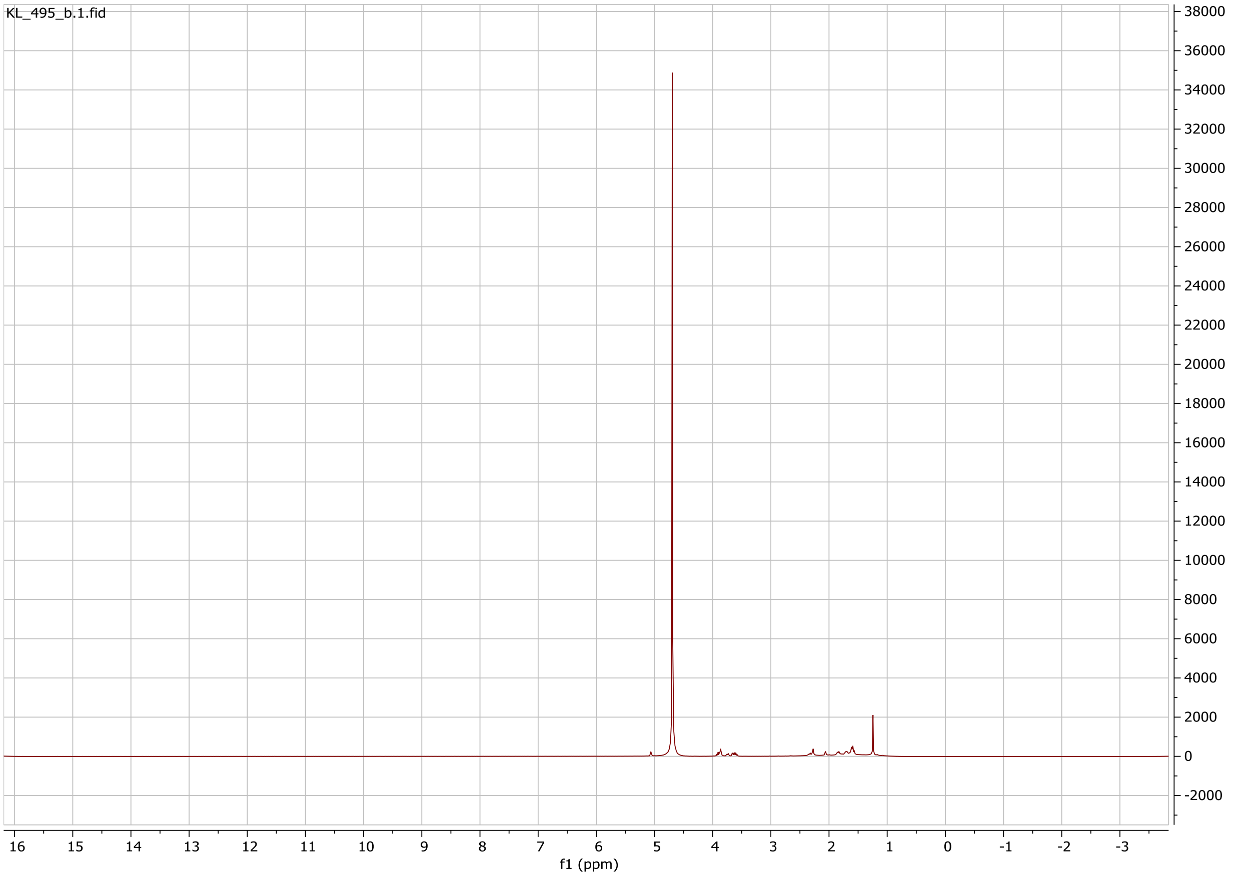

Figure S57 The ^{1}H NMR spectra of **7** (10 mM) water solution in the presence of β-CD (20 mM) (no buffer).

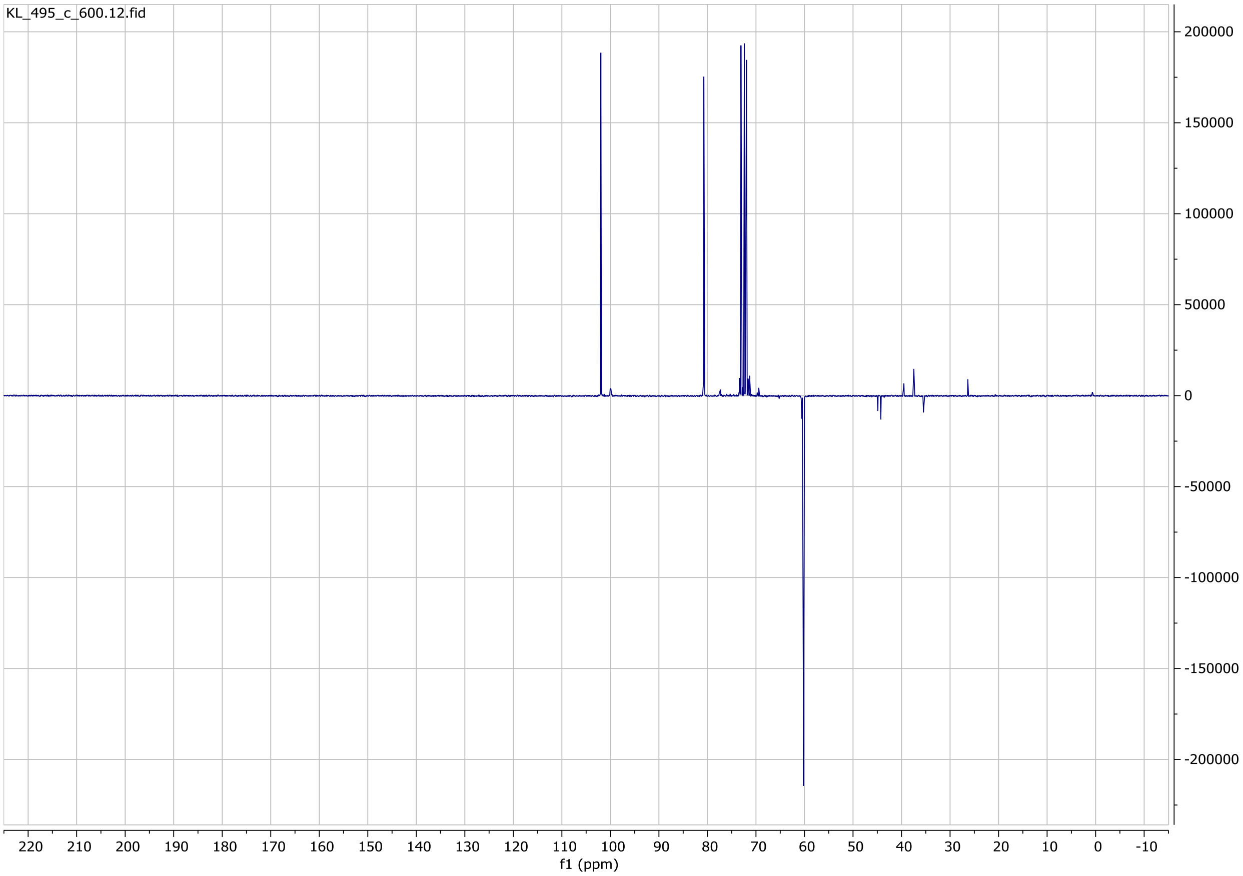

Figure S58 The ^{13}C DEPT spectra of **7** (10 mM) water solution in the presence of γ-CD (20 mM) (no buffer).

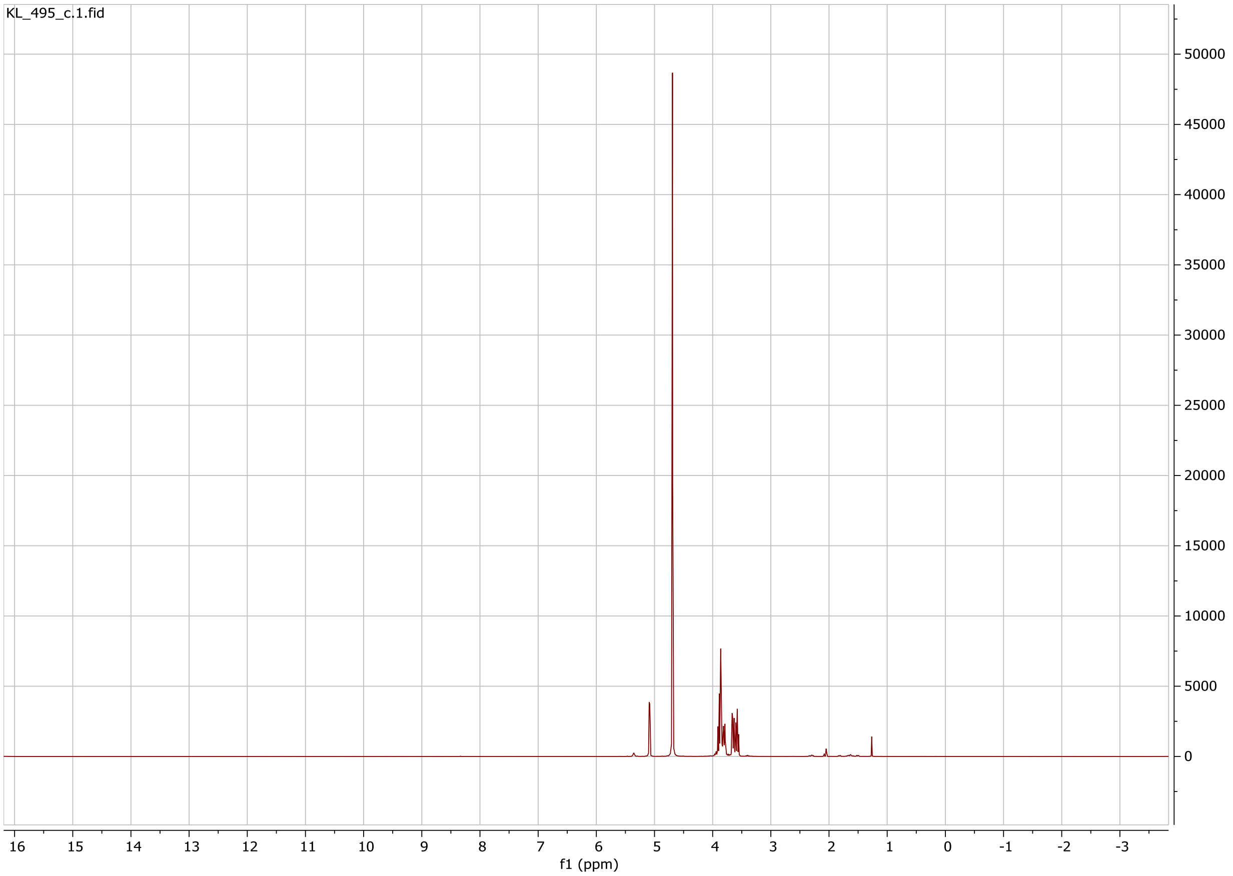

Figure S59 The ^{1}H NMR spectra of **7** (10 mM) water solution in the presence of γ-CD (20 mM) (no buffer).

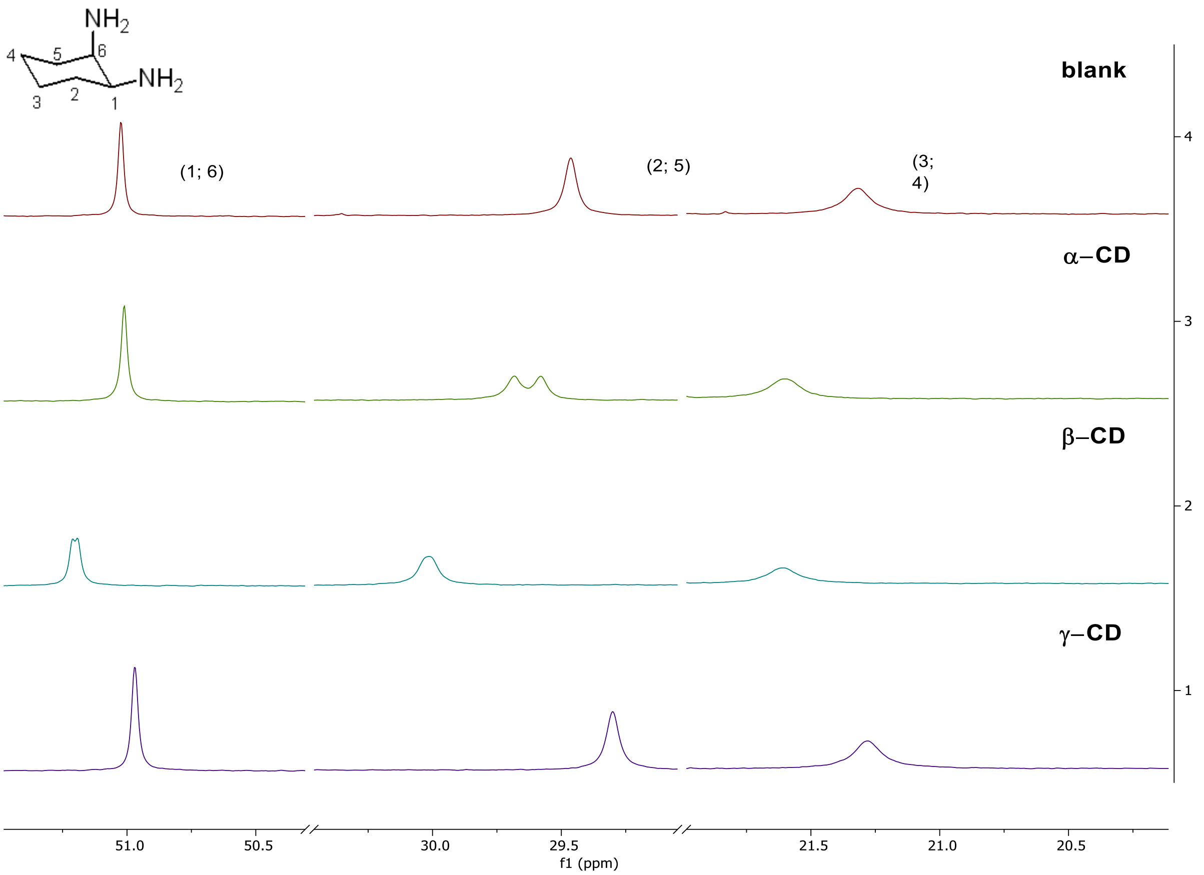

Figure S60 The partial ^{13}C NMR spectra of **8** (10 mM) water solution in the absence of any host (blank); in the presence of α-CD (20 mM); β-CD (10 mM); γ-CD (20 mM) (no buffer).

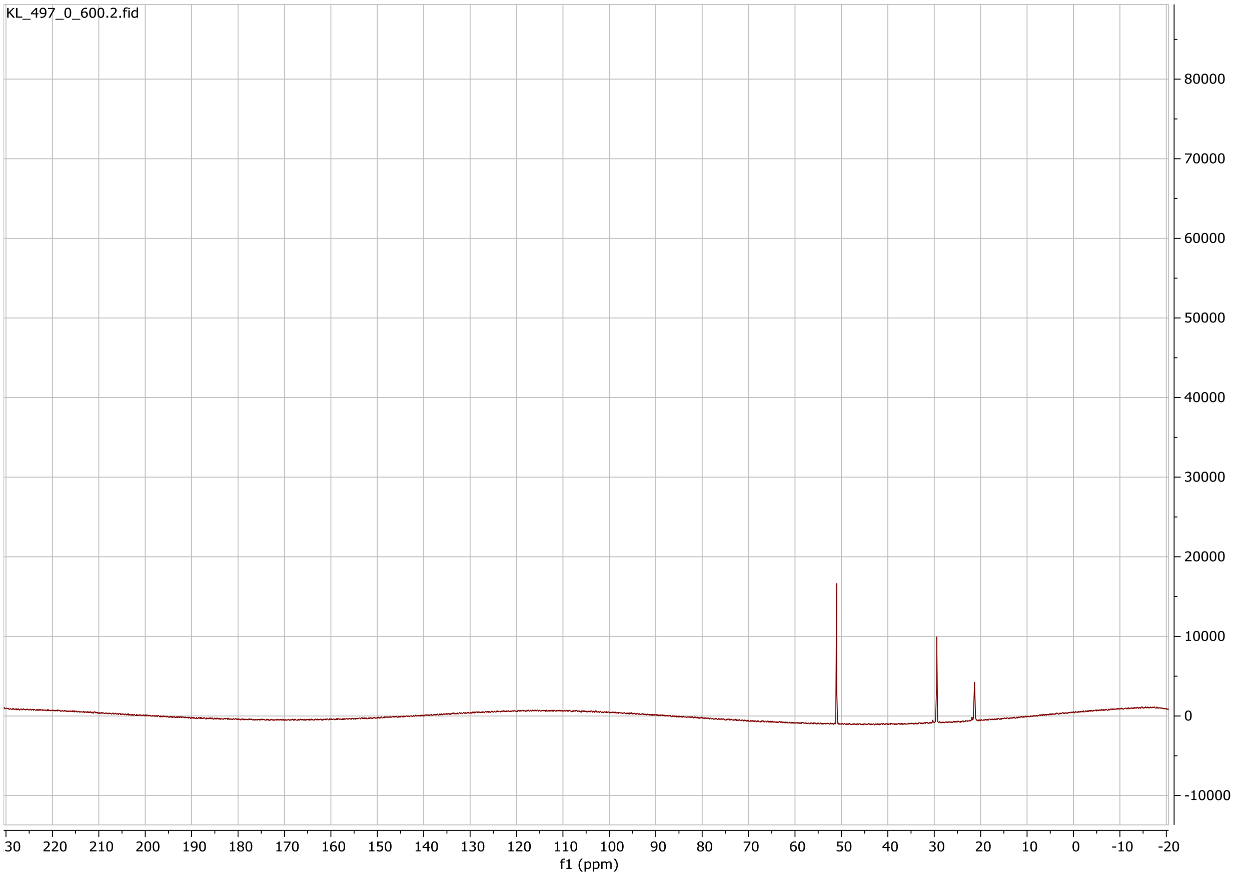

Figure S61 The ^{13}C NMR spectra of **8** (10 mM) water solution (no buffer).

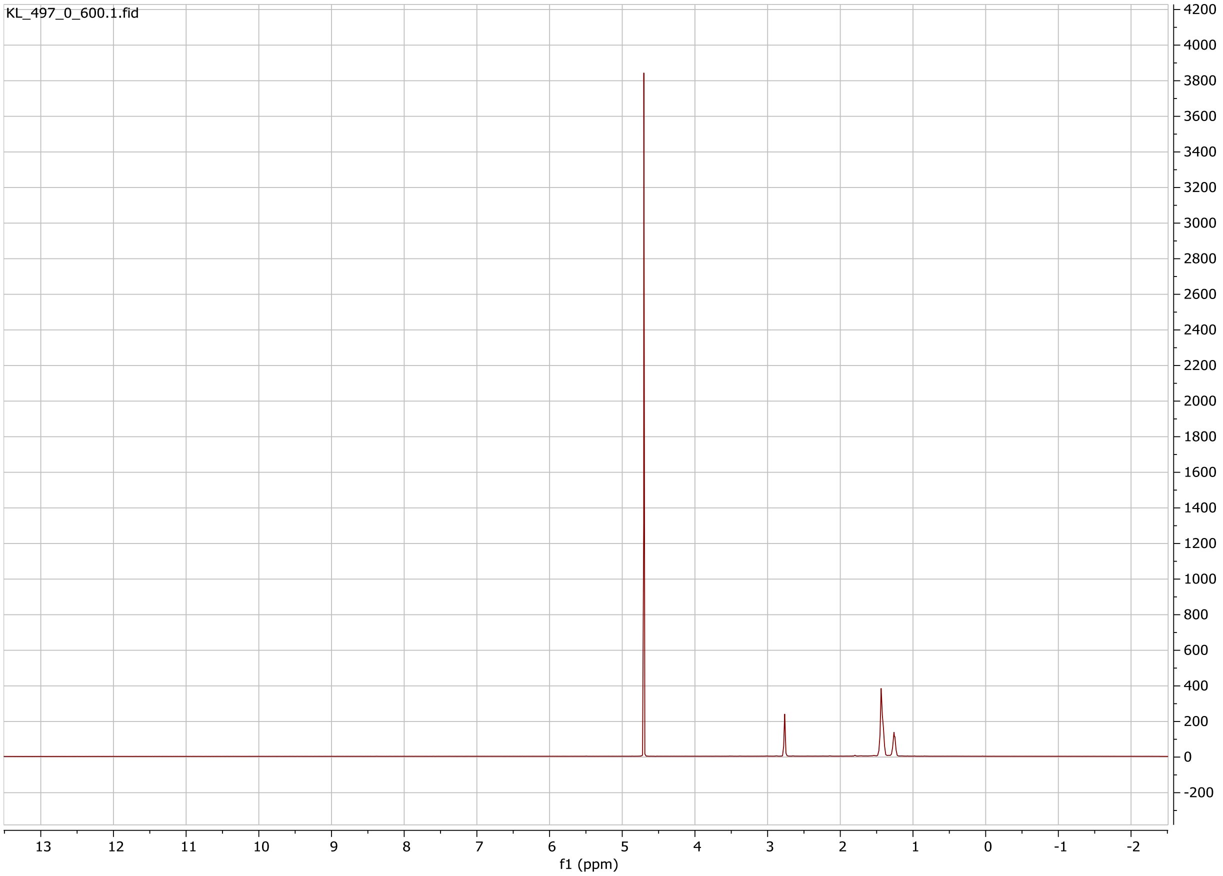

Figure S62 The ^{1}H NMR spectra of **8** (10 mM) water solution (no buffer).

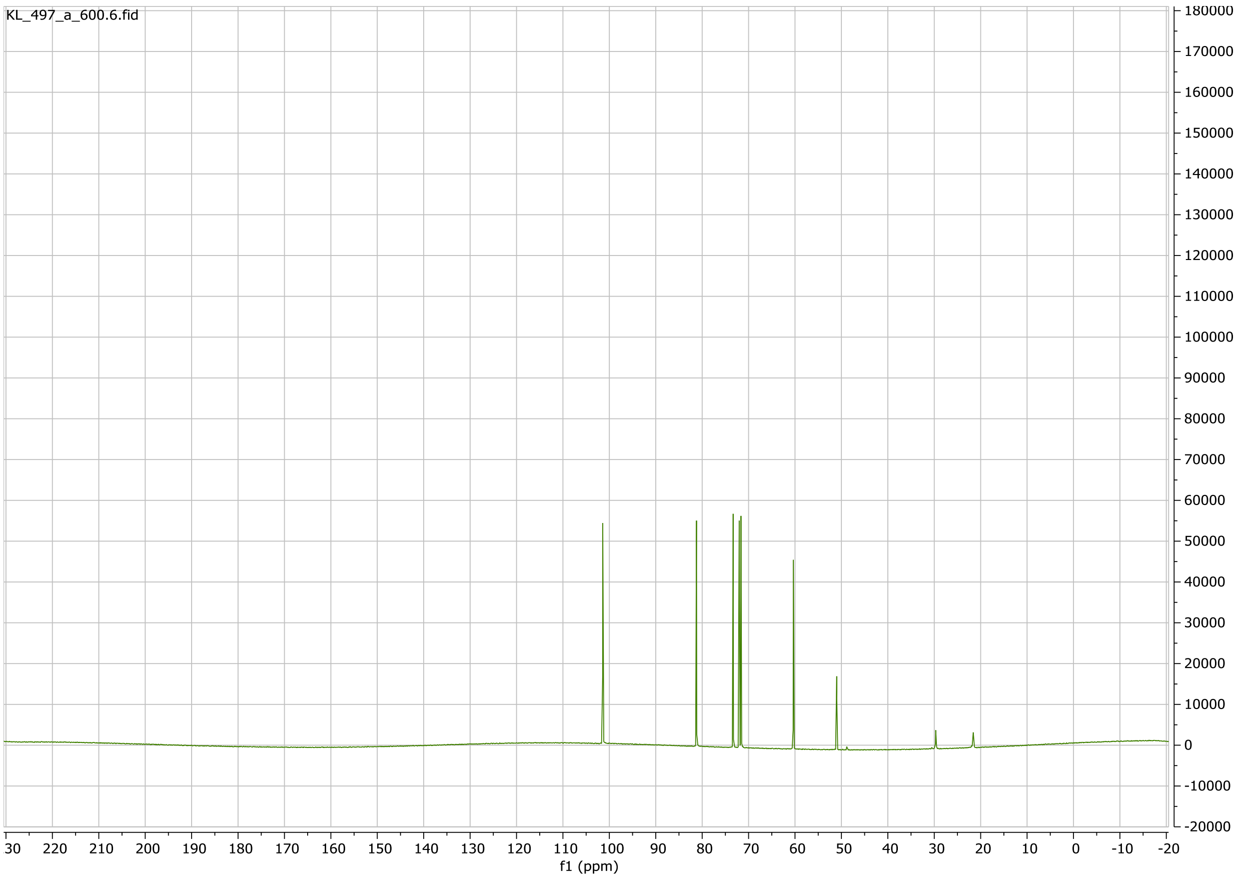

Figure S63 The ^{13}C NMR spectra of **8** (10 mM) in the presence of α-CD (20 mM) water solution (no buffer).

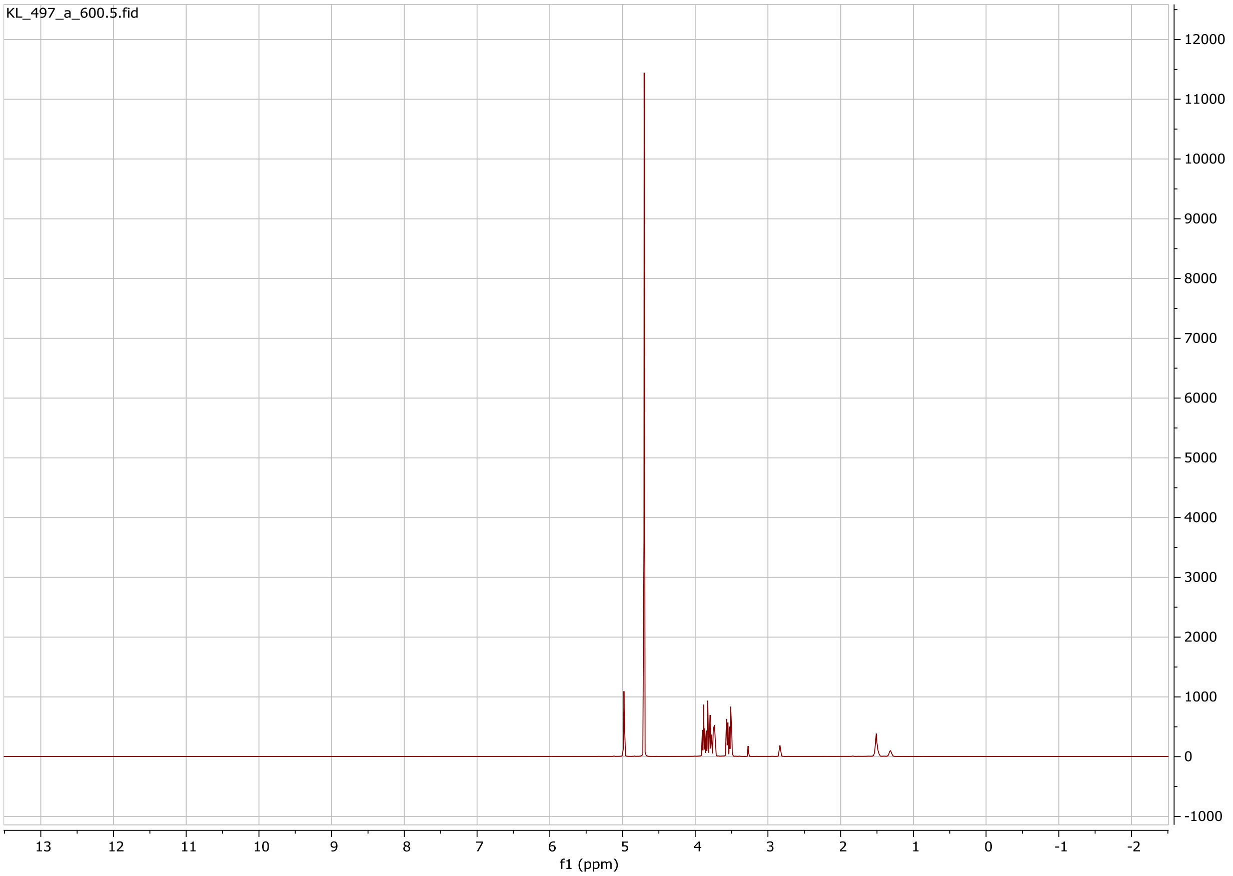

Figure S64 The ^{13}C NMR spectra of **8** (10 mM) in the presence of α-CD (20 mM) water solution (no buffer).

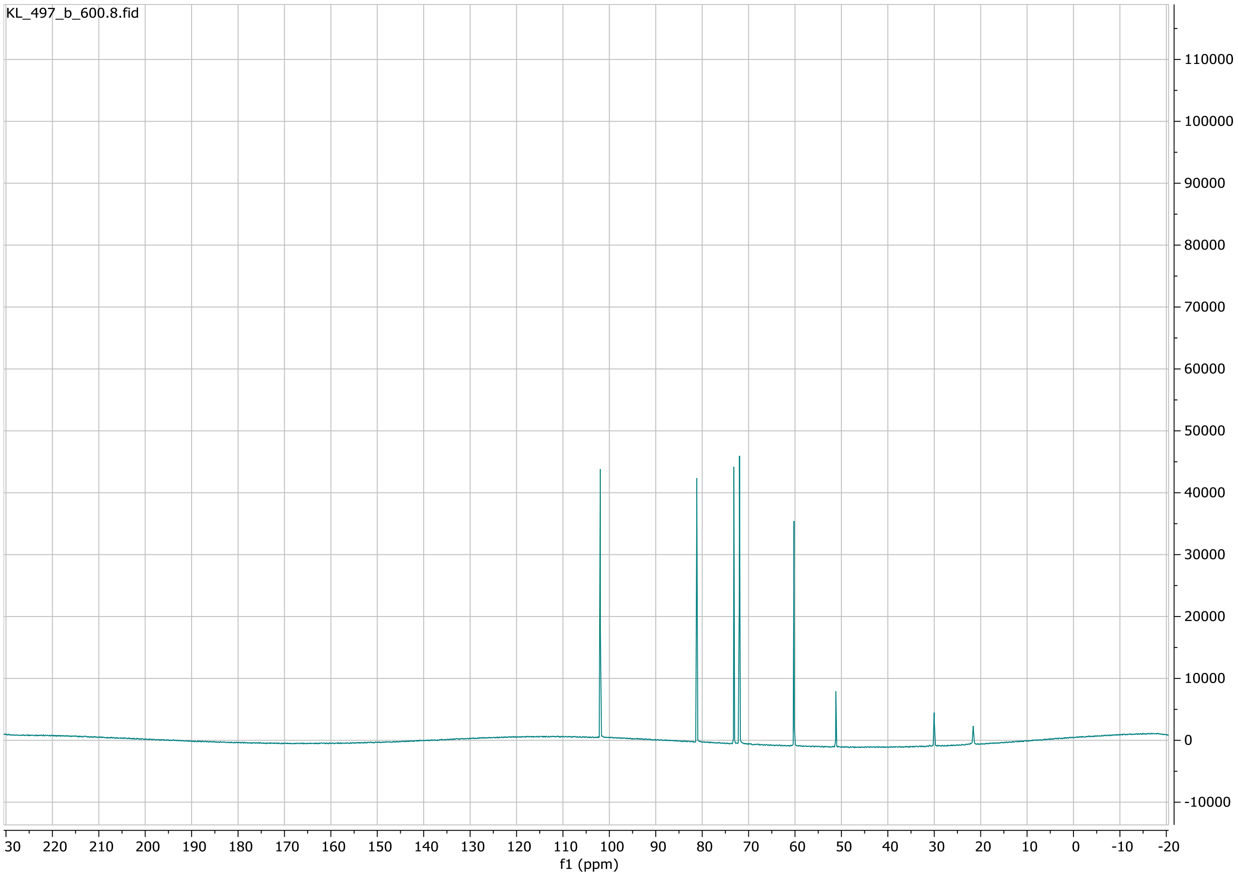

Figure S65 The ^{13}C NMR spectra of **8** (10 mM) in the presence of β-CD (10 mM) water solution (no buffer).

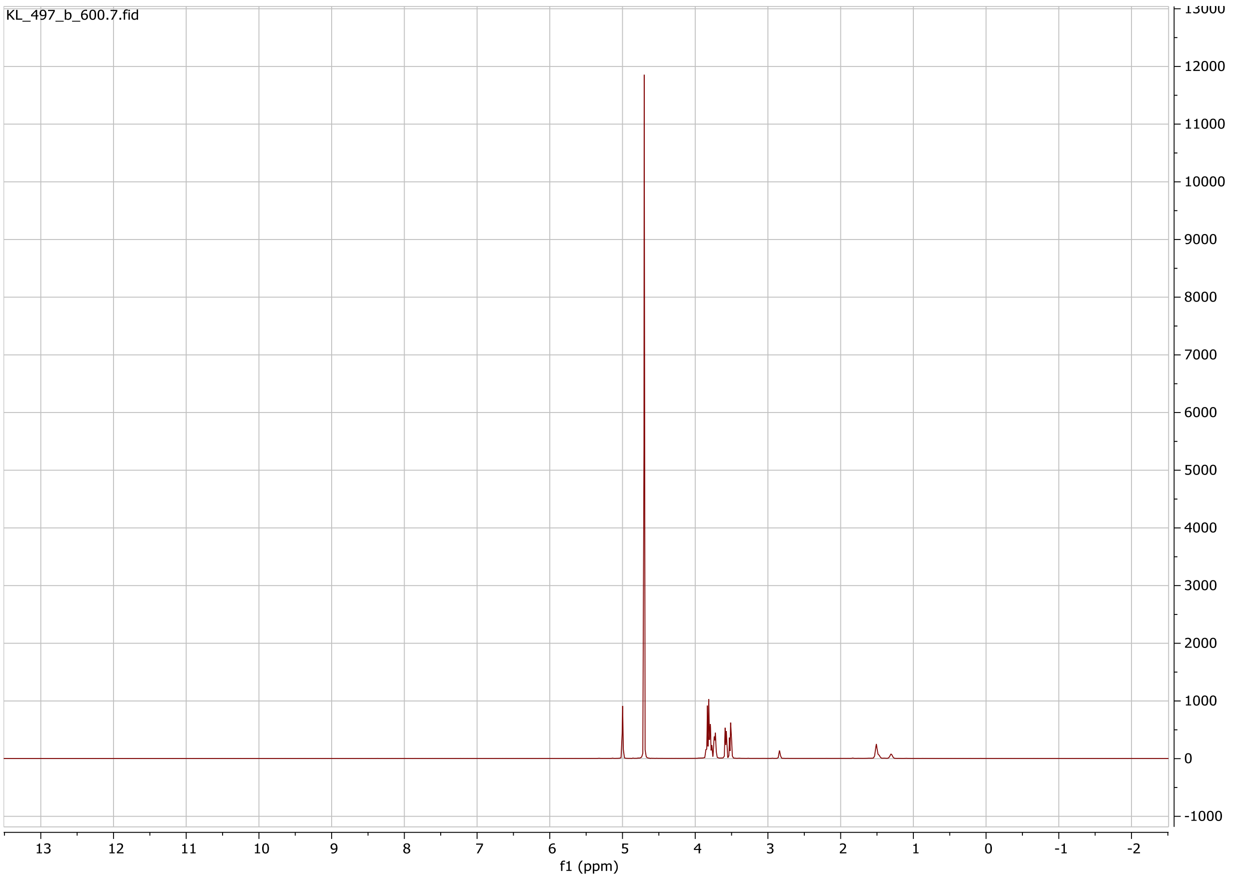

Figure S66 The ^{1}H NMR spectra of **8** (10 mM) in the presence of β-CD (10 mM) water solution (no buffer).

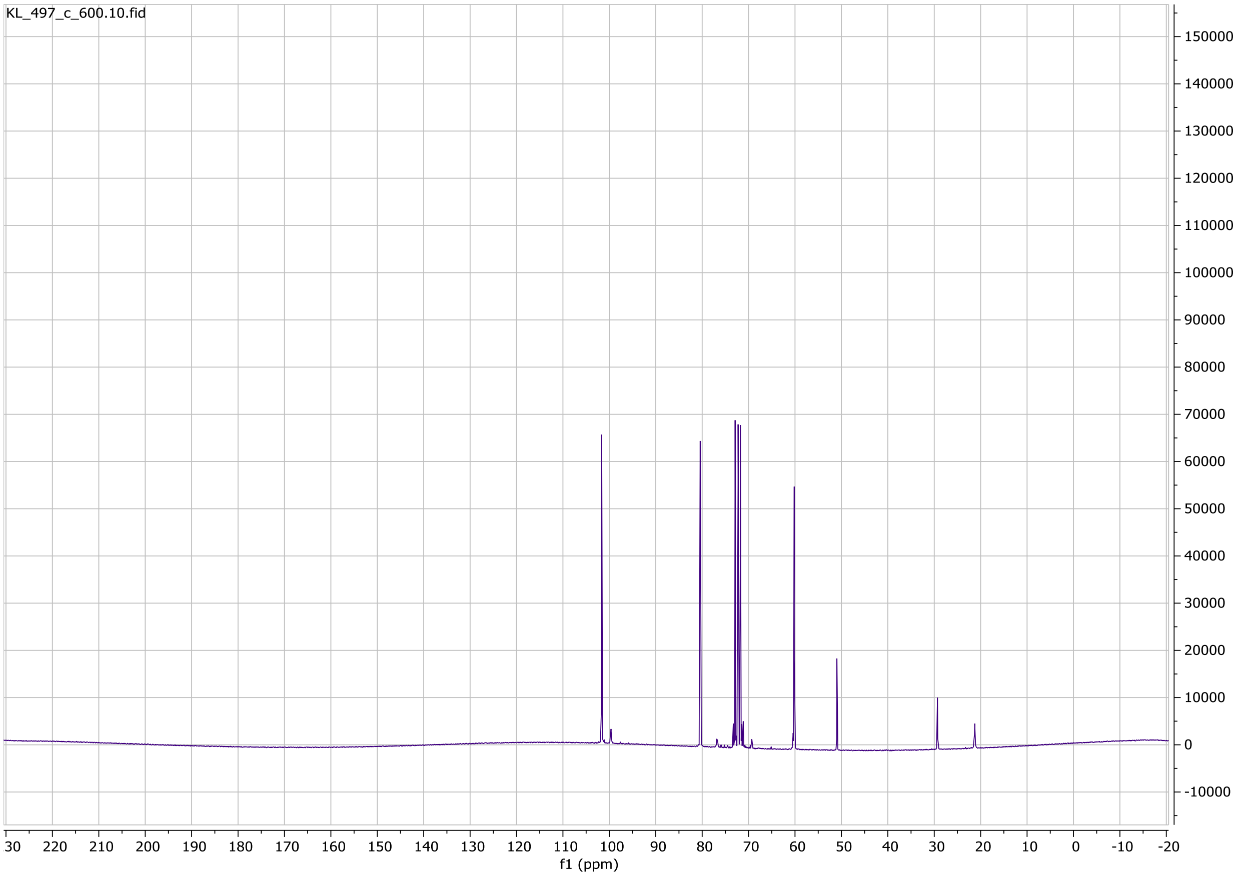

Figure S67 The ^{13}C NMR spectra of **8** (10 mM) in the presence of γ-CD (20 mM) water solution (no buffer).

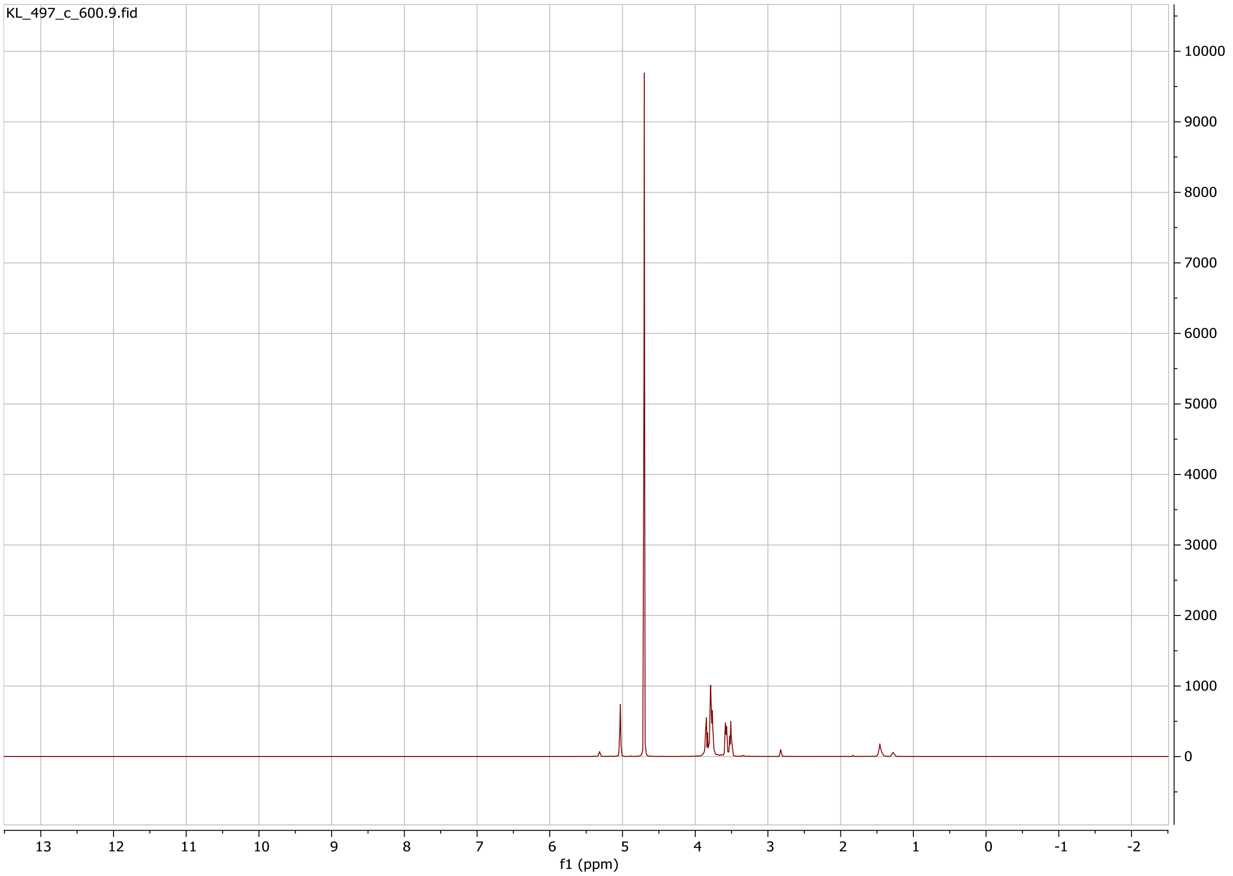

Figure S68 The ^1H NMR spectra of **8** (10 mM) in the presence of γ-CD (20 mM) water solution (no buffer).

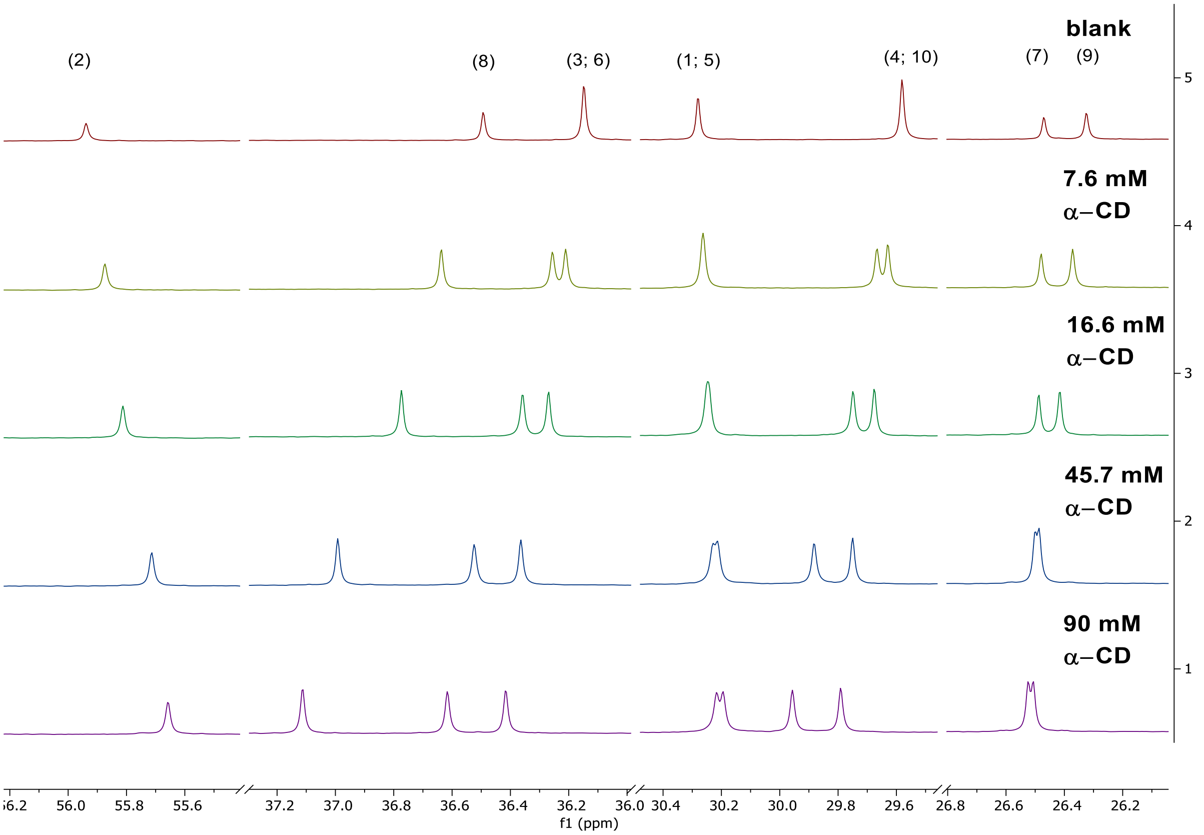

Figure S69 The partial ^{13}C NMR titration of 16 mM water solution of **1** with α-cyclodextrin (no buffer)

3. NMR study

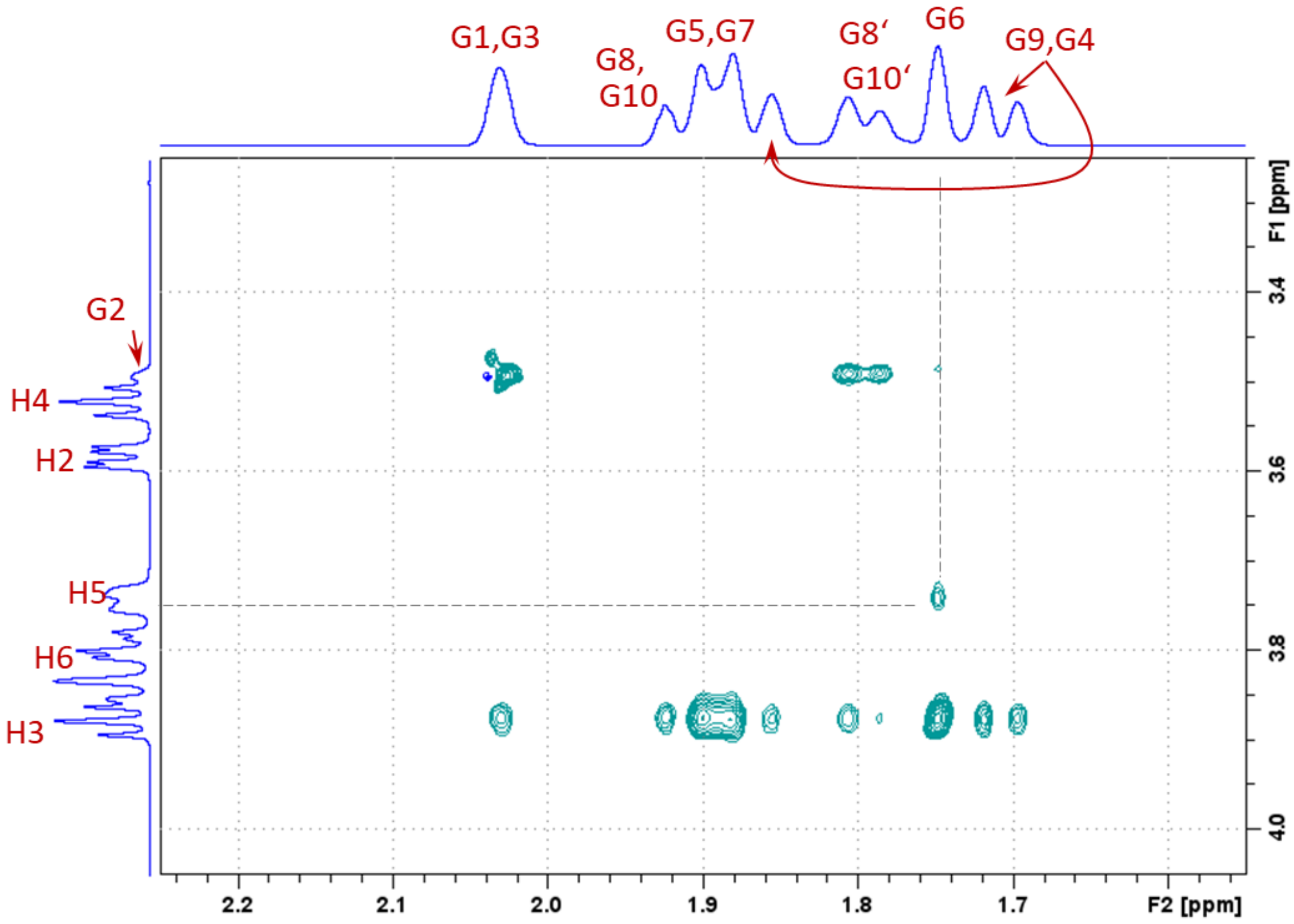

Figure S70 2D ROESY spectra (200 ms mixing) of 16 mM water solution α-cyclodextrin (host) with 16mg of 2-aminoadamantane (guest), no buffer. The highlighted crosspeak indicate deep penetration of the guest into the cavity.

4. Computational study

The initial structure of alpha-CD was obtained from the crystal structure 4FEM [1] and parametrized using the CHARMM force field [2]. Initial structures of compounds 1-8 were created using the Molefacture tool from the VMD software package [3] and force constants were generated using the ParamChem web server [4].

Molecular dynamics simulations of TIP3P water-enveloped host-guest complexes were performed using the NAMD software package [5,6]. The solvated complexes were energy minimized for 2000 steps. Newton's equations of motion were integrated using the Verlet algorithm with a step of 2 fs. The simulated systems were heated up to 280 K and properly equilibrated. Then production MD simulations (10 × 100ns for each compound) were run at 280 K. The temperature was controlled by a Langevin thermostat with a damping coefficient of 5/ps. The Langevin piston method was applied with an oscillation period of 100 fs, decay – damping time scale of 50 fs was used to maintain a constant pressure of 1 atm. Short-range van der Waals interactions were calculated using a switching function of 10.0 Å, with a cutoff of 12.0 Å. Long-range electrostatic interactions were calculated using the Particle Mesh Ewald method with a cut-off of 12.0 Å.

The spatial 3D densities of prochiral atom occurrences were determined using the CPPTRAJ software package [7]. The software packages VMD [MD3] and UCSF Chimera [8] were used to visualize the simulated systems and spatial 3D densities.

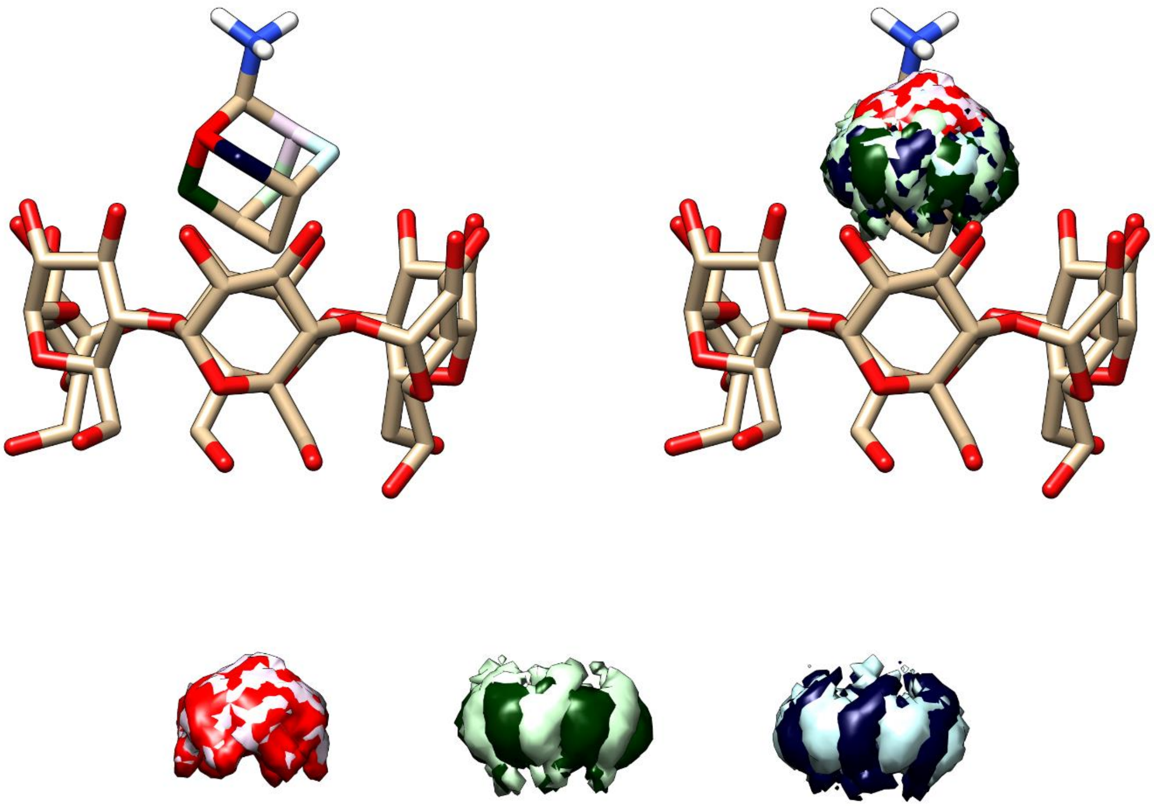

Figure S71 MD1 Molecular model of the host (α-CD) - guest (compound 1) complex. The 3D densities show the spatial distribution of prochiral atoms within MD simulations.

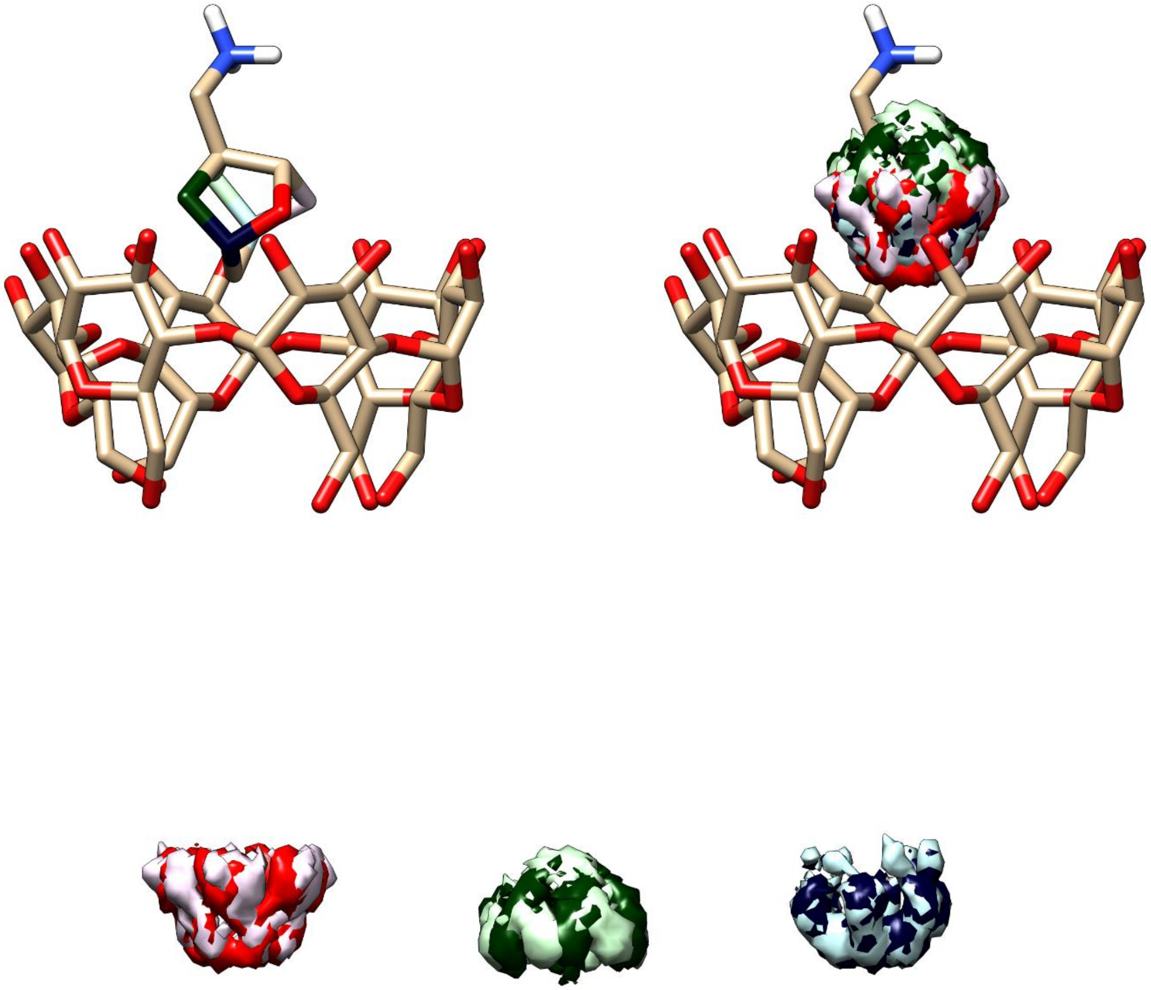

Figure S72 MD2 Molecular model of the host (α-CD) - guest (compound 2) complex. The 3D densities show the spatial distribution of prochiral atoms within MD simulations.

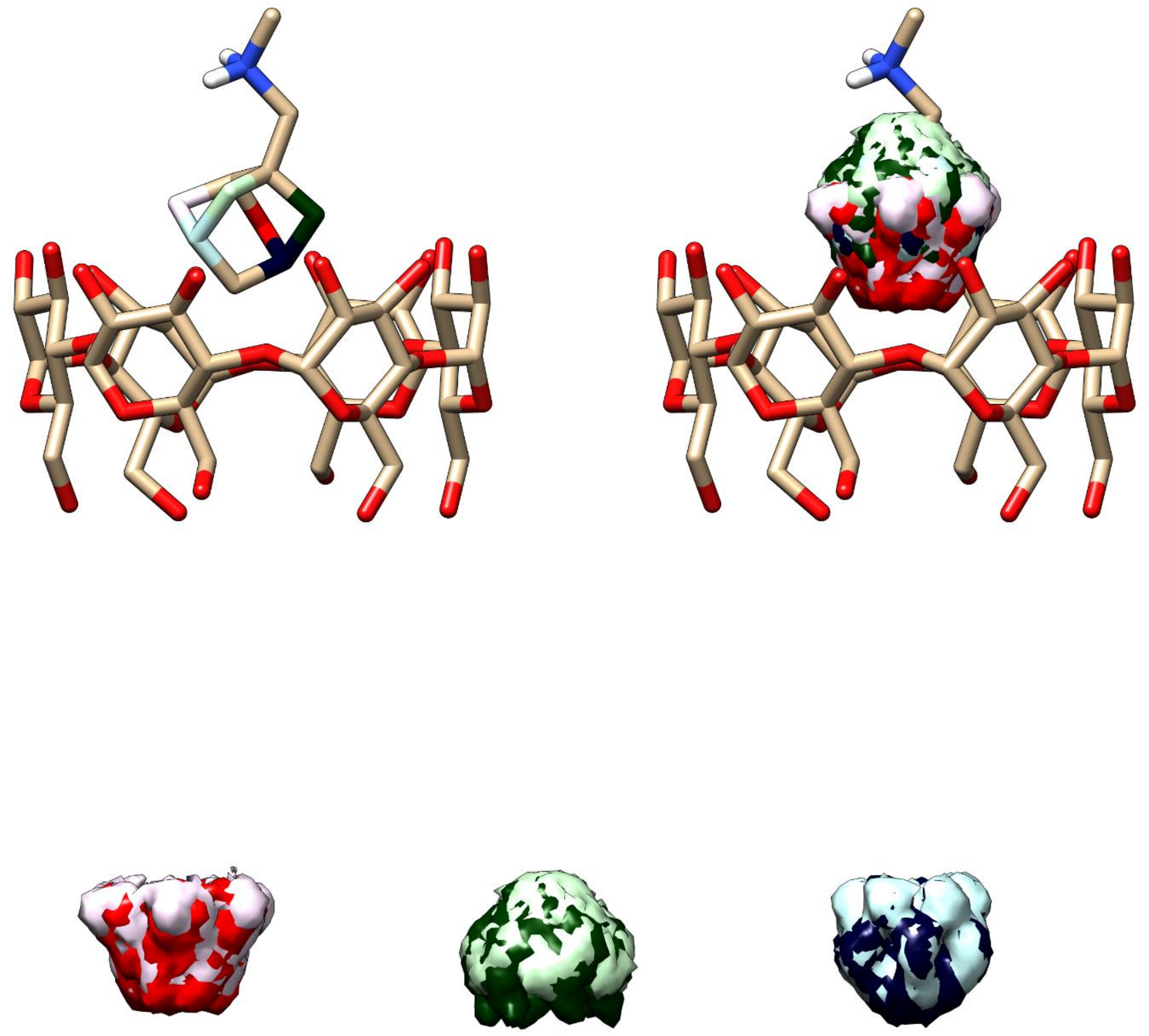

Figure S73 MD3 Molecular model of the host (α-CD) - guest (compound 3) complex. The 3D densities show the spatial distribution of prochiral atoms within MD simulations.

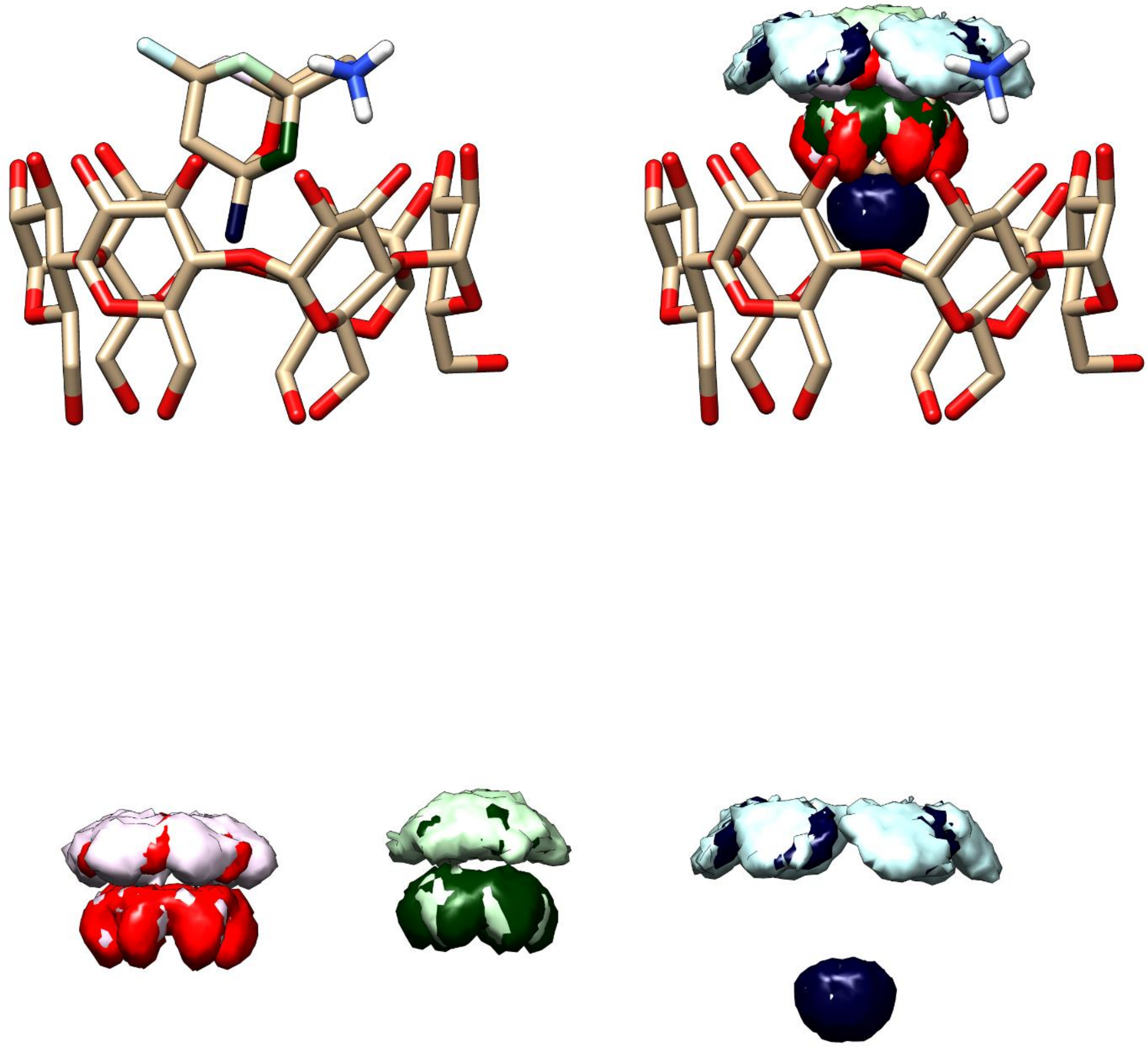

Figure S 74 MD4 Molecular model of the host (α-CD) - guest (compound 4) complex. The 3D densities show the spatial distribution of prochiral atoms within MD simulations.

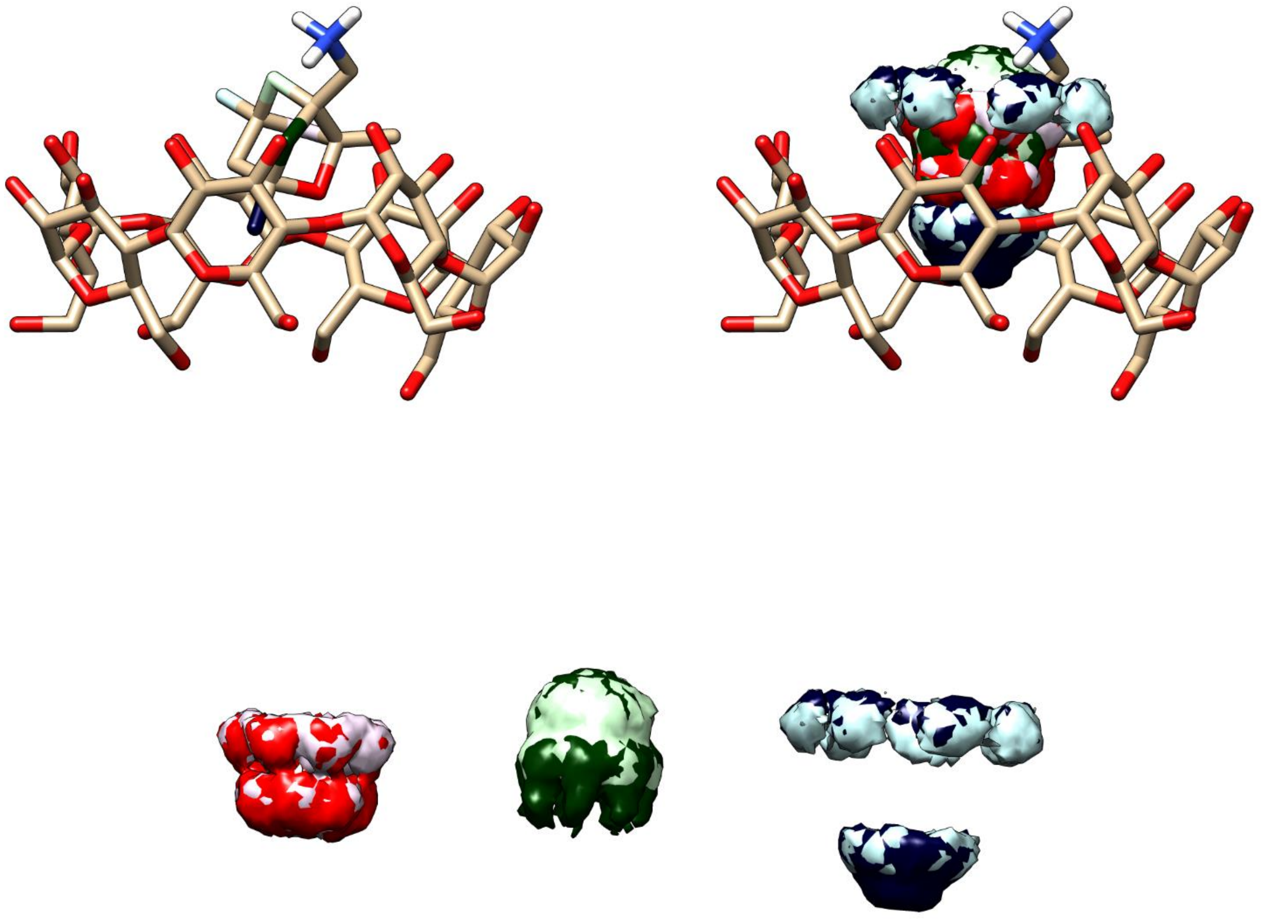

Figure S75 MD5 Molecular model of the host (β-CD) - guest (compound 4) complex. The 3D densities show the spatial distribution of prochiral atoms within MD simulations.

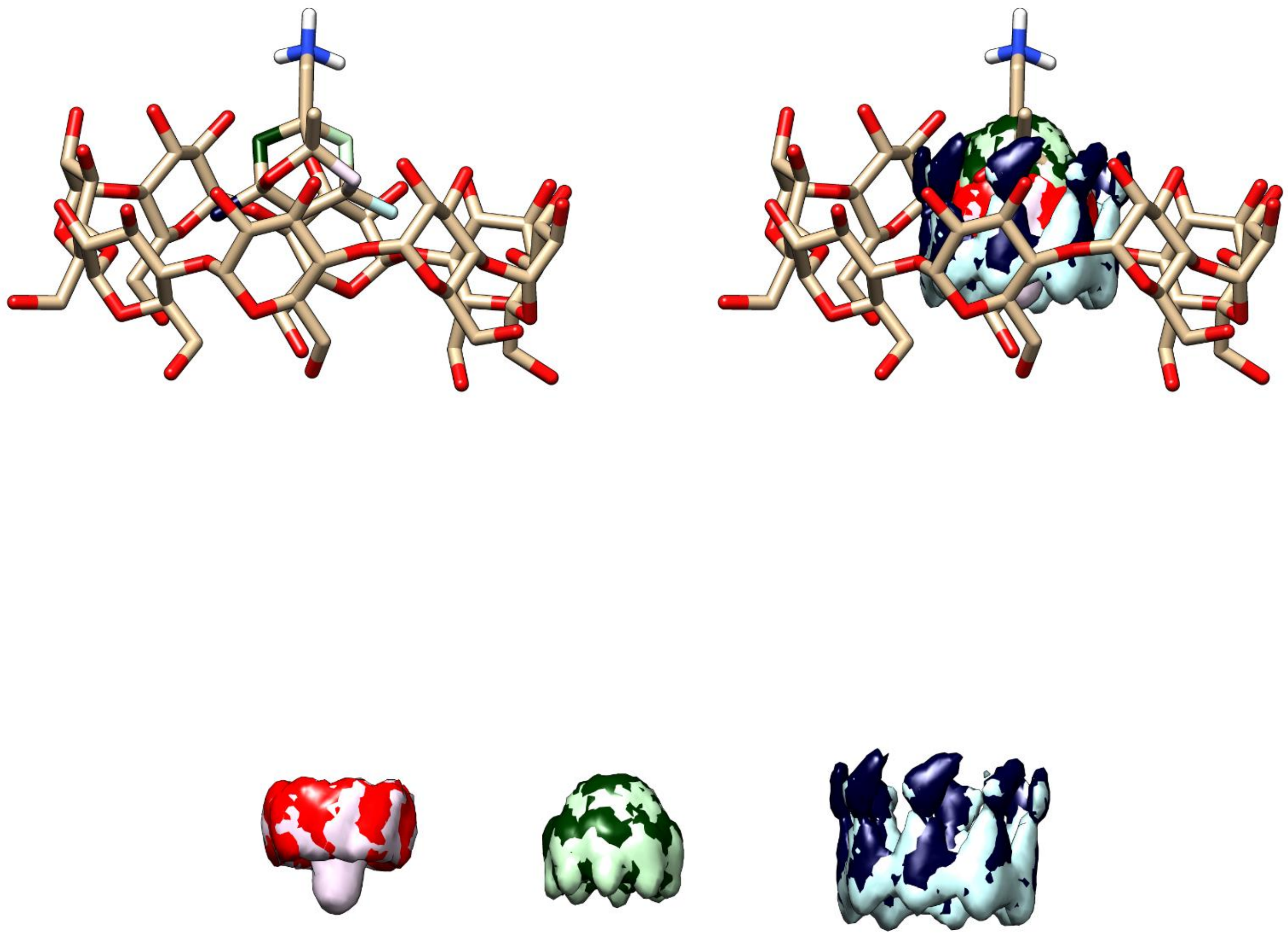

Figure S76 MD6 Molecular model of the host (γ -CD) - guest (compound 4) complex. The 3D densities show the spatial distribution of prochiral atoms within MD simulations.

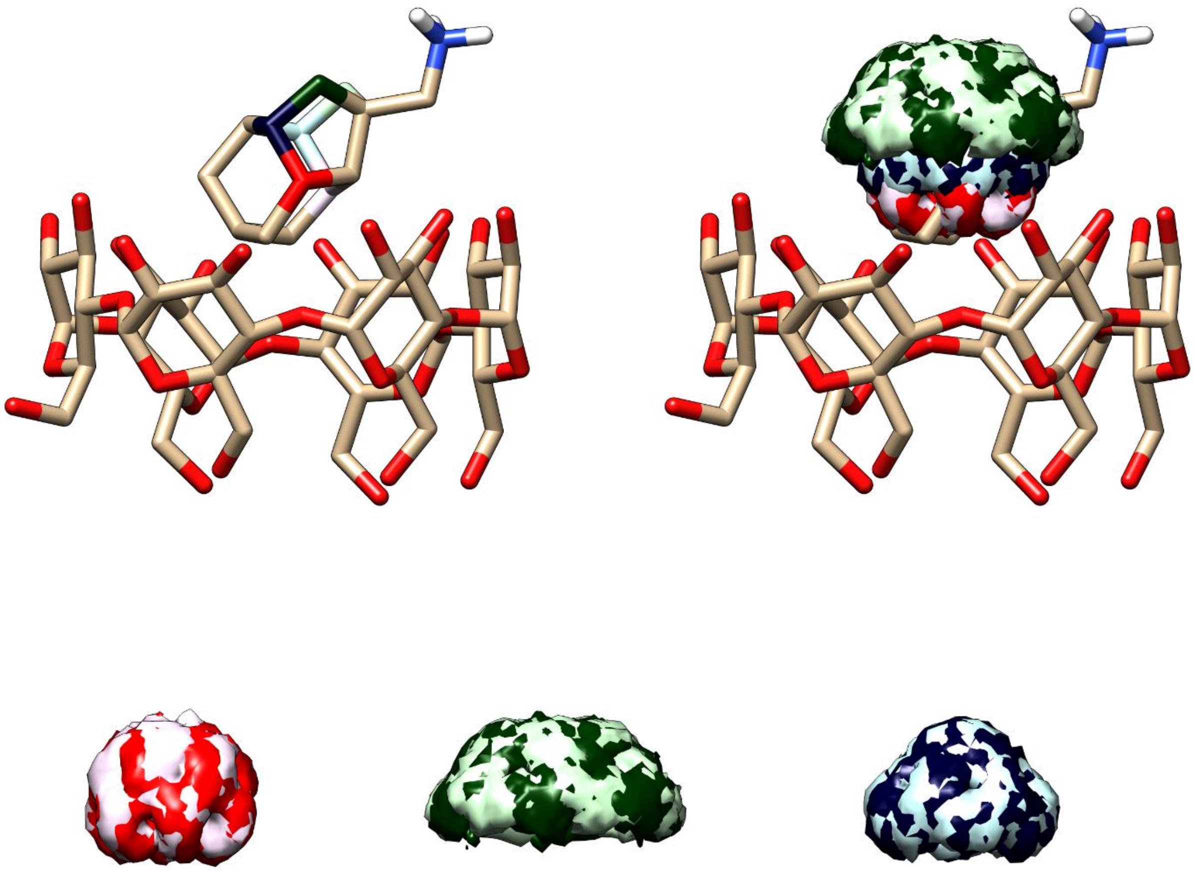

Figure S77 MD7 Molecular model of the host (α-CD) - guest (compound 5) complex. The 3D densities show the spatial distribution of prochiral atoms within MD simulations.

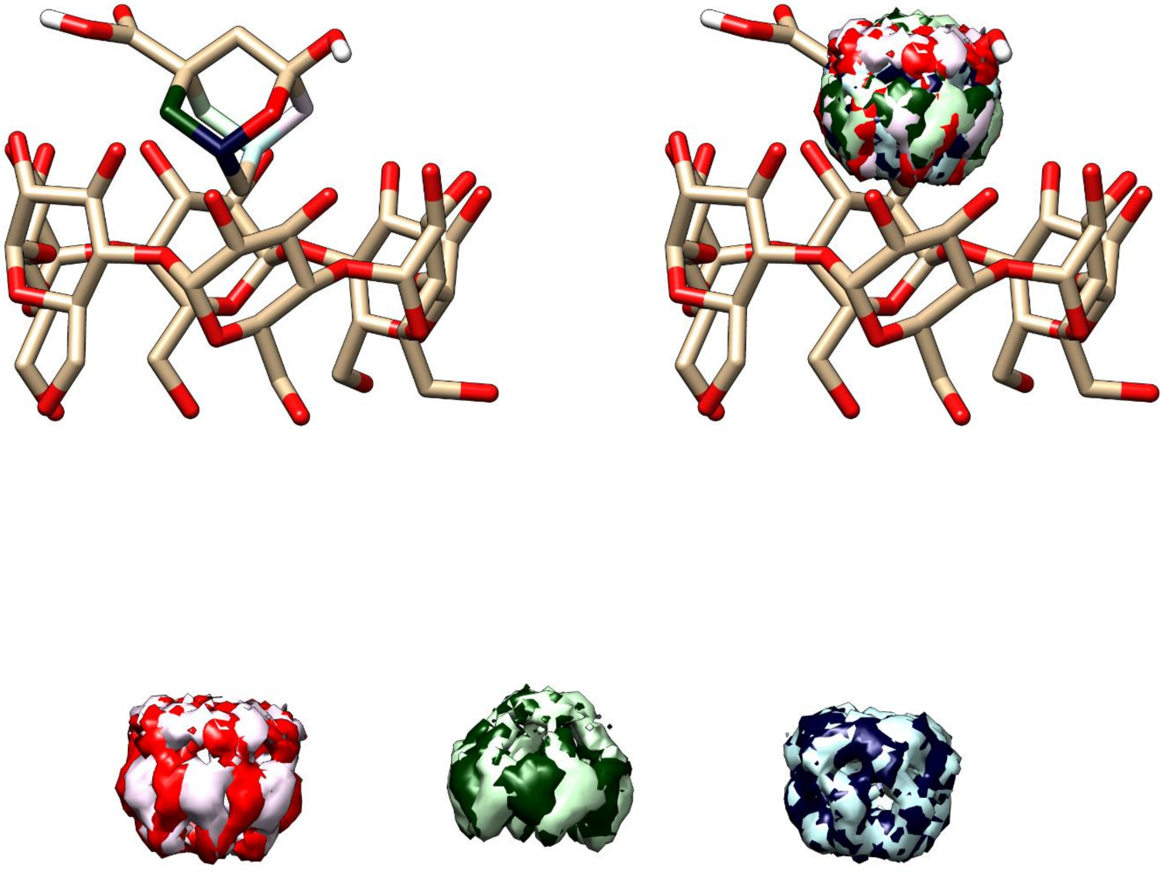

Figure S78 MD8 Molecular model of the host (α-CD) - guest (compound 6) complex. The 3D densities show the spatial distribution of prochiral atoms within MD simulations.

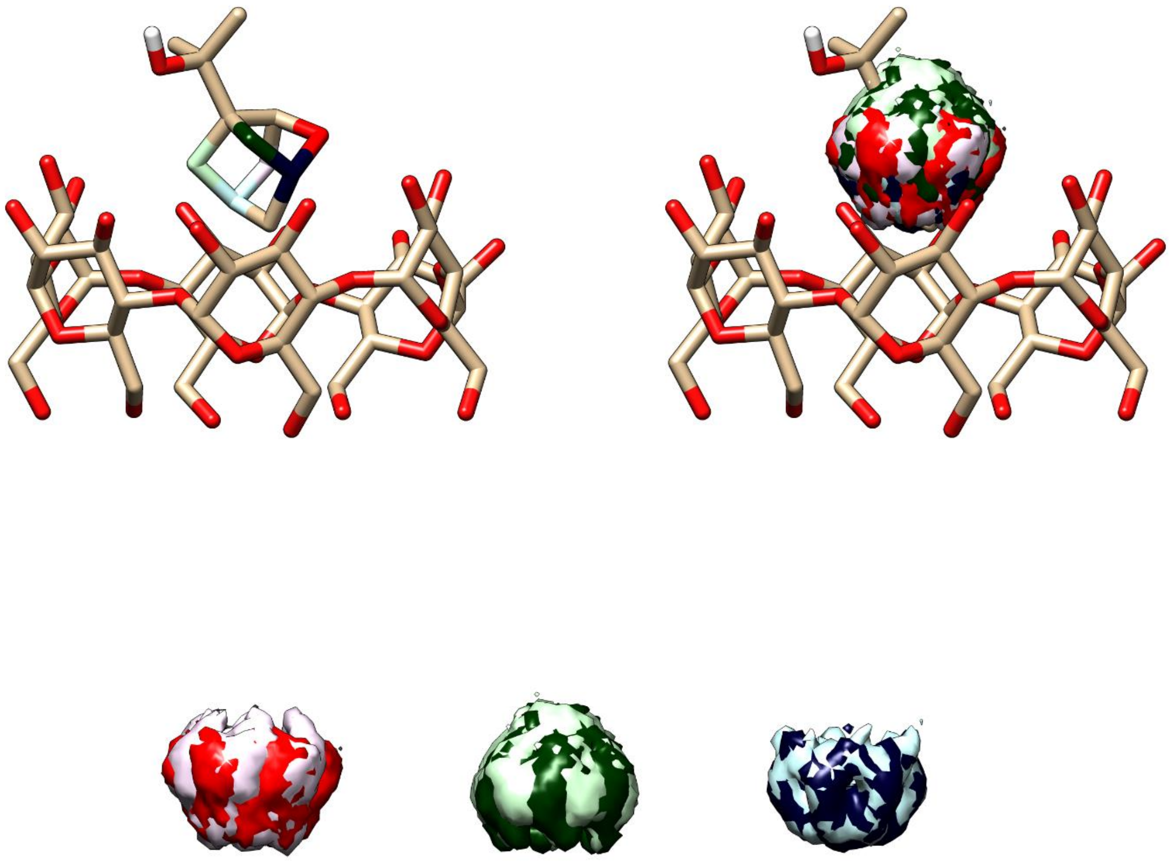

Figure S79 MD9 Molecular model of the host (α-CD) - guest (compound 7) complex. The 3D densities show the spatial distribution of prochiral atoms within MD simulations.

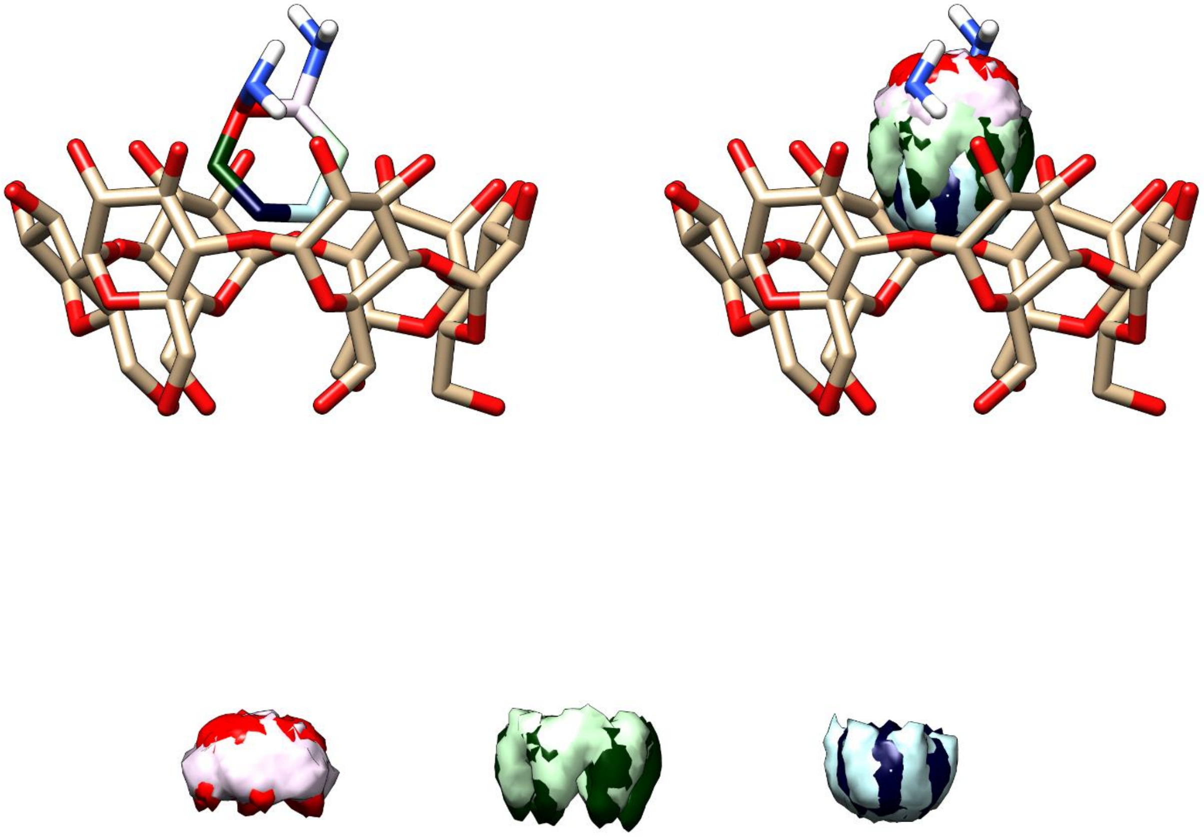

Figure S80 MD10 Molecular model of the host (α-CD) - guest (compound 8) complex. The 3D densities show the spatial distribution of prochiral atoms within MD simulations.

5. Crystallographic data collection and refinement details

The x-ray experiment was carried on diffractometer Bruker D8 VENTURE Kappa Duo PHOTONIII by IμS micro-focus sealed tube Cu Kα (λ= 1.54178 Å) at a temperature of 150(2) K. The structure was solved by direct methods (XT [9]) and refined by full matrix least squares based on F^2 (SHELXL2019 [10]). The hydrogen atoms on carbon were fixed into idealized positions (riding model) and assigned temperature factors $H_{iso}(H) = 1.2\ U_{eq}$(pivot atom). Some hydrogen atom in -OH moieties were found on difference Fourier map and were included in the refinement to partially elucinate the formation of the dimer. The absolute structure of the crystal was assigned, based on known chirality of cyclodextrine moiety.

The structure determination was severely affected by vast disorders. The several -CH2-OH moieties on outer rings of dimer are disordered as well as surrounding pool of water molecules. The contribution of all water molecules (except one) was removed from diffraction pattern using Squeeze procerure of Platon[40] softvare. The electron density inside of cavity of the dimer is witnessing the presense of noradamentane molecule, however due its disorder the restrictions on its geometry and displacement parameters have to be applied during refinement.

Crystal data for **rh_no7**: $C_{36}H_{56}O_{30}\cdot C_{36}H_{54}O_{30}\cdot C_{10}H_{17}N\cdot O$, M_r = 2102.84; Orthorhombic , $P\ 2_1\ 2_1\ 2_1$, (No 19), a = 13.9590 (4) Å, b = 24.5261 (7) Å, c = 30.9239 (9) Å ,V = 10587.1 (5) Å^3, Z = 4, D_x = 1.319 Mg m^{-3}, Needle, colourless of dimensions 0.50 × 0.07 × 0.03 mm, multi-scan absorption correction (μ = 0.99 mm^{-1}) T_{min} = 0.661, T_{max} = 0.752; a total of 131882 measured reflections (θ_{max} = 67°), from which 18708 were unique (R_{int} = 0.084) and 15958 observed according to the $I > 2\sigma(I)$ criterion. The refinement converged (Δ/σ_{max}= 0.001) to R = 0.071 for observed reflections and $wR(F^2)$ = 0.188, GOF = 1.02 for 1244 parameters and all 18708 reflections. The final difference map displayed peaks ($\Delta\rho_{max}$ = 0.74, $\Delta\rho_{min}$ -1.01 e.Å^{-3}). Absolute structure parameter: 0.29 (6) (4) [11].

X-ray crystallographic data have been deposited with the Cambridge Crystallographic Data Centre (CCDC) under deposition number 2302954 for **rh_no7** and can be obtained free of charge from the Centre via its website (https://www.ccdc.cam.ac.uk/structures/).

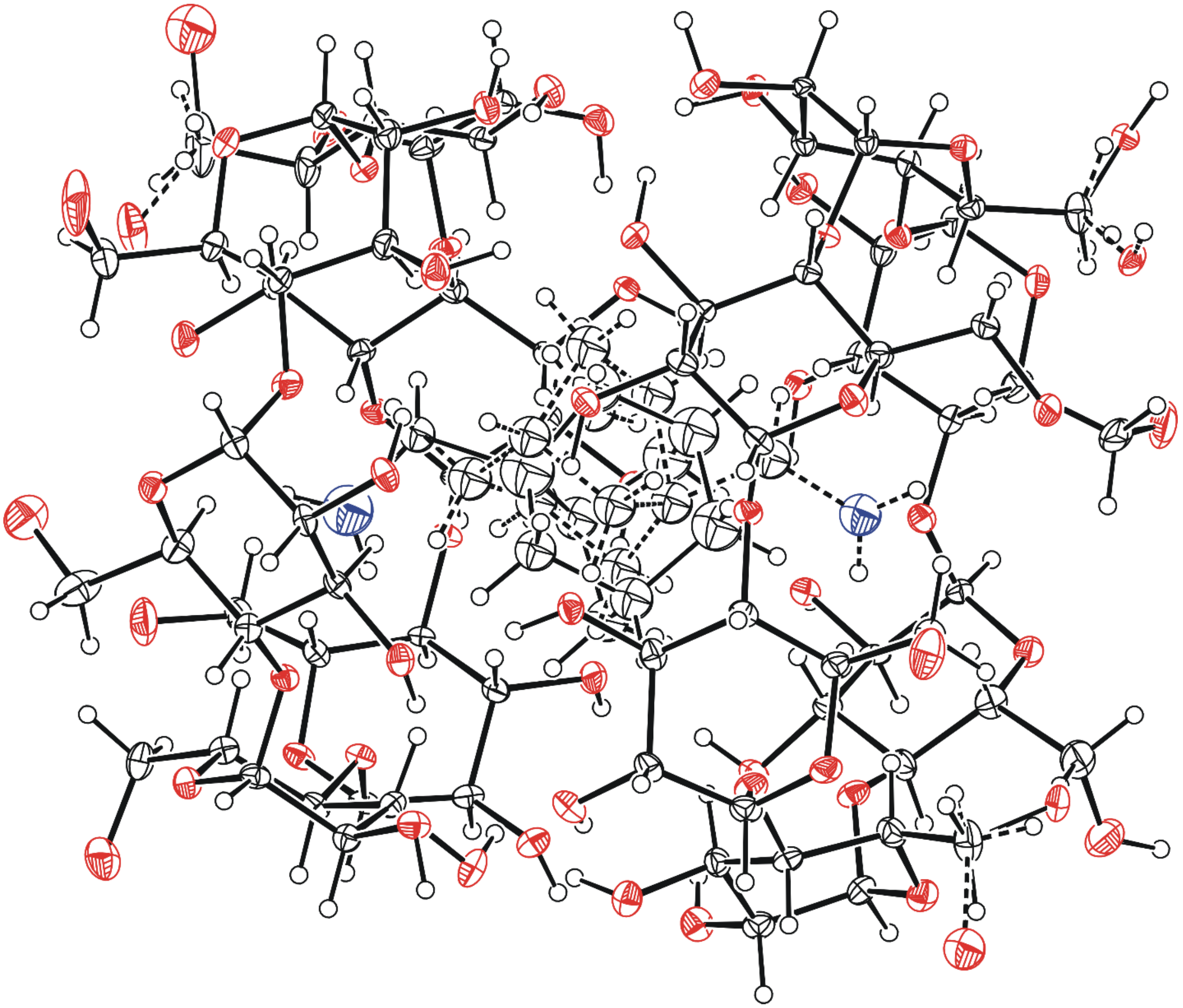

Figure S81 View on the dimer of alfa-cyclodextrin with the guest molecule noradamantane-3-methylene amine, the displacement ellipsoid are drawn on 30% probability level.

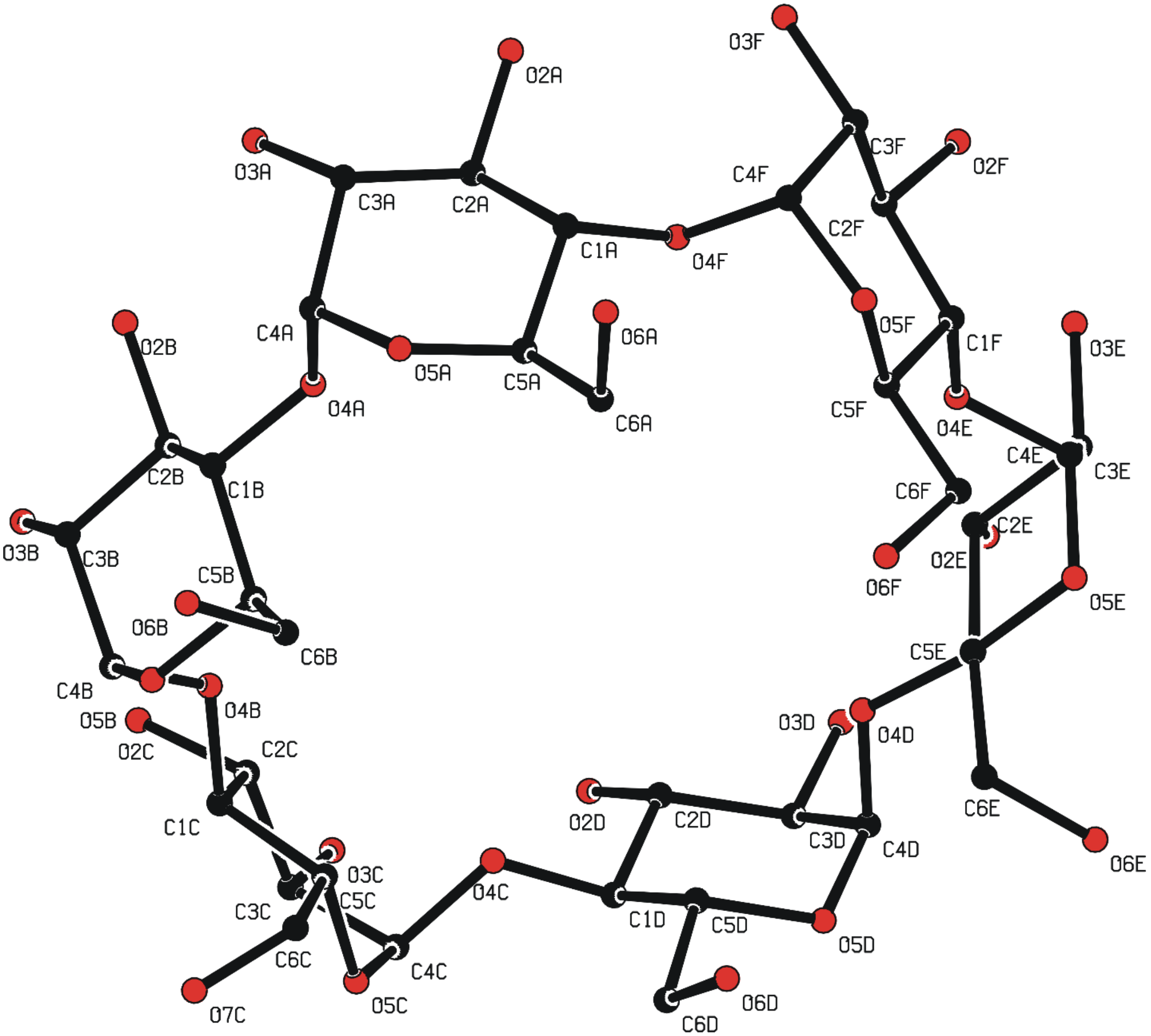

Figure S82 View on one alfa-cyclodextrin ring with the atom numbering schema.

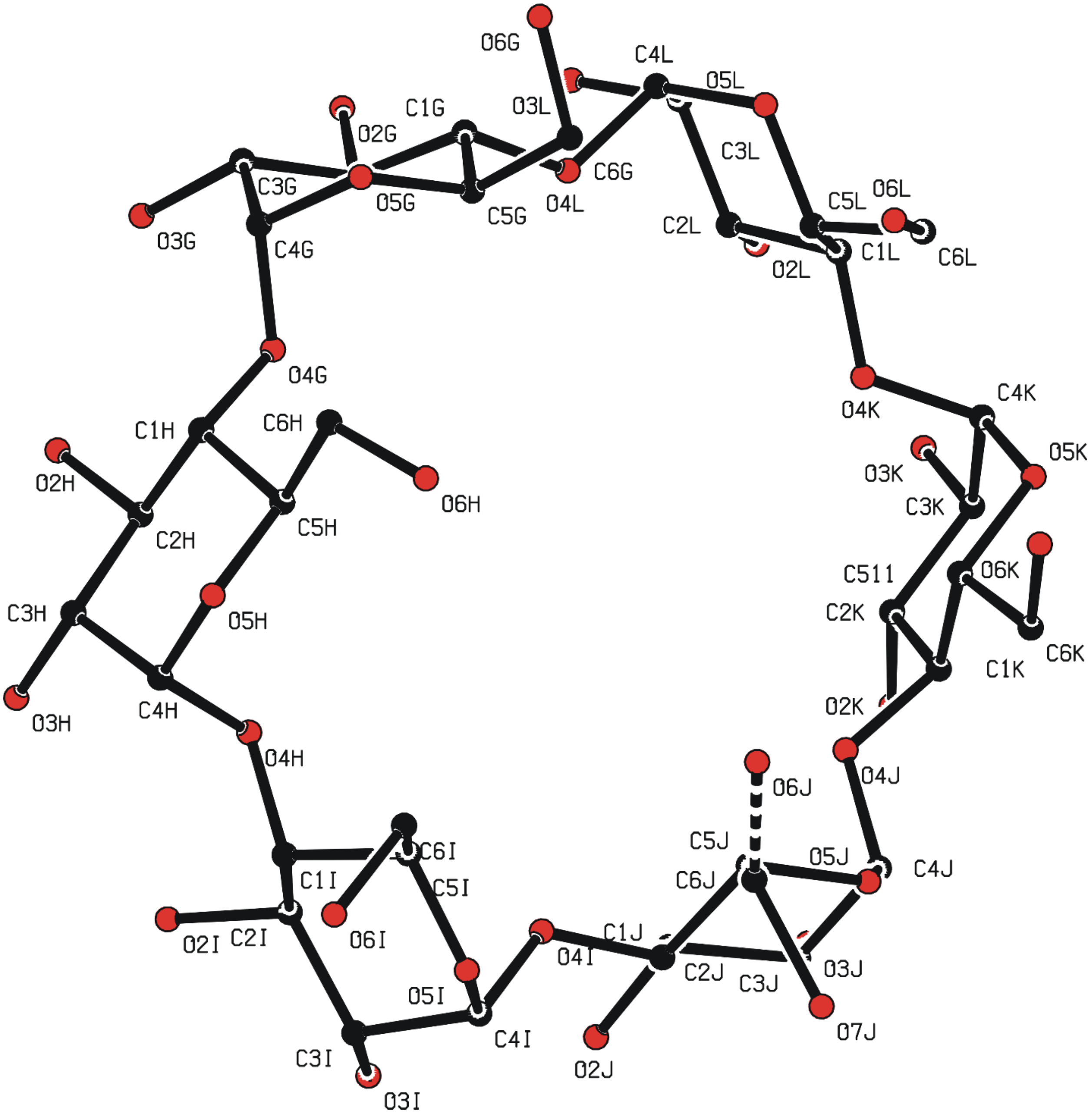

Figure S83 View on the second alfa-cyclodextrin ring with the atom numbering schema.

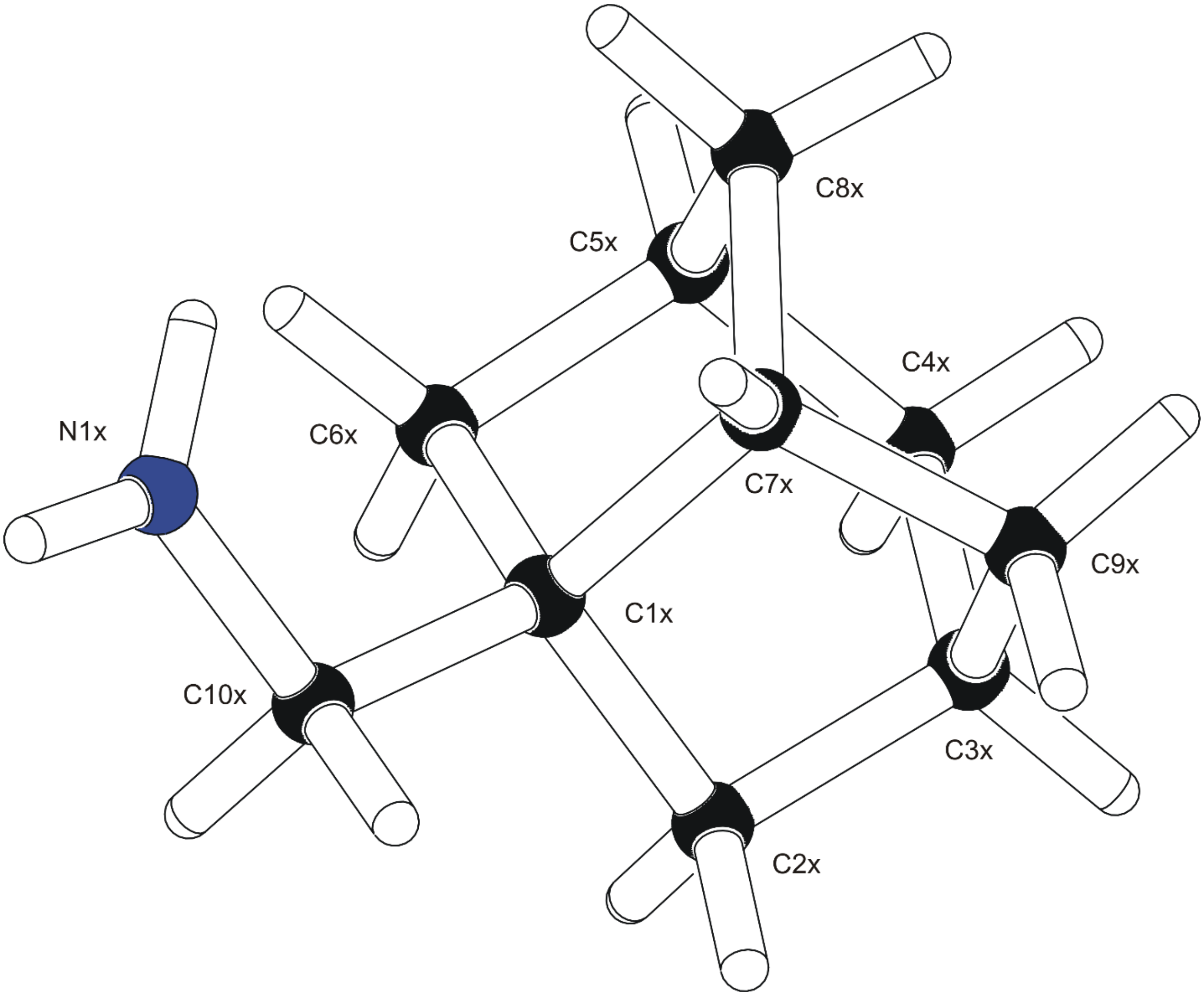

Figure S84 View on one orientation on noradamantane molecule with atom numbering schema.

REFERENCES

1) Cameron E. A., Maynard M. A., Smith C. J., Smith T. J., Koropatkin N. M., Martnes E. C. *J. Biol. Chem.* **2012,** *287,* 34614-34625; doi: 10.1074/jbc.M112.397380

2) Gebhardt J., Kleist C., Jakobtorweihen S., Hansen N. *J. Phys. Chem. B* **2018,** *122*, 1608-1626; doi: 10.1021/acs.jpcb.7b11808

3) Humphrey W., Dalke A., Schulten K. *J. Molec. Graphics* **1996,** *14,* 33-38; doi: 10.1016/0263-7855(96)00018-5

4) https://cgenff.umaryland.edu/

5) Phillips J. C., Braun R., Wang W., Gumbart J., Tajkhorshid E., Villa E., Chipot C., Skeel R. D., Kalé L., Schulten K. *J. Comput. Chem.* **2005,** *26,* 1781-1802; doi: 10.1002/jcc.20289

6) Phillips J. C., Hardy D. J., Maia J. D. C., Stone J. E., Ribeiro J. V., Bernardi R. C., Buch R., Fiorin G., Henin J., Jiang W., McGreevy R., Melo M. C. R., Radak B. K., Skeel R. D., Singharoy A., Wang Y., Roux B., Aksimentiev A., Luthey-Schulten Z., Kalé L. V., Schulten K., Chipot C., Tajkhorshid E. *J. Chem. Phys.* **2020,** *153,* 044130; doi: 10.1063/5.0014475

7) Roe D. R., and Cheatham III T. E. *J. Comput. Chem.* **2018,** *39,* 2110-2117; doi: 10.1002/jcc.25382

8) Pettersen E. F., Goddard T. D., Huang C. C., Couch G. S., Greenblatt D. M., Meng E. C., Ferrin T. E. *J. Comput. Chem.* **2004,** *25,* 1605-12; doi: 10.1002/jcc.20084

9) SHELXT: Sheldrick, G.M. (2015). *Acta Cryst*. **A71**, 3-8.

10) SHELXL: Sheldrick, G.M. (2015). *Acta Cryst.* **C71**, 3-8.

11) Parsons,S., Flack, H.D. and Wagner, T. *Acta Cryst*. **2013,** *B69,* 249-259.
